# Identification of an elusive spliceogenic *MYBPC3* variant in an otherwise genotype-negative hypertrophic cardiomyopathy pedigree

**DOI:** 10.1038/s41598-022-11159-y

**Published:** 2022-05-04

**Authors:** Mario Torrado, Emilia Maneiro, Arsonval Lamounier Junior, Miguel Fernández-Burriel, Sara Sánchez Giralt, Ana Martínez-Carapeto, Laura Cazón, Elisa Santiago, Juan Pablo Ochoa, William J. McKenna, Luis Santomé, Lorenzo Monserrat

**Affiliations:** 1 Cardiovascular Research Group, University of A Coruña, Campus de Oza, Building Fortín, 15006 A Coruña, Spain; 2 Biomedical Research Institute of A Coruña, A Coruña, Spain; 3 Cardiovascular Genetics, Health in Code, Business Center Marineda, Avenida de Arteixo 43, Local 1A, 15008 A Coruña, Spain; 4 Present Address: Medical School, Universidade Vale do Rio Doce, Governador Valadares, MG Brazil; 5 Genetics Unit, Hospital de Mérida, Badajoz, Spain; 6 Cardiology Department, Hospital de Mérida, Badajoz, Spain; 7 Institute of Cardiovascular Science, University College London, London, UK

**Keywords:** Cardiac hypertrophy, Gene expression, Disease genetics, Genetic testing

## Abstract

The finding of a genotype-negative hypertrophic cardiomyopathy (HCM) pedigree with several affected members indicating a familial origin of the disease has driven this study to discover causative gene variants. Genetic testing of the proband and subsequent family screening revealed the presence of a rare variant in the *MYBPC3* gene, c.3331−26T>G in intron 30, with evidence supporting cosegregation with the disease in the family. An analysis of potential splice-altering activity using several splicing algorithms consistently yielded low scores. Minigene expression analysis at the mRNA and protein levels revealed that c.3331−26T>G is a spliceogenic variant with major splice-altering activity leading to undetectable levels of properly spliced transcripts or the corresponding protein. Minigene and patient mRNA analyses indicated that this variant induces complete and partial retention of intron 30, which was expected to lead to haploinsufficiency in carrier patients. As most spliceogenic *MYBPC3* variants, c.3331−26T>G appears to be non-recurrent, since it was identified in only two additional unrelated probands in our large HCM cohort. In fact, the frequency analysis of 46 known splice-altering *MYBPC3* intronic nucleotide substitutions in our HCM cohort revealed 9 recurrent and 16 non-recurrent variants present in a few probands (≤ 4), while 21 were not detected. The identification of non-recurrent elusive *MYBPC3* spliceogenic variants that escape detection by in silico algorithms represents a challenge for genetic diagnosis of HCM and contributes to solving a fraction of genotype-negative HCM cases.

## Introduction

The first splice-altering variants identified in the myosin binding protein C3 gene (*MYBPC3*) were reported as early as 1995^1,2^, two years before the first characterization of the structure and sequence of the human *MYBPC3* gene^3^. Two single-nucleotide substitutions were identified, with the current nomenclature names c.1928−2A>G^1^ and c.3330+5G>C^2^, that independently segregated with hypertrophic cardiomyopathy (HCM) in different families. Splicing assays using total RNA isolated from patients’ lymphocytes revealed the presence of aberrant misspliced *MYBPC3* transcripts in affected carriers. These splicing defects are predicted to create a downstream frameshift and a premature termination codon in the sequence of the *MYBPC3* mRNA. In particular, the c.1928−2A>G variant suppresses the splice acceptor site of intron 20, leading to misspliced transcripts with skipping or truncation of exon 21, whereas the c.3330+5G>C variant is located in intron 30 and causes skipping of exon 30.

At times, the term cryptic splice-altering variant is used, which we refer to and define as a small genetic alteration, usually a single-nucleotide substitution, located outside the essential GT or AG dinucleotides of the canonical splice donor and acceptor sites at positions + 1/ + 2 and − 2/ − 1, respectively, that disrupts the normal pre-mRNA splicing. Cryptic splice-altering variants may be located not far away from the canonical splice sites, at deep-intronic positions, or even in exonic sequences. The more general single-word term spliceogenic refers to a variant that alters the normal pre-mRNA splicing irrespective of its location within the gene.

The identification and characterization of these spliceogenic *MYBPC3* variants by the pioneer studies represented the first evidence that mutations in the *MYBPC3* gene cause familial hypertrophic cardiomyopathy. Mutations in *MYBPC3* are also the most common cause of the disease, and most pathogenic or likely pathogenic *MYBPC3* gene variations are heterozygous truncating alterations caused by nonsense, frameshift or splice-altering variants. Together with *MYBPC3*, the eight core sarcomeric genes (*MYBPC3*, *MYH7*, *ACTC1*, *MYL2*, *MYL3*, *TNNT2*, *TNNI3* and *TPM1*) accounted for more than 99% of variants classified as pathogenic or likely pathogenic by clinical-grade criteria for HCM testing, as revealed by combining clinical results from large HCM cohorts^4^. This large study also reported that clinical genetic testing of HCM patients frequently led to the identification of a pathogenic or likely pathogenic variant in the *MYBPC3* (15.1% of cases) or *MYH7* (9.8% of cases) genes, whereas in 60.1% of cases, no pathogenic or likely pathogenic variant was detected^4^. Other large-scale studies described similar results, and the relative contribution of each gene to all genotype-positive HCM cases were reported to be 50% for *MYBPC3* and 33% for *MYH7*, whereas the genotype-negative detection rate in the entire HCM cohort was 54%^5^. It was estimated that about 40–60% of genetically tested HCM cases had an inconclusive or negative test result^6^, and that expanded diagnostic gene panels offer limited additional sensitivity^5^, representing an important limitation of current personalized genetic diagnostics of one of the most common inherited heart diseases.

Hypertrophic cardiomyopathy is a clinically and genetically heterogeneous heart disease with a classic prevalence estimate of approximately 1/500 individuals, or 0.2% (reviewed in^7^). This prevalence was recently considered to be probably conservative^8^, and a revised estimate of about 1/200 has been proposed^7^, taking into account the combined prevalence of enhanced detection of the HCM phenotype by advanced imaging and carriers of disease-causing variants in the general population. As defined in current guidelines^9,10^, the clinical diagnosis of HCM in adult patients is established by cardiac imaging showing an otherwise unexplained maximal end-diastolic wall thickness of ≥ 15 mm anywhere in the left ventricle (LV). The clinical diagnosis in family members of a patient with HCM is based on the presence of otherwise unexplained increased LV wall thickness ≥ 13 mm in one or more LV myocardial segments. The clinical spectrum of HCM is diverse and includes, in most cases, asymptomatic or minimally symptomatic patients and subsets of gene-positive phenotype-negative individuals carrying a pathogenic variant but without left ventricular hypertrophy^11,12^. However, complications attributable to HCM may progress and lead to sudden death, which could occur in asymptomatic or mildly symptomatic patients^11^, and patients are at increased age-related risk for the development of adverse disease-related events dominated by heart failure and atrial fibrillation that develop later in life^13^.

Particularly in the last four years, cryptic splice-altering *MYBPC3* variants located in proximal, intermediate or distal intronic positions relative to the nearest splice site^14–19^, and also in exonic sequences^17,20^, have been increasingly recognized as a cause of HCM. Accordingly, it was suggested that massively parallel sequencing of the whole *MYBPC3* gene, including the complete sequence of introns, improves the yield and efficiency of molecular diagnosis of patients with HCM^15,16,19^. Usually, rare *MYBPC3* intronic variants that could be detected by massively parallel sequencing of a patient’s genomic DNA are prioritized for functional validation using deep-learning in silico splicing tools^17,21,22^.

In this work, we report the genetic and molecular characterization of *MYBPC3* c.3331−26T>G, a rare, previously undescribed intronic variant identified in a pedigree with a genetically unresolved cause of HCM. Despite the low priority assigned by in silico analyses, both minigene and patients’ RNA splicing assays indicated that the elusive single-nucleotide substitution c.3331−26T>G is actually a spliceogenic variant promoting complete and partial retention of intron 30 in *MYBPC3* misspliced transcripts. A frequency analysis was also performed as part of the characterization of this variant. We examined the frequency of *MYBPC3* c.3331−26T>G in a large HCM cohort, with the aim of identifying additional unrelated probands carrying this nucleotide substitution. The frequency analysis was also extended to include a comprehensive list of known cryptic splice-altering *MYBPC3* intronic variants in order to contribute to the recognition of recurrent and non-recurrent spliceogenic variants.

## Results

### Clinical characterization

**Figure 1:**
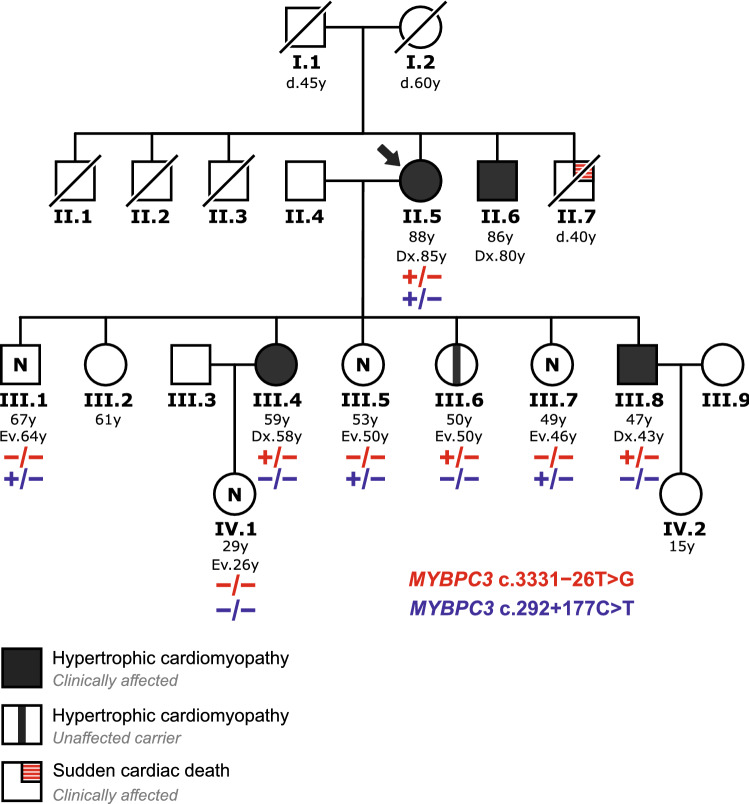
Family pedigree of the index patient. Family members diagnosed with HCM are shown with filled circles (females) or squares (males). Arrow indicates the index patient. The index patient's parents (I.1, I.2) died (d.) relatively young without known cardiac disease, and also three brothers of the index patient (II.1, II.2, II.3) have died of other causes without being known to have heart disease. A fourth brother (II.7) of the index patient has died of sudden death. A fifth brother (II.6) of the index patient has been diagnosed with moderate septal hypertrophy, but he could not be included in the genetic testing. In generations III and IV, two sons, four daughters and a granddaughter of the index patient were included in both the clinical evaluation and genetic testing. Massively parallel sequencing analysis of the index patient identified two rare *MYBPC3* intronic variants (see Supplementary Fig. S1). Family genetic testing results (see Fig. 2) of these two intronic variants are shown for each family member analyzed. At the last clinical evaluation, the index patient’s daughter III.6 did not meet the criteria for HCM, and she was considered an unaffected carrier of the *MYBPC3* c.3331−26T>G variant, according to further functional characterization of this intronic variant (see text). The age (years) of each family member is indicated, and also the age at diagnosis (Dx.) or last clinical evaluation (Ev.). N—unaffected, clinically evaluated.

The pedigree of the index patient indicating hypertrophic cardiomyopathy of familial origin is shown in Fig. 1. The index case was an 88-year-old woman (II.5) admitted to the emergency unit with chest pain after a pre-syncopal episode at the age of 85. Coronary artery disease was ruled out. Electrocardiogram showed sinus rhythm, negative T waves in V1-V3 and signs of ventricular hypertrophy. Transthoracic echocardiography detected asymmetric septal hypertrophy of 25 mm with preserved ejection fraction, moderate diastolic dysfunction and mild left atrial enlargement. Twenty-four-hour Holter monitoring documented episodes of non-sustained ventricular tachycardia defined as ≥ 3 beats at a rate of ≥ 120 bpm. An index patient’s brother (II.6) was reported with moderate HCM and another brother (II.7) with sudden cardiac death at the age of 40 years.

The finding of severe cardiac hypertrophy in the index patient together with the reported moderate hypertrophy and sudden death in her brothers prompted the clinical evaluation of the next-generation family members, none of whom had a prior diagnosis of heart disease at that time. The clinical screening led to the diagnosis of HCM in two of the index patient’s descendants, a daughter and a son. The 59-year-old daughter (III.4) was complaining of an unspecified chest pain; her cardiac magnetic resonance showed septal hypertrophy of 16 mm and electrocardiogram Q waves in V1-V3 and high voltages. The annual follow-up of this patient revealed a progressive annual hypertrophic increase, reaching the diagnostic criteria for HCM by cardiac magnetic resonance in the last evaluation. The 47-year-old son (III.8) was diagnosed with septal hypertrophy of 15 mm, without obstructive gradient. No malignant arrhythmia was identified in either of them. Five other relatives, one son (III.1), three daughters (III.5, III.6 and III.7) and a granddaughter (IV.1), were healthy individuals whose ECG, echocardiogram, 24 h-Holter and exercise treadmill test were normal. No heart disease was reported in the other family members.

### Genetic diagnosis of the index patient

The index patient’s genomic DNA was subjected to massively parallel sequencing to identify the genetic origin of the disease using a 251-gene cardiomyopathy diagnostic panel. Genetic data analysis returned a negative result for the presence of a definitive or likely pathogenic variant in the genes covered by the panel, including HCM sarcomeric, non-sarcomeric and syndromic genes (see Supplementary Table S1), that could explain the phenotype. Although this genotype-negative hypertrophic cardiomyopathy case is not unusual, as about half^6,23^ of patients tested have a negative genetic test result, in most adult probands, it is unlikely to identify pathogenic variants in genes not previously associated with HCM, as has been considered recently^6^. In this line, the genetic diagnosis of this case was focused on the discovery of causative variants in known HCM genes. After sorting the massively parallel sequencing data and filtering out common variants, two rare intronic single-nucleotide substitutions remained in the *MYBPC3* gene, located in intron 2 (NM_000256.3:c.292+177C>T) and intron 30 (NM_000256.3:c.3331−26T>G). As shown in Supplementary Fig. S1, the alignments of massively parallel sequencing reads to the reference genome revealed that the index patient carried these variants in intron 2 and 30 in the heterozygous state. Both intronic variants are expected to be extremely rare, since they have been neither described in the literature nor reported in large population databases, including the Single Nucleotide Polymorphism database (dbSNP) and the Genome Aggregation database (gnomAD). The c.292+177C>T variant (genomic name NC_000011.9:g.47372613G>A) is registered in dbSNP under the accession ID rs966994107, which covers the genomic changes G>T and G>A, with one and zero cases, respectively. As these were the only possible candidates, we decided to perform a detailed characterization of both variants and, consequently, used several deep learning-based algorithms to evaluate the probability of these two rare *MYBPC3* intronic variants of being splice-altering.

### In silico analysis of variant‐induced alterations of pre-mRNA splicing

The SpliceAI algorithm returned a near null value (Δ score = 0.01) for c.292+177C>T and a low score (Δ score = 0.16) for the c.3331−26T>G variant. These values are below the high recall threshold (Δ score ≥ 0.2) for higher sensitivity for detecting splice-altering variants (Supplementary Table S2). The Alamut software package also returned neutral or low score predictions for both variants (Supplementary Fig. S2). The output of both in silico splicing tools suggested that these variants are neither involved in the loss of a splice site nor in the gain of a recognizable new splice site.

The c.3331−26T>G variant is expected to be located within the branchpoint area of intron 30, since most (about 90%) human splicing branchpoints are located upstream of the polypyrimidine tract at relative positions within − 37 to − 19 nucleotides from the splice acceptor site, as revealed by a large-scale experimental annotation of human splicing branchpoints^24^. The branchpoint sequence is one of the sequence elements essential for splicing, and branchpoint recognition (frequently involving the nucleotide adenine in about 78% of introns^24,25^) by U2 snRNA is an early prespliceosome assembly event that initiates a two-step reaction, branching and exon ligation, finally responsible for intron removal from pre-mRNA and exon ligation to form mature mRNA (recently reviewed in^26^). Thus, the c.3331−26T>G variant was further analyzed with several deep-learning algorithms specifically designed for splice branchpoint annotation and prediction of the effects of intronic variants on branchpoint selection (Supplementary Table S3). According to the output of Branchpointer (see also Supplementary Fig. S3), the c.3331−26T>G variant is close to several potential weak branchpoints of intron 30 with the highest probability at − 31A, but the nucleotide substitution is not predicted to have a major effect on the selection of the branchpoint. All potential branchpoints identified in intron 30 reference or alternative sequences are below the recommended probability threshold to distinguish branchpoints and non-branchpoint sites.

We have also run Branchpointer with the input of a previously reported^18^ *MYBPC3* splice-altering variant, c.1898−23A>G, which escaped detection by SpliceAI (Δ score = 0.04), with the aim of obtaining a positive control for the in silico prediction. This variant is located in intron 19 at a similar relative position from the splice acceptor site. Branchpointer identified the branchpoint in intron 19 with a high score (0.705), located in the nucleotide A at relative position − 23, the same position affected by the variant, and the nucleotide substitution c.1898−23A>G significantly reduced the branchpoint probability (0.092) with the use of − 23G, and no other potential branchpoints emerged (Supplementary Fig. S3). This Branchpointer prediction confirmed the high probability of branchpoint disruption previously reported with the use of LaBranchoR^18^ and further pointed to branchpoint suppression with no other alternative branchpoint candidates. Both tools consistently provided computational evidence for a mechanism that would explain the induction of a misspliced transcript with retained intron 19 caused by the c.1898−23A>G variant.

In the case of *MYBPC3* intron 30, RNABP (see also Supplementary Fig. S4) and LaBranchoR also predicted several putative branchpoints with the highest probability at nucleotide positions − 31A (RNABP) and − 29A (LaBranchoR), and a minor score change at these positions associated with the c.3331−26T>G variant. The BPP software identified the branchpoint sequence of intron 30 as the heptamer GGCCCAG, which includes the branchpoint at − 31A (underlined). According to BPP, the nucleotide substitution c.3331−26T>G is located outside of this heptamer, with no expected effect on the selection of the branchpoint (see Supplementary Table S3).

Thus, the four branchpoint tools suggested that the *MYBPC3* c.3331−26T>G variant is close to several possible branchpoints but does not have a considerable effect on the scores obtained by the top-ranked nucleotides − 31A or − 29A which may correspond to the natural branchpoint of intron 30.

In summary, all in silico tools used for splicing defect prediction have assigned a low priority score to the *MYBPC3* intronic nucleotide substitutions c.292+177C>T and c.3331−26T>G for being spliceogenic variants.

### Family genetic testing for *MYBPC3* variants identified in the index patient

**Figure 2:**
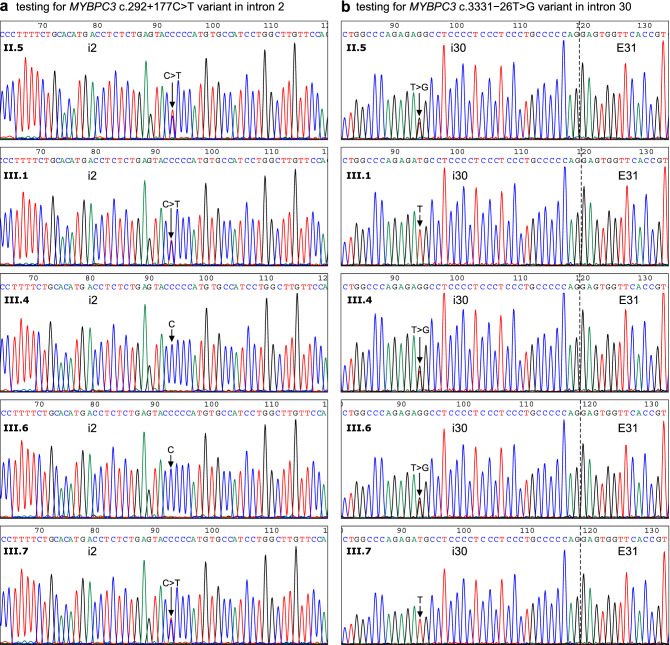
Family inheritance analysis of two rare intronic *MYBPC3* variants identified in the index patient. Sanger DNA chromatograms testing the *MYBPC3* c.292+177C>T variant in intron 2 (**a**) and the *MYBPC3* c.3311−26T>G variant in intron 30 (**b**) are shown. DNA chromatograms from the index patient (II.5) revealed the presence of both nucleotide substitutions in the heterozygous state, thus independently confirming the massively parallel sequencing results (see Supplementary Fig. S1). Sequence analyses of four family members of the III generation are also shown: one index patient’s son (III.1) and three daughters (III.4, III.6 and III.7). Two of them inherited from their mother the *MYBPC3* variant in intron 2 (III.1, III.7) and the other two (III.4, III6) the *MYBPC3* variant in intron 30. The complete family testing results are presented in Fig. 1. Arrows indicate the position of the affected nucleotide. The intron–exon boundary (i30-E31) is marked with a dotted line.

As a next step in the characterization of the intronic *MYBPC3* variants identified in the index patient, we performed a family genetic testing for phase determination and disease segregation analysis (Fig. 2). Sanger DNA sequencing of the index patient (II.5) confirmed the previous massively parallel sequencing results, and both variants were detected in the heterozygous state. All family members analyzed within generation III inherited from their mother (index patient II.5) one *MYBPC3* variant or another in the heterozygous state, but none inherited both variants, indicating that the index patient carried the intronic variants c.292+177C>T and c.3331−26T>G in trans, located on different alleles. As shown in the family pedigree (Fig. 1), the presence of the c.292+177C>T variant in three unaffected individuals (III.1, III.5 and III.7) and its absence in two affected patients (III.4 and III.8) suggested no association with the disease in the family. On the other hand, the presence of the c.3331−26T>G variant has been identified in two HCM patients from generation III, the index patient’s daughter (III.4) and son (III.8), but also in an unaffected index patient’s daughter (III.6). The genomic DNA from patient III.8 was also subjected to massively parallel sequencing analysis, which confirmed the presence of the nucleotide substitution c.3331−26T>G in the heterozygous state but has not identified other relevant variants. Affected non-carriers of c.3331−26T>G were not found among tested family members.

Family genetic testing for the *MYBPC3* c.3331−26T>G variant has provided supporting-level evidence (PP1^27,28^) for segregation with the disease in the index patient (II.5), her daughter (III.4) and son (III.8), taking into account that this variant is absent in large population cohorts of gnomAD, thereby meeting the moderate-level criterion PM2^27,28^. This interpretation was further supported by the emerging evidence of cosegregation of the *MYBPC3* c.3331−26T>G variant with the disease, revealed by a frequency analysis in our large cohort of HCM probands (see Table 1) leading to the identification of two unrelated HCM index cases, in addition to the family described here, carriers of the c.3331−26T>G variant without other relevant variants that could explain the phenotype. Disease expression heterogeneity in affected families is expected, since a number of *MYBPC3* variants associated with HCM have shown incomplete, age-related and gender specific penetrance—higher in males than in females^29^. The disease expression heterogeneity, with gender and age-related incomplete penetrance, would explain the absence of clinical expression in patient III.6 (age 50y), a female carrier of the *MYBPC3* c.3331−26T>G variant. In order to obtain further stronger functional evidence for the characterization of the *MYBPC3* c.3331−26T>G variant, we next performed an experimental RNA splicing assay by minigene expression.

**Table 1:** Frequency of cryptic splice-altering *MYBPC3* variants in a large cohort of HCM probands.

vID	GRCh38 NC_000011.10	GRCh37 NC_000011.9	Coding DNA NM_000256.3	L	HCM (n)	HCM (%)	SpliceAI Δ score	Splicing defect	Deduced effect on the TRF	Ref	dbSNP ID	ClinVar	ClinVar ID	gnomAD
v1	g.47351515C>T	g.47373066C>T	c.26−10G>A	i1	2	0.021	0.39	E2sk	In-frame (del 89 aa)	^21^	rs397515978	US	42,643	NA
v2	g.47350117G>A	g.47371668G>A	c.407−5C>T	i3	2	0.021	0.18	E4sk	In-frame (del 33 aa)	^21^	rs371312567	LB	414,443	4.24E−05
v3	g.47350009C>G	g.47371560C>G	c.505+5G>C	i4	1	0.010	0.86	E4tr	Frameshift—PTC	^47^	rs727503219	LP	164,151	NA
v4	g.47350008A>C	g.47371559A>C	c.505+6T>G	i4	0	0	0.85	E4sk	In-frame (del 33 aa)	^21^	rs397516055	US	42,762	NA
v5	g.47349769C>G	g.47371320C>G	c.654+5G>C	i5	1	0.010	0.62	E5tr	In-frame (del 16 aa)	^17^	rs397516066	US	263,572	NA
v6	g.47349769C>T	g.47371320C>T	c.654+5G>A	i5	1	0.010	0.50	E5sk	Frameshift—PTC	^21^	rs397516066	US	42,782	NA
v7	g.47348566T>C	g.47370117T>C	c.655−25A>G	i5	1	0.010	0.19	E6sk	Frameshift—PTC	^48^	NA	NA	NA	NA
v8	g.47347854C>A	g.47369405C>A	c.821+3G>T	i7	1	0.010	0.71	E7sk	Frameshift—PTC	^17^	rs727503213	US/LP/P	228,869	NA
v9	g.47347852C>T	g.47369403C>T	c.821+5G>A	i7	2	0.021	0.86	E7sk	Frameshift—PTC	^3,21^	rs397516077	US/LP/P	42,796	NA
v10	g.47347065C>T	g.47368616C>T	c.906−36G>A	i9	9	0.094	0.91	pRi9/E10ext	Frameshift—PTC	^16,49^	rs864622197	LP/P	219,660	NA
v11	g.47347037A>G	g.47368588A>G	c.906−8T>C	i9	0	0	0.12	E10sk	In-frame (ins 1 aa, del 2 aa)	^21^	rs370245341	LB	922,256	8.78E−06
v12	g.47346623T>C	g.47368174T>C	c.926+4A>G	i11	0	0	0.40	E11sk	In-frame (ins 1 aa, del 7 aa)	^21^	rs397516081	US	42,803	NA
v13	g.47346380G>T	g.47367931G>T	c.927−10C>A	i11	0	0	0.45	E12sk	Frameshift—PTC	^21^	rs201078659	LP	42,805	NA
v14	g.47346379C>T	g.47367930C>T	c.927−9G>A	i11	12	0.125	0.28	E12sk	Frameshift—PTC	^21,50,51^	rs397516083	P	42,807	9.21E−06
v15	g.47346378C>T	g.47367929C>T	c.927−8G>A	i11	1	0.010	0.11	pRi11/E12ext	Frameshift—PTC	^52^	rs1468812059	NA	NA	NA
v16	g.47345754G>A	g.47367305G>A	c.1090+453C>T	i12	0	0	0.56	crypEins in i12	Frameshift—PTC	^15,19^	rs2095893477	LP	870,077	NA
v17	g.47344199T>G	g.47365750T>G	c.1091−575A>C	i12	0	0	0.38	crypEins in i12	Frameshift—PTC	^15^	NA	NA	NA	NA
v18	g.47343342C>T	g.47364893C>T	c.1224−80G>A	i13	6	0.175	0.94	pRi13/E14ext	In-frame (ins 26 aa)	^16^	rs1025692267	LP	693,982	NA
v19	g.47343314C>T	g.47364865C>T	c.1224−52G>A	i13	12	0.125	0.99	pRi13/E14ext	Frameshift—PTC	^15,53^	rs786204336	LP/P	188,544	3.19E−05
v20	g.47343283T>C	g.47364834T>C	c.1224−21A>G	i13	0	0	0.90	pRi13/E14ext	Frameshift—PTC	^14,54^	NA	US	1,180,864	NA
v21	g.47343281C>T	g.47364832C>T	c.1224−19G>A	i13	15	0.156	0.81	pRi13/E14ext	Frameshift—PTC	^49^	rs587776699	LP	138,326	2.56E−05
v22	g.47343159G>T	g.47364710G>T	c.1227−14C>A	i14	0	0	0.43	E15sk	Frameshift—PTC	^21^	rs574567658	NA	NA	4.20E−06
v23	g.47343158C>T	g.47364709C>T	c.1227−13G>A	i14	8	0.083	0.98	pRi14/E15ext	Frameshift—PTC	^21,55^	rs397515893	US/P	42,513	1.48E−05
v24	g.47343154G>T	g.47364705G>T	c.1227−9C>A	i14	0	0	0.08	E15sk	Frameshift—PTC	^21^	rs11570079	US	921,244	4.17E−06
v25	g.47342827C>G	g.47364378C>G	c.1457+3G>C	i16	0	0	0.25	E16sk	Frameshift—PTC	^21^	rs112918360	NA	NA	NA
v26	g.47342825C>G	g.47364376C>G	c.1457+5G>C	i16	1	0.010	0.93	E16sk	Frameshift—PTC	^14,21^	rs727503202	US	164,116	NA
v27	g.47342574T>A	g.47364125T>A	c.1624+4A>T	i17	3	0.031	0.16	E17sk	Stop gain—PTC	^14,17,21,51^	rs397515916	P	42,556	1.33E−05
v28	g.47342163G>T	g.47363714G>T	c.1625−7C>A	i17	0	0	0.11	E18sk	Frameshift—PTC	^21^	rs760972720	NA	NA	1.07E−05
v29	g.47341986C>T	g.47363537C>T	c.1790+5G>A	i18	0	0	0.89	E18sk	Frameshift—PTC	^21^	rs727504489	US	178,851	NA
v30	g.47341133C>T	g.47362684C>T	c.1897+5G>A	i19	0	0	0.77	E19sk	Frameshift—PTC	^21^	rs397515936	US	42,583	NA
v31	g.47341055T>C	g.47362606T>C	c.1898−23A>G	i19	4	0.042	0.04	cRi19	Frameshift—PTC	^18^	rs1158983908	NA	NA	5.01E−06
v32	g.47340403G>A	g.47361954G>A	c.1927+600C>T	i20	7	0.204	0.13	crypEins in i20	Frameshift—PTC	^16^	rs1595845204	P	692,114	NA
v33	g.47340359C>A	g.47361910C>A	c.1928−569G>T	i20	0	0	0.23	crypEins in i20	Frameshift—PTC	^19^	rs1343555574	NA	NA	NA
v34	g.47338682G>C	g.47360233G>C	c.2149−3C>G	i22	1	0.010	0.61	E23sk	Frameshift—PTC	^21^	rs113182334	NA	NA	NA
v35	g.47338517C>G	g.47360068C>G	c.2308+3G>C	i23	0	0	0.74	E23sk	Frameshift—PTC	^56^	NA	NA	NA	NA
v36	g.47337820T>C	g.47359371T>C	c.2309−26A>G	i23	0	0	0.35	E24sk	In-frame (del 35 aa)	^3^	rs886041030	P	8610	NA
v37	g.47337615C>A	g.47359166C>A	c.2414−36G>T	i24	0	0	0.52	pRi24/E25ext	Frameshift—PTC	^48^	rs371970979	NA	NA	8.07E−06
v38	g.47335212G>C	g.47356763G>C	c.2738−3C>G	i26	0	0	0.61	E27sk	Stop gain—PTC	^21^	NA	NA	NA	NA
v39	g.47335037C>A	g.47356588C>A	c.2905+5G>T	i27	0	0	0.96	E27sk	Stop gain—PTC	^21^	rs193922381	US/LP	42,668	NA
v40	g.47333552C>T	g.47355103C>T	c.3190+5G>A	i29	10	0.104	0.85	E29sk	Frameshift—PTC	^21,50^	rs587782958	P	155,808	1.68E−05
v41	g.47333189C>A	g.47354740C>A	c.3330+5G>T	i30	0	0	0.25	E30sk	Frameshift—PTC	^21^	rs373746463	P/LP	42,707	NA
v42	g.47333189C>G	g.47354740C>G	c.3330+5G>C	i30	14	0.146	0.30	E30sk	Frameshift—PTC	^2,21^	rs373746463	P/LP	42,706	2.71E−05
v43	g.47333189C>T	g.47354740C>T	c.3330+5G>A	i30	1	0.010	0.25	E30sk	Frameshift—PTC	^21^	rs373746463	P	8602	7.74E−06
v44	g.47332999A>C	g.47354550A>C	c.3331−26T>G	i30	3	0.031	0.16	cRi30	Frameshift—PTC	^This study^	NA	NA	NA	NA
v45	g.47332705G>C	g.47354256G>C	c.3491−3C>G	i31	4	0.042	0.90	E32sk	Frameshift—PTC	^17^	rs730880592	LP/US	181,010	NA
v46	g.47332270G>C	g.47353821G>C	c.3628−12C>G	i32	0	0	0.95	E33sk	Frameshift—stop loss	^21^	rs371428751	NA	NA	4.02E−06

vID—ID number assigned to each variant. Genomic (GRCh38 and Grch37 assembly versions) and coding DNA Reference Sequences to name the variants are indicated. L—variant location, intron (i) number. HCM—number (n) and percentage (%) of HCM probands in the HIC cohort database carrying each variant. HCM percentages for variants located up to 50–60 nucleotides from the splice site, are relative to 9,611 HCM probands tested by massively parallel sequencing in which intronic flanking regions were covered; HCM percentages for deep intronic variants are relative to 3,437 HCM probands tested by massively parallel sequencing in which all introns of *MYBPC3* were covered. Twenty-five variants were identified in 122 probands (sum of all n values) with HCM. With a few exceptions, most variants were detected only in HCM probands but not in patients with other inherited cardiovascular diseases (12,112 probands with intronic flanking regions sequenced, and 4,505 probands with complete *MYBPC3* intronic regions sequenced). Six variants were also detected in seven patients with other phenotypes, as follows: v1 was detected also in a case of Marfan syndrome; v2 in two cases of sudden cardiac death and dilated cardiomyopathy/myocarditis, respectively; v14 was detected in a patient with hyperlipoproteinemia; v19 and v32 in two cases of sudden cardiac death; and v42 in one case of dilated cardiomyopathy.

*SpliceAI Δ score* maximum SpliceAI Δ score, *Splicing defect* aberrant transcript structure reported in the corresponding references (Ref), the affected Exon (E) or intron (i) number is indicated, *tr* truncated, *sk* skipped, *ext* extended, complete (cRi) or partial (pRi) retention of intron, *crypEins* cryptic exon insertion, *TRF* translational reading frame, *PTC* premature termination codon, *aa* amino acids, *del* deletion, *ins* insertion, *dbSNP ID* accession number in dbSNP, *NA* not available, *ClinVar* ClinVar interpretation category, *LB* likely benign, *P* pathogenic, *LP* likely pathogenic, *US* uncertain significance, *ClinVar ID* accession number in ClinVar, *gnomAD* frequency of the alternative allele in gnomAD.

### *MYBPC3* minigene expression analysis at the mRNA level

**Figure 3:**
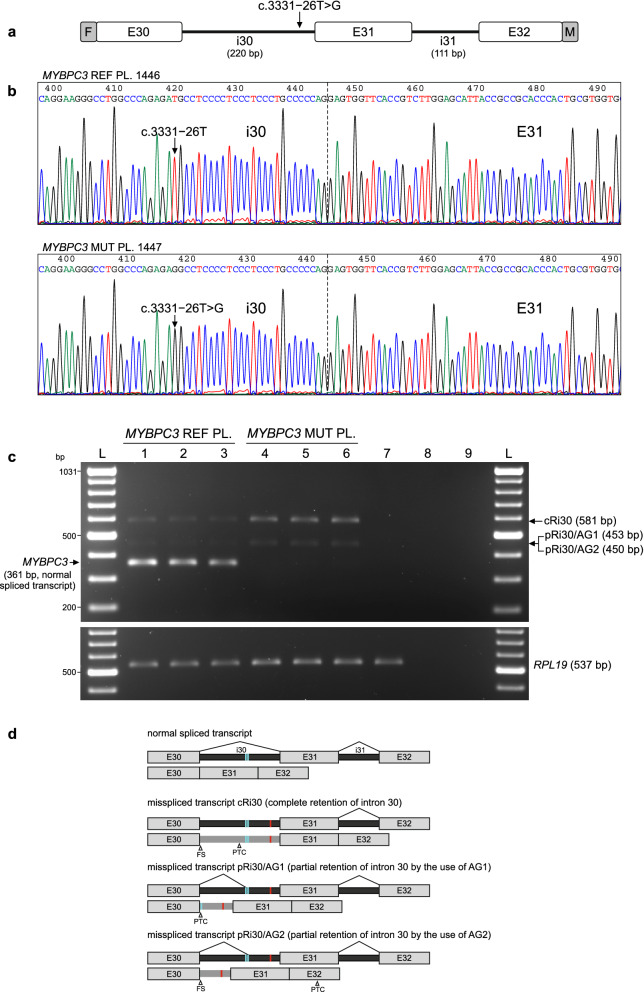
mRNA expression analysis of HeLa cells transfected with *MYBPC3* c.3331−26T>G minigenes. (**a**) Structure of the 767-bp *MYBPC3* minigene cloned in-frame to both epitope tags, 3XFLAG (F) and Myc-tag (M). Exons (E30, E31 and E32) and introns (i30 and i31) are represented with white boxes and black lines, respectively. Location of the variant in i30 is indicated. (**b**) Sanger DNA sequencing chromatograms of reference (REF, PL. 1446) and mutant (MUT, PL. 1447) plasmids carrying a T (REF) or alternative G (MUT) nucleotide corresponding to the *MYBPC3* c.3331−26T>G variant that were used for transfection experiments. The reference and mutant *MYBPC3* minigenes were generated by molecular cloning of the allelic copy of genomic DNA isolated from patient III.8, heterozygous carrier of the c.3331−26T>G variant, as described in the Methods section. Full insert DNA sequencing of REF and MUT plasmids are presented in Supplementary Figs. S5 and S6, respectively. (**c**) RT-PCR analysis of HeLa cells transiently transfected with *MYBPC3* REF or MUT plasmids. Samples were derived from the same experiment and processed in parallel. Agarose gel electrophoresis of RT-PCR products from triplicate transfections is shown. The lower, most abundant, 361-bp bands of the REF lanes (1–3) correspond to normal *MYBPC3* mRNA (see Fig. 4a). Normal *MYBPC3* mRNA was not detected in the MUT lanes (4–6), but two other larger bands were detected. In the MUT lanes, the longer bands (581 bp) correspond to a misspliced transcript with the complete retention of intron 30 (cRi30, see Fig. 4b). Sequence analysis of the intermediate-sized weaker band of about 450 bp revealed that it actually contained two transcripts, with partial retention of intron 30, named pRi30/AG1 (453 bp) and pRi30/AG2 (450 bp) (see Supplementary Fig. S8). Control samples were loaded in lane 7 (non-transfected cells), lane 8 (RT −) and lane 9 (non-template). A DNA ladder was loaded in lanes L. Endogenous *RPL19* was amplified as a control for the synthesis of cDNA. Full-size RT-PCR gel image is shown in Supplementary Fig. S7. (**d**) Schematic representation of normal spliced and the three misspliced transcripts. Location of c.3331−26T>G (red line) and the two preexisting cryptic acceptor sites AG1 and AG2 (cyan lines) in intron 30 are indicated. *PTC* premature termination codon, *FS* frameshift.

**Figure 4:**
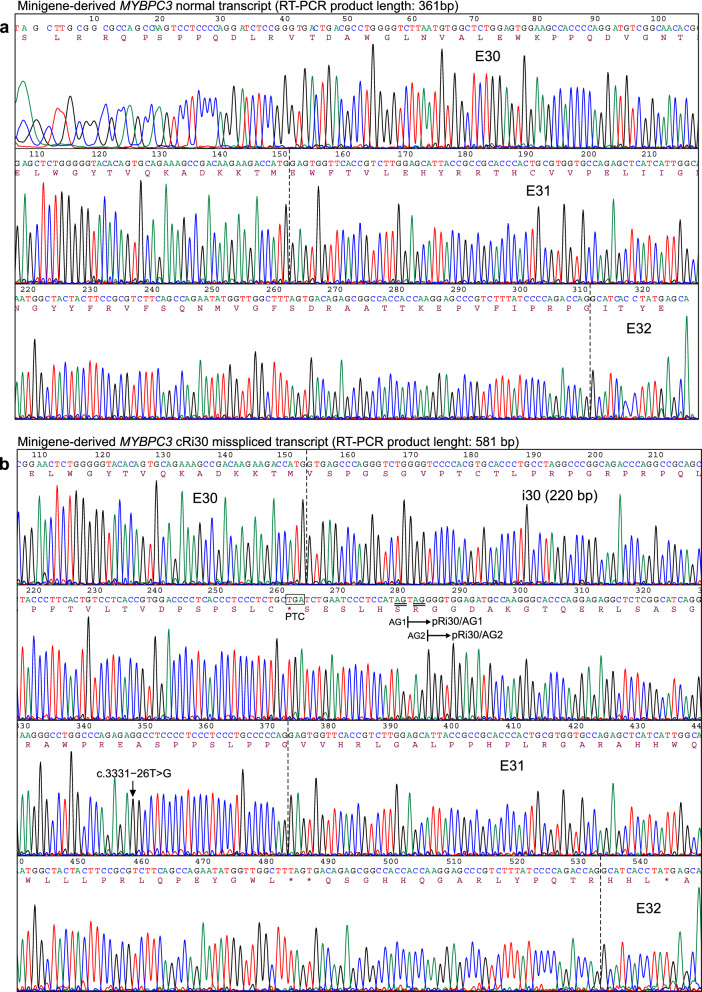
Sanger sequencing of minigene-derived normal and misspliced *MYBPC3* mRNA forms. RT-PCR products of HeLa cells transfected with REF or MUT *MYBPC3* c.3331−26T>G minigenes (as described in Fig. 3c) were eluted from agarose gel slices and subjected to Sanger sequencing. Sequencing chromatograms of the expected size (**a**, 361 bp, derived from the REF *MYBPC3* minigene) and the longer (**b**, 581 bp, derived from the MUT *MYBPC3* minigene) RT-PCR bands are shown. The sequence of the 361-bp product corresponds to properly spliced *MYBPC3* transcript, with exons (E30-E31-E32) normally ligated and introns (i30 and i31) removed. Sequence analysis of the longer 581-bp band revealed that it corresponds to a misspliced transcript with the complete retention of i30 (cRi30), but not i31. The presence of the *MYBPC3* c.3331−26T>G nucleotide substitution in the cRi30 misspliced transcript is indicated with a vertical arrow. The sequence analysis of the intermediate-sized (450 bp) RT-PCR band is shown in Supplementary Fig. S8, and revealed that two consecutive AG dinucleotides (named AG1 and AG2, double underlined in the cRi30 sequence), located in the middle of i30, were used as cryptic acceptor sites to generate two misspliced transcripts with partial retention of the second half of i30, named pRi30/AG1 and pRi30/AG2 (horizontal arrows). *PTC* premature termination codon.

The consequences of the *MYBPC3* c.3331−26T>G variant on pre-mRNA splicing were evaluated at the mRNA level by RT-PCR analysis of HeLa cells transiently transfected with reference 1446 (REF 1446, Supplementary Fig. S5) or mutant 1447 (MUT 1447, Supplementary Fig. S6) *MYBPC3* plasmids, carrying the reference T or the alternative G nucleotide, respectively, corresponding to nucleotide substitution c.3331−26T>G. As shown in Fig. 3 and Supplementary Fig. S7, an RT-PCR product of expected normal size (361 bp) was detected in cells transfected with the REF minigene as the most abundant transcript. Sanger sequencing confirmed that the 361-bp band corresponded to normal *MYBPC3* mRNA, with exons 30–31-32 ligated and intervening introns 30–31 removed (Fig. 4a). Two other very faint bands of larger size were noted in the REF lanes. In a sharp contrast, normal *MYBPC3* mRNA was not detected in the MUT lanes, but two other larger bands were detected. In the MUT lanes, the most abundant longer band of 581 bp corresponds to a misspliced transcript with the complete retention of intron 30 (named cRi30), but not intron 31. The Sanger DNA chromatogram of the *MYBPC3* cRi30 misspliced transcript is shown in Fig. 4b.

Sequence analysis of the intermediate-sized RT-PCR weaker band of 450 bp, identified in the MUT lanes, revealed that it actually contained two similar‐sized transcripts, which differed in a few nucleotides in the middle of the sequence (Supplementary Fig. S8). The forward Sanger chromatogram started as a single sequence through the whole E30, but it turned into a double overlapping sequence after the last ATG codon of E30. The reverse complement sequence, obtained by sequencing in the opposite direction, ended as a single sequence through the whole E31 and second half of intron 30, and then turned into double overlapping chromatogram peaks in the middle of intron 30. Detailed sequence analysis revealed that both sequences were generated by partial retention of the second half of intron 30 (pRi30). In these two aberrant transcripts, the first half of intron 30 has been removed from the pre‐mRNA by the use of the canonical GT dinucleotide splice donor site of intron 30 and the alternative use of two consecutive AG dinucleotides present in the middle of intron 30, named AG1 and AG2 (see Fig. 4b), acting as cryptic splice acceptor sites. The alternative use of cryptic acceptor sites AG1 or AG2 (the latter located three nucleotides downstream) generated two transcripts, named pRi30/AG1 and pRi30/AG2. Therefore, both misspliced transcripts with partial retention of intron 30 were amplified generating a single RT-PCR band, which actually contained both the 453‐bp pRi30/AG1 and 450‐bp pRi30/AG2 RT‐PCR products.

In the REF lanes, we were able to perform direct sequencing of the faint 581-bp band, revealing that it corresponded to the mRNA with complete intron 30 retention. Both minor bands (581 bp and 450 bp) from the REF lanes were interpreted as leaky expression resulting from the simplified genetic information of the reference minigene, although sufficient for processing normal *MYBPC3* mRNA as the most abundant RT-PCR band (361 bp). This leaky expression of minor missplicing events in the reference minigene could reflect a weakness of intron 30 for being recognized and removed with complete efficiency (for example caused by the presence of weak or/and competitive splice signals), which is exploited by the c.3331−26T>G variant leading to undetectable expression of the normal transcript by the mutant minigene and only missplicing events are detected.

Full-length sequence translation analysis of the misspliced transcripts identified by minigene assays predicted that the three of them (cRi30, pRi30/AG1 and pRi30/AG2) introduced a premature termination codon (PTC) into the *MYBPC3* mRNA, either as a result of downstream frameshift or a stop gain in the retained sequence (Supplementary Fig. S9). The complete retention of i30 in the aberrant transcript cRi30 introduces a PTC in the middle of i30. The partial retention of i30 in the misspliced transcript pRi30/AG1 introduces a PTC immediately after E30, whereas the PTC in pRi30/AG2 was found in E32, after translation of the second half of i30 and the out‐of‐frame translation of E31 and part of E32. Thus, the three misspliced transcripts (cRi30, pRi30/AG1 and pRi30/AG2, standardized nomenclature is given in Supplementary Table S4) identified by the minigene splicing assay were predicted to introduce a PTC before reaching the now abolished natural stop codon (which is located in the penultimate E34), with at least three downstream exon‐exon junctions. Therefore, it is expected that the three misspliced transcripts would be targets of nonsense-mediated mRNA decay, promoting its degradation and preventing the synthesis of aberrant truncated proteins, suggesting that this intronic variant would cause haploinsufficiency in carrier patients.

In summary, analysis of minigene expression at the mRNA level indicated that *MYBPC3* c.3331−26T>G is an elusive spliceogenic variant that escaped detection by deep-learning algorithms but that actually promotes disruption of pre-mRNA splicing with no detectable normal mRNA due to a failure to recognize and remove intron 30. To confirm these results at the protein level, minigene-derived MYBPC3-tag fusion proteins were analyzed by Western blotting.

### *MYBPC3* minigene expression analysis at the protein level

**Figure 5:**
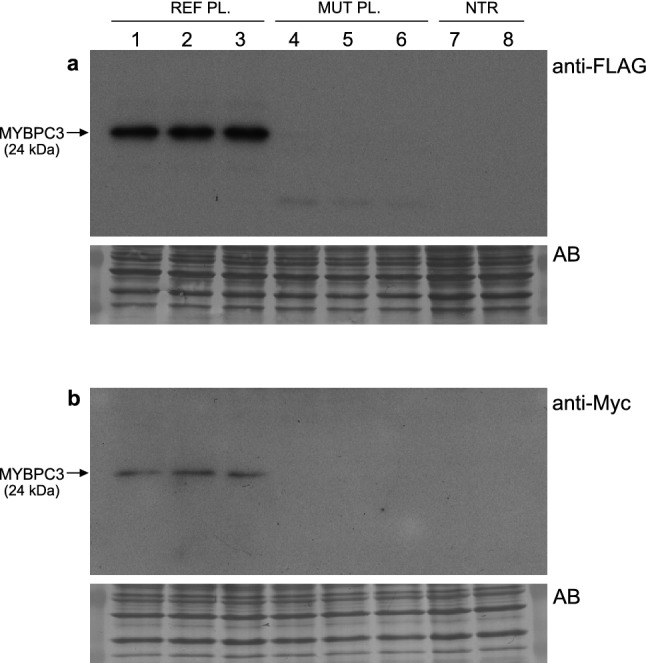
Western blot analysis of fusion proteins derived from *MYBPC3* c.3331−26T>G minigenes. *MYBPC3* reference (REF, Plasmid 1446) or mutant (MUT, Plasmid 1447) minigenes were transiently transfected into HeLa cells in triplicate as indicated on the top of each lane. Cell lysates derived from the same experiment and processed in parallel were analyzed by Western blot to detect the presence of FLAG-MYBPC3 and MYBPC3-Myc fusion proteins with monoclonal anti-FLAG (**a**) or anti-Myc (**b**) antibodies. Cells transfected with the REF plasmid expressed the expected normal MYBPC3 fusion protein of about 24 kDa encoded by the minigene, detected with both anti-tag (FLAG and Myc) monoclonal antibodies. The corresponding normal MYBPC3 fusion protein was not detected with either of the two antibodies in cells transfected with the MUT plasmid. A very faint band of about 16 kDa could be observed only in the MUT lanes with anti-FLAG, but not with anti-Myc, which may correspond to a poorly translated product from the misspliced cRi30 transcript. Each PVDF membrane was stained for total protein with Amido Black (AB) after immunostaining. Full size Western blot film and membrane images including the protein ladder used are presented in Supplementary Fig. S10. The anti-FLAG Western blot film displayed a strong immunological signal for the MYBPC3 fusion protein (containing three consecutive FLAG epitopes at the N-terminal protein end for increased detection sensitivity) encoded by the REF minigene, even at the lowest film exposure of about three seconds shown here. Similar results displaying weaker bands were obtained in an additional experiment using a lower amount of the anti-FLAG antibody, also presented in Supplementary Fig. S10 including different film exposures. *NTR* non-transfected cells.

Protein extracts from HeLa cells transiently transfected with REF or MUT minigenes in a second transfection experiment were analyzed by Western blot with anti-FLAG and anti-Myc monoclonal antibodies, targeting the N-terminal and C-terminal tags, respectively. The Western blot analysis with anti-FLAG (Fig. 5a, Supplementary Fig. S10) revealed the expected band of about 24 kDa corresponding to the normal MYBPC3-fusion protein encoded by the REF minigene (Fig. 5a, lanes 1–3). Again in a sharp contrast, the corresponding normal FLAG-MYBPC3 fusion protein was not detected in cells transfected with the MUT minigene (Fig. 5a, lanes 4–6). In addition, the Western blotting with anti-Myc tag (Fig. 5b) detected the normal protein synthesized by cells transfected with the REF minigene, confirming that the protein product is positive for both N-terminal FLAG and C-terminal Myc tags, and thus translated from the normal mRNA with the preserved reading frame. On the other hand, the Western blotting with anti-Myc also confirmed that cells transfected with the MUT minigene did not express detectable levels of either the normal protein or any other protein product with the preserved reading frame.

Taken together, the minigene splicing assay results indicated that the c.3331−26T>G variant produces a major disruption of pre-mRNA splicing, leading to no detectable amount of properly spliced transcript or normal protein product in transfected cells, representing strong evidence of pathogenicity (PS3), given that MYBPC3 loss of function and haploinsufficiency is a recognized mechanism in HCM^6^.

### Analysis of *MYBPC3* expression in patients’ blood

We next addressed whether the misspliced transcripts by the complete and partial retention of intron 30, identified by the minigene splicing assay, could be detected in the patients’ mRNA . Thus, in an independent approach, we have studied the functional splicing consequences of the *MYBPC3* c.3331−26T>G variant by analysis of ectopic expression of *MYBPC3* in blood samples from several family members. Although we could not obtain a valid blood sample from the index patient to be used in RNA assays, we have collected four blood samples from other family members, two from carriers (III.4 and III.6) and another two from non-carriers (III.1 and III.7) to be used as controls (Supplementary Fig. S11). The four of them were a son and daughters of the index patient.

**Figure 6:**
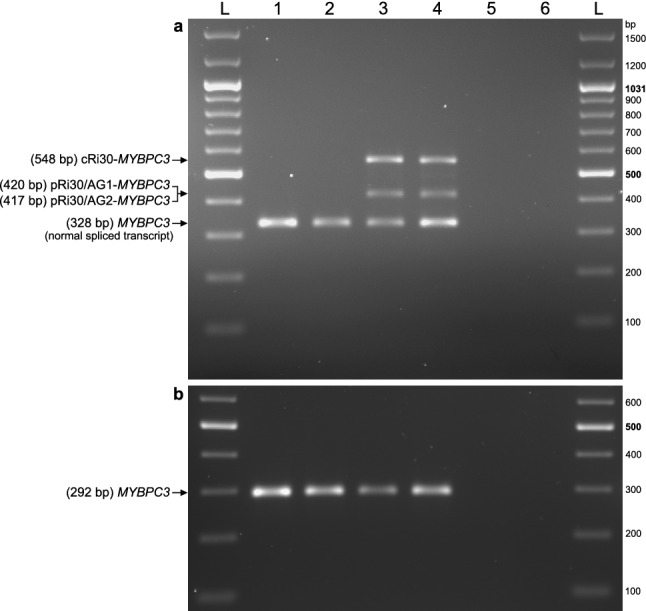
RT‐PCR analysis of *MYBPC3* expression in patients’ blood. (**a**) Agarose gel electrophoresis of RT‐PCR products of blood RNA isolated from non‐carriers (lane 1‐III.7, lane 2‐III.1) and carriers (lane 3‐III.4, lane 4‐III.6) of the *MYBPC3* c.3331−26T>G variant in intron 30. This splicing assay targeted the *MYBPC3* mRNA region between exons 30–31 using the primer pair 543–542. Negative controls were loaded in lane 5 (RT −) and 6 (non-template). The lower bands (328 bp) correspond to normal *MYBPC3* mRNA (Supplementary Fig. S13). The longer bands from both III.4 and III.6 correspond to a misspliced transcript with the complete retention of intron 30 (548 bp, cRi30‐*MYBPC3*, Supplementary Fig. S13). Sequence analysis revealed that the intermediate‐sized RT‐PCR bands actually contained two transcripts, with partial retention of intron 30 (Supplementary Fig. S14), named pRi30/AG1 (420 bp) and pRi30/AG2 (417 bp), as previously shown by the minigene expression assay (see Fig. 3). (**b**) An additional RT-PCR assay targeting the *MYBPC3* mRNA region between exons 2–3 with the primer pair 601–603 and the same cDNA samples was performed. This second assay was designed to analyze mRNA splicing in the presence of the *MYBPC3* c.292+177C>T variant, located in intron 2, in carriers (lane 1‐III.7, lane 2‐III.1) and non-carriers (lane 3‐III.4, lane 4‐III.6). A single RT-PCR band of the expected size (292 bp) was detected in all lanes. Sanger sequencing of the purified 292-bp band confirmed that it corresponds to normal *MYBPC3* mRNA (Supplementary Fig. S15). Full‐size RT‐PCR images are presented in Supplementary Fig. S12. L—DNA ladder.

*MYBPC3* RT-PCR amplification of total RNA from non-carrier controls (III7, III.1) produced a single band of the expected size (328 bp, lanes 1–2 in Fig. 6a, Supplementary Fig. S12). Sequencing of these purified RT-PCR products confirmed that they corresponded to the normal transcript, with exons 30 and 31 joined and intervening introns removed (Supplementary Fig. S13). In contrast, the RT-PCR of total RNA from the two carriers of the intronic variant c.3331−26T>G (III.4, III6, lanes 3–4 in Fig. 6a) showed two additional longer bands of 548 bp and 420 bp. Sanger sequencing of the 548-bp RT-PCR band (from both III.4 and III.6) showed complete retention of intron 30 (cRi30, Supplementary Fig. S13), as had been previously shown by the splicing assay using minigenes generated from genomic DNA of III.8. An anomalous second RT-PCR band of intermediate size (420 bp) was detected in both carriers. Sequence analysis of the purified 420-bp band (Supplementary Fig. S14) revealed that it actually contained two similar-sized transcripts with partial retention of the second half of intron 30, pRi30/AG1 (420 bp) and pRi30/AG2 (417 bp), with the same structure as misspliced transcripts identified by the minigene assay. Thus, *MYBPC3* expression analyses of total RNA from blood of carriers confirmed the minigene splicing results, which have anticipated that c.3331−26T>G promotes complete and partial intron 30 retention. Also, in this case, the nucleotide substitution c.3331−26T>G has been identified in the sequence of the aberrant transcripts (Supplementary Figs. S13 and S14) amplified from blood RNA of both carriers (III.4, III6).

Additionally, we performed a second RT-PCR assay targeting exons 2–3 using the same cDNA samples to analyze *MYBPC3* splicing in the presence of the c.292+177C>T variant located in intron 2, identified by massively parallel sequencing in the index patient and also present in III.7 and III1, but not in III.4 and III.6. As shown in Fig. 6b, in addition to the expected RT-PCR product of 292 bp, no other splice forms were detected in the targeted mRNA region by this second splicing assay. In the four samples, the cDNA sequence of the 292-bp RT-PCR band corresponded to normal *MYBPC3* mRNA with canonical splicing (Supplementary Fig. S15), with intron 2 removed and exons 2 and 3 normally ligated in the presence of the c.292+177C>T intronic variant. This second splicing assay pointed in the same direction as the disease cosegregation analysis in Fig. 1, indicating no association of c.292+177C>T with HCM in the family.

Both the minigene and patients’ RNA splicing assays revealed that the *MYBPC3* c.3331−26T>G variant promoted complete intron 30 retention in the mRNA, and also partial retention of the second half of intron 30 by the use of the canonical GT dinucleotide of the splice donor site and the alternative use of two consecutive AG dinucleotides in the middle of intron 30. With the aim of assessing the relative potential strength of the two preexisting cryptic splice acceptor sites in *MYBPC3* intron 30, we tested whether these cryptic sites that were used in vivo could be detected by in silico analyses. Using the complete *MYBPC3* intron 30 sequence (including the i30-E31 junction) as an input, all potential splice acceptor sites were predicted using the FSPLICE and SPLM platforms. As shown in Supplementary Table S5, both bioinformatics tools have detected three potential splice acceptor sites in intron 30, the canonical one and the two AG1 and AG2 cryptic sites. The software outputs have also shown that sites AG1 and AG2 account for about 70% and 40% of the score obtained by the canonical acceptor site, respectively. The introduction of the c.3331−26T>G variant in the input sequence resulted in identical outputs for cryptic sites and slightly reduced for the canonical site.

The c.3331−26T>G variant had escaped detection by several deep-learning-based prediction programs to identify genetic variants that cause cryptic splicing. In the search for a molecular mechanism that could explain the retention of intron 30 in the *MYBPC3* mRNA promoted by the intronic variant c.3331−26T>G, we performed an additional in silico analysis. The analysis of pre-mRNA splicing factor binding sites by SpliceAid, a database of strictly experimentally assessed target RNA sequences in humans, predicted that the nucleotide substitution T>G creates two binding sites present only in the mutated sequence (Supplementary Fig. S16). According to the SpliceAid output, binding sites for SRSF5 (splicing factor arginine/serine-rich 5, also known as SRp40) and SRSF2 (splicing factor arginine/serine-rich 2, also known as SC35) are present only in the mutated sequence: AGAGG for SRp40 (score + 5) and GGCCUCC for SC35 (score + 5); affected nucleotide is underlined in both cases. The software assigned a positive score to the target sequences that facilitate exon definition and a negative score to the target sequences that facilitate intron definition.

Collectively, the *MYBPC3* expression analyses by both minigene and blood mRNA assays, involving different cell hosts in culture and in vivo, represent strong evidence for the classification of the *MYBPC3* c.3331−26T>G nucleotide substitution as a spliceogenic variant.

### Frequency of cryptic splice-altering *MYBPC3* variants in a large cohort of HCM probands

Finally, with the aim of obtaining an overview of the frequency of known, previously characterized cryptic splice-altering *MYBPC3* variants in HCM, we examined our Health in Code (HIC) database cohort of unrelated patients with HCM, consisting of a total of 9,611 HCM probands with massively parallel sequencing data covering *MYBPC3* proximal intronic regions, of which 3,437 were sequenced including complete introns. Firstly, from published articles, we have compiled a comprehensive list of 46 (including c.3331−26T>G from this study) cryptic splice-altering single-nucleotide-substitution *MYBPC3* variants located in intronic sequences but outside the essential dinucleotides of the donor and acceptor sites (Table 1). All variants listed in Table 1 were reported to disrupt normal *MYBPC3* pre-mRNA splicing using minigene and/or blood RNA assays, which led to the identification and characterization of the resulting aberrant misspliced ​​transcripts.

We have included all splice-altering *MYBPC3* variants located in intron sequences that have been identified in the largest minigene-based splicing study^21^. Although these variants are generally interpreted as probably pathogenic, further studies would provide additional evidence. In particular, variants that promote misspliced transcripts which are predicted to introduce small in-frame deletions/insertions, for example (v11) c.906−8T>C and (v12) c.926+4A>G, or alterations at the extreme 3’ end of the gene, as in (v46) c.3628−12C>G, require further follow-up to obtain additional evidence.

All other splice-altering *MYBPC3* variants in Table 1 have been explicitly interpreted as either pathogenic or likely pathogenic. The alternative allele of these variants is extremely rare or absent in the population frequency database gnomAD. In addition to functional studies, evidence of cosegregation with the disease was reported in several cases.

Out of a total of 46 cryptic splice-altering *MYBPC3* variants listed in Table 1, 25 variants (54%) were represented in our cohort with at least one patient. These 25 variants were identified in 122 probands with HCM. Most variants (19/25) were identified in patients with HCM but not in patients with other inherited cardiovascular diseases. Six variants (6/25) were also detected in seven patients with other cardiovascular diseases, as detailed in the legend of Table 1. Therefore, in our cohort, cryptic splice-altering *MYBPC3* variants showed an association with the HCM phenotype with a specificity of 94.6% (122/129).

Nine (9/46) *MYBPC3* splice-altering variants—(v10) c.906−36G>A, (v14) c.927−9G>A, (v18) c.1224−80G>A, (v19) c.1224−52G>A, (v21) c.1224−19G>A, (v23) c.1227−13G>A, (v32) c.1927+600C>T, (v40) c.3190+5G>A and (v42) c.3330+5G>C—were recurrent and over-represented in our cohort, being detected in six to fifteen probands, accounting for most HCM patients (76%, 93/122) with this type of variants and representing strong evidence for pathogenicity, in addition to the criteria previously reported in references of Table 1. The splicing defects caused by these recurrent variants are predicted to create a downstream frameshift and premature termination codon in an NMD-sensitive region, with one exception. It was reported that the variant (v18) c.1224−80G>A promotes a misspliced transcript with the retention of the last 78 nucleotides of intron 13 in the mRNA, predicted to insert 26 amino acids in-frame^16^.

Sixteen (16/46) variants were identified in one to four probands, of which nine variants were identified in only one proband, and 21 (21/46) variants were not detected. These data from our cohort supported the emerging concept that a wide range of cryptic splice-altering *MYBPC3* variants located in introns but outside essential dinucleotides of canonical splice sites represent an increasingly recognized cause of hypertrophic cardiomyopathy^18,19^ (see also references in Table 1). In our cohort, about 80% (37/46) of known *MYBPC3* splice-altering variants are non-recurrent variants, present in a few patients (≤ 4), proband-specific or rare enough to be undetected in a large study. In these cases, the application of strong evidence of pathogenicity such as over-representation and cosegregation is prevented or limited, and variant characterization largely depends on functional RNA splicing assays^17^.

To assess whether the known cryptic splice-altering *MYBPC3* variants could have been detected or prioritized in silico if presented for the first time in the output of a diagnostic test, the SpliceAI program was run for each variant to obtain a Δ score probability of being splice-altering. The values obtained for each variant revealed that the deep-learning algorithm SpliceAI prioritized 36/46 variants (78%) as being splice-altering candidates with a high recall cutoff with higher sensitivity (Δ score ≥ 0.2), of which 25/46 (54%) variants were detected with a more restrictive cutoff point (Δ score ≥ 0.5). Taking into account that variants in Table 1 are distributed in proximal, intermediate and distal/deep intronic positions, in general, we considered that the SpliceAI sensitivity for the detection of cryptic splice-altering *MYBPC3* variants was in line with the expected one^22,30^. For cryptic splice-altering variants that produce detectable splice isoforms, SpliceAI has an overall sensitivity (Δ score ≥ 0.5) of 71% when a variant is near exons and 41% for deep intronic variants^22^. Thus, although some splice-disrupting *MYBPC3* variants occasionally escaped being detected by the SpliceAI algorithm even with a high recall score, as it was the case of the elusive *MYBPC3* c.3331−26T>G spliceogenic variant characterized in this study, this algorithm contributes, in combination with other approaches, to the first in silico step for prioritizing a number of rare *MYBPC3* variants that could be detected by massively parallel sequencing of patients’ genomic DNA.

## Discussion

In this work, the finding of a genotype-negative hypertrophic cardiomyopathy (HCM) proband in a pedigree with several affected members indicating a familial origin of the disease has driven the discovery of a causative gene variant. Focusing on highly prevalent HCM genes, we identified in the proband two rare intronic heterozygous single-nucleotide substitutions in the *MYBPC3* gene, c.292+177C>T and c.3331−26T>G. Family tests have shown that the c.292+177C>T variant, identified only in this pedigree, does not appear to be associated with the disease. In contrast, the emerging evidence of cosegregation of the *MYBPC3* c.3331−26T>G variant with the disease in two of the proband’s descendants and, importantly, in two unrelated HCM index patients, revealed by a frequency analysis in our HCM cohort, has promoted the experimental functional characterization of the rare intronic variant *MYBPC3* c.3331−26T>G.

Both the minigene and blood pre-mRNA splicing assays revealed that the single-nucleotide substitution *MYBPC3* c.3331−26T>G acts as a cryptic splice-altering variant promoting the complete and partial retention of intron 30 in different cell types and within simplified or complete gene versions, representing strong evidence for its splice-altering activity in different cellular or genetic backgrounds. Since the minigene assay allows to isolate the effect of the variant without interference with the wild-type version, it was observed that the nucleotide G at position c.3331−26 in the MUT minigene actively promotes a major disruption of pre-mRNA splicing, leading to undetectable levels of normal mRNA or protein in transfected cells, in a sharp contrast with cells transfected with the REF minigene. In both minigene and blood splicing assays and in both reference and mutant samples, using RT-PCR and Sanger sequencing, it is not possible to rule out the presence of other mRNA forms which are less abundant, unable to be co-amplified with the normal transcript, rapidly degraded or specific for the cardiac tissue. Taking all results together, we considered that the activation of preexisting cryptic splice acceptor sites, revealed by in silico analysis as potentially stronger AG1 and weaker AG2, which led to the partial retention of intron 30, is a secondary event to the complete intron 30 retention promoted by the c.3331−26T>G variant. This primary event could promote the selection of normally silenced preexisting cryptic splice sites, expanding the diversity of the missplicing consequences. The three aberrant transcripts induced by the *MYBPC3* c.3331−26T>G variant are predicted to contain a premature termination codon in an NMD-sensitive region and expected to lead to haploinsufficiency in carrier patients.

The previously reported *MYBPC3* c.1898−23A>G^18^ variant represents an example of the utility of branchpoint-specific algorithms^31^ for detecting splice variants located in the branchpoint area that escaped being detected by SpliceAI. However, the elusive variant characterized in this study, c.3331−26T>G, disrupts normal splicing without displaying significantly altered in silico scores for major regulatory splicing elements, including the branchpoint. Variant-induced alteration of regulatory splice factor binding sites may contribute to missplicing events even when other splicing elements (splice donor site, branchpoint, polypyrimidine tract and splice acceptor site) are not predicted to be significantly altered. In fact, as revealed by a database of experimentally assessed target RNA sequences that are bound by regulatory splicing proteins in humans (SpliceAid, Supplementary Fig. S16), we considered that the alteration of regulatory binding sites created by the c.3331−26T>G variant may contribute to explain the mechanism underlying the observed missplicing events leading to improper recognition and removal of intron 30.

Once we characterized the *MYBPC3* c.3331−26T>G substitution as a splice-altering variant, we considered unlikely that the c.292+177C>T variant in intron 2 would also be spliceogenic, since the index patient carried both variants in trans. Compound heterozygous or homozygous truncating pathogenic *MYBPC3* variants cause severe neonatal cardiomyopathy^32,33^, which is not the case of the index patient of this family. Furthermore, the blood RNA splicing assay revealed only normal canonical splicing of exons 2 and 3, with removal of intron 2 in the presence of c.292+177C>T. Taken together, these results suggested that the *MYBPC3* c.292+177C>T variant was not associated with the disease.

According to the American College of Medical Genetics and the Association of Molecular Pathology guidelines^27^, and recommendations by ClinGen’s Inherited Cardiomyopathy Expert Panel^28^, the *MYBPC3* c.3331−26T>G variant was classified as likely pathogenic, as per the following criteria: PS3, strong evidence based on minigene and patients’ RNA splicing assays as well as minigene protein expression analysis supporting a damaging effect on the *MYBPC3* gene product; PM2, moderate evidence based on the absence of the variant in the large genome population database gnomAD; and PP1, supporting evidence based on cosegregation with the disease in three affected family members and two additional unrelated HCM probands.

The frequency analysis extended to known *MYBPC3* spliceogenic variants has revealed that six recurrent cryptic splice-altering *MYBPC3* variants in our cohort (c.906−36G>A, c.927−9G>A, c.1224−80G>A, c.1224−52G>A, c.1224−19G>A, c.1227−13G>A) are caused by the same nucleotide substitution preceded by a minus sign, − (n)G>A, frequently detected with a high SpliceAI score. In these cases, the change occurs in the first G of two consecutive G nucleotides, GG in the reference sequence (Supplementary Fig. S17), and the nucleotide substitution changes the first G to an A, creating an AG dinucleotide acting as a cryptic splice acceptor site and usually promoting the inclusion of intronic sequences in the mRNA and extending the downstream exon. Although the substitution G>A is the most frequent, other options leading to the creation of an active AG dinucleotide are known, such as A>G (in c.1224−21A>G) and C>A (in c.1227−14C>A). These active cryptic AG dinucleotides are created within the AG-exclusion zone^34^, defined as the region between the splice acceptor site and the first upstream AG (including the branchpoint sequence and the polypyrimidine tract) that is generally devoid of AG dinucleotides. AG dinucleotides are expected to be under selective pressure against their location between the branchpoint and the splice acceptor site. Recognition of essential splicing elements involves a scanning search process for the splice acceptor site that will usually use the first AG downstream of the branchpoint for splicing of the adjacent exon^34^.

Taking into account that most AGs created by variants in the AG-exclusion zone are pathogenic, becoming active cryptic splice acceptor sites that outcompete the canonical AGs, it has been recently proposed^35^ that following simple rules such as including the search for variant-created AG dinucleotides that break the AG-exclusion zone provides an effective prioritization method of splice-disrupting candidate variants in proximal intronic regions. Splice-altering variants proximal to the donor or acceptor sites can also weaken or suppress natural splice signals without creating active competitive splice dinucleotides, and deep intronic splice variants can promote the inclusion of cryptic exons within large introns which are particularly difficult to predict in silico and to detect in experimental splicing assays.

The c.3331−26T>G variant actually creates a new AG dinucleotide which is the first upstream from the canonical acceptor site on intron 30 (Supplementary Fig. S17); however, both in silico and experimental splicing analyses suggested that it is not an active splice acceptor dinucleotide, probably because other necessary splicing elements are not compatible with the flanking sequences. At recommended thresholds, both the SpliceAI and Alamut outputs suggested that the c.3331−26T>G variant is neither involved in the loss of the natural acceptor site of intron 30 nor in the gain of a new acceptor site. One of the four independent algorithms included in the Alamut software package (GeneSplicer, Supplementary Fig. S2) generated a minority prediction suggesting that the c.3331−26T>G variant could reduce the efficiency of the natural acceptor site at c.3331 by − 11.7%, which is insufficient for prioritizing candidate splice-altering variants.

Interestingly, detailed analysis of SpliceAI outputs obtained with different windows (maximum distance of nucleotides analyzed from the c.3331−26T>G variant) have shown that the deep-learning algorithm is actually able to correctly predict, albeit with low scores, the activation of the two preexisting cryptic splice acceptor sites AG1 and AG2 (located in the *MYBPC3* pre-mRNA intron 30 upstream of c.3331−26T>G), as revealed by functional splicing assays. SpliceAI with any window selected ≥ 66 bp (and up to 10,000 bp allowed) shows that the maximum Δ score (0.16, Supplementary Table S2) is an acceptor gain at 66 bp from the variant, corresponding to the cryptic splice acceptor site used by the pRi30/AG1 transcript. Nevertheless, with a window of 65 bp, SpliceAI shows that the maximum Δ score (0.10, Supplementary Fig. S17) is an acceptor gain at 63 bp from the variant, corresponding to the cryptic splice acceptor site used by the pRi30/AG2 transcript. The SpliceAI output identifies a unique nucleotide position for each of the four predicted categories (acceptor–donor, gain–loss) with a maximum Δ score in the selected window, and if more than one acceptor gain position is predicted, it is shown only when selecting different windows.

The relative strengths assigned by SpliceAI to each preexisting cryptic splice acceptor site, stronger AG1 and weaker AG2, are consistent with those values obtained with other in silico predictors used (Supplementary Table S5). The SpliceAI output does not provide any indication that natural splice donor and acceptor sites of intron 30 could be lost or weakened; therefore, the functional consequences of the c.3331−26T>G variant, leading to a major disruption of pre-mRNA splicing by promoting complete and partial retention of the second half of intron 30, could not have been anticipated on the basis of the SpliceAI output.

However, it is tempting to speculate if the c.3331−26T>G variant and others in Table 1 could have been prioritized for functional studies using a lower SpliceAI Δ score threshold and different range of analyzed nucleotides. Recently, SpliceAI performance benchmarking studies using different datasets corresponding to single-gene and multi-gene variants have obtained different optimal SpliceAI thresholds for each dataset, but noted that they were lower than initially recommended (Δ score cutoff of 0.5), about five^30^ and up to ten^36^ times lower, and the performance may vary depending on the gene and the location of the variant.

Although we consider that lowering the SpliceAI threshold in personalized genetic diagnostics may lead to an increasing number of candidate variants with uncertain significance or lower validation rate, it could be expected that the predictive power of SpliceAI at low scores and different selected windows would be potentially relevant to some clinical cases with an elusive genetic diagnosis, which are often identified by cardiologists and geneticists, as the one described in this work.

Finally, we considered that the frequency of known cryptic splice-altering *MYBPC3* variants in our HCM cohort supports the notion that this type of variants represents an increasingly recognized cause of HCM, as they are usually expected to lead to haploinsufficiency in carrier patients, and also revealed that most cryptic splice-altering variants are non-recurrent, either present in a few patients, proband-specific or undetected in large studies. The identification of non-recurrent elusive spliceogenic *MYBPC3* variants that escaped detection by in silico algorithms represents a challenge for genetic diagnosis of HCM, and contributes to solving a fraction of HCM cases with inconclusive or negative genetic test results.

In this work, the application of tailored RNA splicing assays after a negative massively parallel sequencing standard DNA test has played an essential role to reach a personalized genetic diagnosis of HCM. Considering that spliceogenic variants may represent a widespread and prevalent cause of different genetic disorders, the integration of DNA and RNA diagnostics is expected to be also relevant to other less common inherited cardiovascular diseases, providing clinical benefits for disease management in patients and their families.

## Methods

### Patient consent for research and publication

Written informed consent was obtained from all family participants for the genetic analysis and functional characterization of the *MYBPC3* c.3331−26T>G variant and for the publication of anonymized data obtained through the clinical characterization and the scientific research carried out. Ethical approval has been obtained from the Research Ethics Committee of Badajoz (Spain). In addition, an Ethical approval has been obtained from the Clinical Research Ethics Committee of Galicia (Spain) for evaluation of the utility of genetic diagnosis in the prognostic assessment of patients with hypertrophic cardiomyopathy. All probands gave written informed consent for genetic testing. All methods were carried out in accordance with relevant guidelines and regulations. Electrophoretic gels, films and blots in figures are presented in compliance with the digital image and integrity policies of the Nature Research journals. Full-size gel and blot images are presented in the Supplementary information file S1.

### Clinical characterization

Clinical diagnosis of hypertrophic cardiomyopathy (HCM) in the index patient was established by imaging with echocardiography according to current clinical practice guidelines^9,10^. Twelve-lead electrocardiogram and continuous 24 h-Holter monitoring were also performed. The finding of a severe phenotype in the index patient prompted the clinical evaluation of the next-generation family members. The index patient’s descendants were clinically evaluated under standard protocols and cardiac magnetic resonance was also performed in some cases when possible. Criteria for HCM of index patients’ descendants allow a more limited left ventricular wall thickness^9,10^. Family history and available medical records were reviewed and relevant clinical events were collected and annotated in the family pedigree.

### Genomic DNA purification

Genomic DNA from peripheral blood of the index patient and relatives was purified on the QIAsymphony SP robot using the QIAsymphony DNA Midi Kit (Qiagen). Eluted genomic DNA was quantified using the Nanodrop 1000 Spectrophotometer (Thermo Fisher Scientific) and DNA integrity was assessed on the automated electrophoresis unit TapeStation 2200 (Agilent Technologies).

**Massively parallel sequencing** Targeted massively parallel sequencing of the index patient’s genomic DNA was performed with an Agilent SureSelect custom library covering 251 genes that constitute our current cardiomyopathy panel. This panel is detailed in Supplementary Table S1 and includes the following gene categories: (1) sarcomeric genes with definitive clinical evidence that they cause HCM: the eight core sarcomeric genes (*MYBPC3*, *MYH7*, *ACTC1*, *MYL2*, *MYL3*, *TNNT2*, *TNNI3* and *TPM1*) that account for more than 99%^4^ of pathogenic or likely pathogenic HCM variants*,* and the *TNNC1* gene, with moderate evidence^6,37,38^; (2) non-sarcomeric genes^4,6,38^ with definitive clinical, strong or moderate evidence: *ACTN2*, *PLN*, *CACNA1C*, *CSRP3*, *DES*, *FLNC*, *FHL1*, *FHOD3*, *JHP2*, *RIT1* and *ALPK3*; (3) HCM syndromic genes^6,37–39^: *LAMP2*, *PRKAG2*, *GLA*, *TTR*, *PTPN11*, *RAF1*, *DTNA*, *TAFAZZIN*, *FXN*, *SOS1* and *AGL*; (4) genes relevant to other cardiomyopathies, cardiac arrhythmias and sudden death; and (5) genes with weak clinical evidence and for research purposes only.

The Agilent's custom-designed probes for the cardiomyopathy panel cover all coding exons plus 50 flanking intronic nucleotides, with a particular design for the *MYBPC3* gene: all exons including the complete sequence of introns; and for the *PKP2*, *FLNC*, *DSP* and *LMNA* genes: coding exons plus 100 flanking intronic nucleotides. DNA libraries were prepared using the SureSelect XT Target Enrichment Kit (Agilent Technologies) following the manufacturer's instructions. Each DNA sample (3 µg) was fragmented by sonication (Covaris E220). After repairing the DNA fragments and adding an adenine base to the 3' ends to ligate the sequencing adapters, the adaptor-ligated fragments were amplified by PCR. In all these steps, the samples were purified with AMPure XP beads. Hybridization between DNA libraries and the SureSelect custom probes was performed in a Verity thermocycler (Applied Biosystems) for 16 h and the targeted regions were captured on streptavidin beads. For sample identification, each captured DNA library was amplified with an appropriate index tag by PCR. After pooling libraries for multiplexed sequencing, cluster amplification (using the Illumina Paired-End Cluster Generation Kit) and sequencing were performed on the NovaSeq 6000 Sequencing System platform (Illumina).

Bioinformatics analysis was carried out with a custom pipeline including software for variant calling and annotation according to the human reference genome version GRCh37/hg19. Massively parallel sequencing data were collected and analyzed using a proprietary bioinformatic pipeline (NDNA-Pipeline, v.01) that includes both sample demultiplexing (using Illumina software bcl2fastq) and all the required steps to obtain an annotated variant report, along with the corresponding coverage and quality parameters. NDNA-Pipeline consists of three logical stages executed sequentially. (1) Alignment, alignment refinement and adjustment. Alignment was performed with the software package Burrows-Wheeler Aligner, BWA on paired-end reads and raw alignment was improved by the Genome Analysis Toolkit, GATK. (2) Variant calling, variant normalization and quality scoring. Several open source genotypers (samtools + bcftools, haplotypeCaller, UnifiedGenotyperCaller, varDict) were executed at this stage, and finally a consensus step was performed to obtain a unified calling quality. (3) Annotation. This step is performed with a custom DB-annotator utility that was created for annotation of variant-associated data at genetic, molecular, in silico, clinical and population level from internal and external databases. The Integrative Genomics Viewer (IGV v2.9) was used for visualization of aligned massively parallel sequencing reads.

Filtering of variants was performed using the NCBI database of single-nucleotide polymorphisms (www.ncbi.nlm.nih.gov/SNP), Genome Aggregation Database (gnomad.broadinstitute.org) and the in-house proprietary database of genotypes of about 20,000 consecutive unrelated index cases with different inherited cardiovascular diseases. Health in Code’s proprietary database (HiC-Mutaciones v.10.0) integrates all data from the annotation stage of the NDNA-Pipeline and prioritizes variants in clinically relevant genes according to population frequency, variant location and predicted consequences, pathogenicity, and clinical and cosegregation data.

### In silico analysis of variant‐induced alterations of pre-mRNA splicing

The probability of a variant being splice-altering was estimated in silico using the deep learning-based tool SpliceAI^22^ with a window of ± 200 bp (maximum distance of analyzed nucleotides from the variant) unless otherwise indicated, and the splicing prediction module of the software package Alamut Visual (v.2.11.0, Interactive Biosoftware). The output of in silico predictive algorithms was interpreted following the guidelines and recommendations from software developers, which are indicated in the corresponding section of the results. For SpliceAI, the following cutoffs apply: 0.2 (high recall), 0.5 (recommended) and 0.8 (high precision). For Alamut, a consensus prediction of at least three of four algorithms is recommended. Multiple lines of computational evidence are needed to contribute to variant classification^27^. The *MYBPC3* c.3331−26T>G variant, located within the branchpoint area, was evaluated with the following branchpoint-specific algorithms: Branchpointer^40^, Branch Point Selection on RNA (RNABP^41^), LaBranchoR^42^ and Branch Point Prediction (BPP^43^). All potential splice acceptor sites in *MYBPC3* intron 30 were predicted using two matrix-based splice site programs: FSPLICE 1.0 and SPLM platforms^44^. The analysis of splicing factor binding sites was performed with SpliceAid, a database of strictly experimentally assessed target RNA sequences in humans^45^. Each software was downloaded or used from the websites detailed in the Code Availability section.

### Family genetic testing

Sanger DNA sequencing was used for independent confirmation of massively parallel sequencing variants, family testing and phase determination of two *MYBPC3* variants identified in the index patient, located in intron 2 (NM_000256.3:c.292+177C>T) and intron 30 (NM_000256.3:c.3331−26T>G). The forward and reverse primers used to amplify intron 2 were INT2-FW (5'-CCA GCG ACT CTC CAT CCA TC-3') and INT2-RV (5'-CGT CTG TGC AGG GTC ATC TC-3') and the primer pair for amplification of the region between exons 30 and 31 were EX30-31-FW (5'-CTC TGC TGA TCT GAA TCC CTC CAT-3') and EX30-31-RV (5'-GGT GGA GAG AAA GCA GGG GAG A-3'). As previously described^46^, PCR was performed with KAPA2G Fast HotStart ReadyMix (Roche) and the PCR products were subjected to an enzymatic clean-up using exonuclease I and thermosensitive alkaline phosphatase (Thermo Scientific) following manufacturer's protocols. Bidirectional sequencing reactions were performed using the Big Dye Terminator v3.1 cycle sequencing kit (Applied Biosystems) with the same primers used in PCR, except for the reverse sequencing primer of the EX30-31 amplicon (EX30-31-RV2: 5'-CTG GAC CAG CGC CTA AAG TT-3'). The cycle sequencing products were purified with the BigDye XTerminator purification kit (Applied Biosystems) and subjected to capillary electrophoresis with the ABI3730 DNA Analyzer (Applied Biosystems)^46^. Sanger electropherograms were analyzed by Variant Reporter v1.0 (Applied Biosystems) and the ABI files (.ab1) were visualized with the Chromas software.

### *MYBPC3* minigene construction

The strategy for minigene construction and analysis was similar to that previously described^46^. *MYBPC3* minigenes were designed with a three-exon (E30-E31-E32) and two-intron (i30-i31) structure that included the variant of this study, c.3331−26T>G, located in intron 30. The corresponding fragment of 767 bp was generated by high-fidelity PCR (Phusion Hot Start II high-fidelity DNA polymerase) of genomic DNA isolated from the patient codified as III.8 in the pedigree (Fig. 1), heterozygous carrier of the *MYBPC3* c.3331−26T>G variant. The cloning primers used were forward NotI-462-E30 (5'-G TCT GCG GCC GCC AAG CCA AGT CCT CCC CAG GAT-3') and reverse EcoRV-537-E32 (5'-CAG CGA TAT CAG CTT GGG GCT ACC CCG GAC AG-3') and each included the indicated restriction enzyme recognition sites.

The PCR product of the expected size was subjected to double NotI/EcoRV restriction digestion, purified by agarose gel electrophoresis and eluted from gel slices. The purified insert was directionally cloned in-frame into the NotI and EcoRV sites of the p3XFLAG-Myc-CMV26 vector (Sigma-Aldrich) for dual-tagged expression of N-terminal 3XFLAG and C-terminal Myc using standard molecular cloning techniques, as it was described in a previous work^46^. The first nucleotide of E30 was omitted from the forward primer design to allow cloning the first complete codon of E30 (AAG, underlined in the forward primer) in-frame with the N-terminal 3XFLAG epitope. The second nucleotide of E30 (C, also underlined in the forward primer) was preserved since it completed the reading frame coming from the 3XFLAG vector sequence after ligation. The normal reading frame is indicated with spaces in the cloning primer sequences. The reverse cloning primer included the last nucleotides of E32, which ends with a complete codon (AAG, underlined in the reverse primer as CTT).

Plasmid DNA was isolated from transformed and ampicillin-resistant XL1-Blue Supercompetent *E. coli* cells (Stratagene), using the QIAprep Spin Miniprep Kit (Qiagen) and separated by agarose gel electrophoresis along with the empty vector control lane to identify positive constructs containing the insert. Positive *MYBPC3* minigene constructs were subjected to full-length insert Sanger DNA sequencing, with the following sequencing primers located in the vector sequences: forward 480 (5'-GCA GAG CTC GTT TAG TGA ACC GTC-3') and reverse 481 (5'-GCA ACT TCC AGG GCC AGG AG-3'). Finally, we have generated and characterized the reference 1446 and mutant 1447 *MYBPC3* minigenes, carrying the reference T or alternative G nucleotide, respectively, corresponding to the *MYBPC3* c.3331−26T>G variant. The full-insert DNA sequencing chromatograms of the reference and mutant plasmids generated with the forward primer 480 are shown in Supplementary Figs. S5 and S6, respectively. Sanger chromatograms also showed that insert ends were cloned in-frame with both N- and C-terminal tags. This cloning strategy allowed subsequent minigene expression analysis at the mRNA and protein levels, after cellular transfection.

### Cell culture for minigene splicing assay

The consequences of the *MYBPC3* variant c.3331−26T>G on pre-mRNA splicing were evaluated, as previously described^46^, by RT-PCR and Western blot analysis of HeLa cells transiently transfected with the reference 1446 and mutant 1447 *MYBPC3* plasmids. The HeLa cell line was purchased from the European Collection of Authenticated Cell Cultures (Sigma-Aldrich) and maintained in the cell culture medium recommended by the manufacturer. Primary stocks propagated from the original frozen culture were used (passage <  + 8). The day before transfection, cells were trypsinized from culture flasks and plated in 12-well culture plates, allowed to attach overnight and transiently transfected with 1.0 µg of plasmid DNA. Cellular transfections with each plasmid were performed in triplicate wells using Lipofectamine 3000 (Invitrogen) following the manufacturer’s instructions. Transfection experiments were repeated twice and cells were harvested 48 h after transfection, processing each cell culture well independently for RNA or protein extraction.

### *MYBPC3* minigene expression analysis at the mRNA level

For RT-PCR analysis, total RNA from transfected cells was purified with the RNeasy-Mini Kit (Qiagen) according to the manufacturer’s protocol and evaluated by an automated electrophoresis system (Agilent 2200 TapeStation). All samples showed high quality RNA integrity numbers (RIN>9). First-strand cDNA synthesis reaction was performed with one µg of total RNA, SuperScript IV (Invitrogen) and oligo-dT primers following the manufacturer's recommendations and then subjected to RT-PCR with KAPA2G Fast Hot Start Ready Mix (KAPA Biosystems) and primers located in different minigene exons. The forward primer 529 (5'-GAT CAT GAC ATC GAT TAC AAG GAT GAC G-3´) was located in the N-terminal vector sequence and the reverse primer 542 (5'-GCT CAT AGG TGA TGC CTG GTC TGG-3') in the junction between exons 31 and 32, designed for the amplification of a minigene-derived *MYBPC3* cDNA product of 361 bp. The amplification of endogenous *RPL19* cDNA (537 bp) was performed with forward primer 590 (5'-CGG CCG CAG CCA TGA GTA T-3') and reverse primer 588 (5'-GCT CTT CAC GGC GCT TGC G-3') and used as a control for the synthesis of cDNA. A standard thermocycler program with 25 cycles was used (95 °C-3 min, 95 °C-15 s, 60 °C-15 s, 72 °C-15 s and 72 °C 1 min). Negative controls, including non-transfected cells, non-reversed transcribed RNA (RT −) and non-template were used. RT-PCR products were separated by electrophoresis in 2% agarose gels, visualized by ethidium bromide staining, photographed using VersaDoc 1000 (Bio-Rad), sliced from gel, purified (QIAquick gel extraction kit, Qiagen) and sequenced with the same primers used in the PCR.

### *MYBPC3* minigene expression analysis at the protein level

Western blot analysis was performed as described in^46^. Cell samples from each well plate were independently homogenized and solubilized in 100 µl of Tris–Glycine SDS sample buffer (Novex Life Technologies) supplemented with complete protease inhibitor cocktail (Roche). Extracted proteins were separated by SDS-PAGE, blotted onto PVDF membranes (Hybond-P, GE Healthcare) and probed with mouse monoclonal anti-FLAG M2 (F3165, Sigma-Aldrich, 1:20,000) or anti-Myc tag (SAB2702192, Sigma-Aldrich, 1:900) antibodies, followed by incubation with anti-mouse IgG peroxidase antibody (A9917, Sigma-Aldrich, 1:25,000). Immunological signals were detected by Super-Signal West Pico PLUS chemiluminescent substrate (Thermo Scientific). Protein extracts from non-transfected cells were used as negative controls. The SeeBlue Plus2 pre‐stained protein standard (Invitrogen) was included in each membrane. Equivalence of protein loading was confirmed by Amido Black 10B staining of blots after immunodetection. All gels and blots presented in figures contained a set of samples processed and analyzed in parallel.

### RT‐PCR analysis of *MYBPC3* expression in patients’ blood

We have studied the functional splicing consequences of the *MYBPC3* c.3331−26T>G variant located in intron 30 by analysis of ectopic expression of *MYBPC3* in blood samples from several family members, two from carriers (III.4 and III.6) and another two from non-carriers (III.1 and III.7) to be used as controls. The four of them were a son and three daughters of the index patient. The same samples were also used to assess splicing in the presence of the *MYBPC3* c.292+177C>T variant in intron 2, which was present in III.1 and III.7 but not in III.4 and III.6. Whole blood was collected in PAXgene Blood RNA tubes (PreAnalytix) for stabilization and transport to Health in Code’s laboratory, and stored at − 80 °C until analysis. Total intracellular RNA was isolated and purified from stabilized whole blood using the PAXgene Blood RNA kit (PreAnalytix) following the manufacturer’s protocol, which included on-column DNase digestion. The four samples were processed and analyzed in parallel. The quality and quantity of total RNA were evaluated by an automated electrophoresis system (Agilent TapeStation). In the four samples, the ratio of the 28S/18S ribosomal RNA bands was between 1.9 and 2.4, with an estimated RNA integrity number (RIN) between 8.0 and 8.5, as calculated by the TapeStation algorithm, representing high-quality RNA with minimal degradation (Supplementary Fig. S11).

A two-step RT-PCR was used to detect *MYBPC3* expression. First-strand cDNA synthesis was performed using SuperScript IV reverse transcriptase (Invitrogen), 500 ng of total RNA and oligo(dT) primer, following the manufacturer’s protocol. PCR amplification of *MYBPC3* cDNA was performed with KAPA2G Fast HotStart DNA Polymerase (KAPA Biosystems) and primers located at the junction of different exons, the forward primer spanning the junction between exons 29–30 (543: 5'-CTG CAG GTT GTT GAC AAG CCA AGT C-3') and the reverse primer the junction between exons 31–32 (542: 5'-GCT CAT AGG TGA TGC CTG GTC TGG-3'), designed to amplify an RT-PCR product of 328 bp. A touchdown thermocycler program was used with the following two phases, first phase: 13 cycles with decreasing (Δ, 1 °C/cycle) annealing temperature (95 °C-3 min, 95 °C-30 s, Δ72 °C-30 s, 72 °C-30 s); and second phase: 23 cycles with stable annealing temperature (95 °C-15 s, 60 °C-15 s, 72 °C-15 s and 72 °C-3 min). Successful synthesis of cDNA was verified by amplification of the *RPL19* transcript (537 bp) with the same primer pair used in the minigene assay and a standard thermocycler program with 28 cycles.

The RT-PCR assay targeting the *MYBPC3* mRNA region between exons 2–3 was performed by two-round PCR using the primer pair 601 (forward, exon 2, 5'-GTG ACA TCA GCG CCA GCA ACA A-3') and 602 (reverse, junction exons 4–5, 5'-TGA TGC TGC CAC CCA CGG TC-3') in the first PCR, and the primer pair 601 and 603 (reverse, exon 4, 5'-CCA TTG AGA GCT GCT GAG CTT GA-3') in the second PCR. Products from the first PCR (touchdown thermocycler program) were diluted 1:100 and used as template for the second PCR (standard thermocycler program, 22 cycles). The expected normal length of the final RT-PCR product is 292 bp. Negative controls, including non-reverse transcribed RNA (RT −) and non-template, were used. RT-PCR products were separated by electrophoresis in 2% agarose gels, visualized by ethidium bromide staining, photographed using Versadoc 1000 (Bio-Rad), sliced from gel, purified and sequenced. Sanger sequencing of purified RT-PCR products was performed with the same primers used in PCR, BigDye Terminator v3.1 Cycle Sequencing Kit (Applied Biosystems) and the ABI3730 DNA Analyzer (Applied Biosystems). Sanger DNA chromatograms were visualized and analyzed with the Chromas software.

### Frequency of cryptic splice-altering *MYBPC3* variants in a large cohort of HCM probands

Published articles were searched and reviewed to generate a comprehensive list of known spliceogenic *MYBPC3* variants located in intron sequences but outside the essential splice donor and acceptor dinucleotides at positions + 1/ + 2 and − 1/ − 2, respectively. Splice-altering variants located in exonic sequences were not included. Criteria for inclusion required experimental confirmation with either patients’ RNA and/or minigene assays that the nucleotide substitution alters normal *MYBPC3* pre-mRNA splicing and leads to detectable misspliced transcripts. The Health in Code (HIC) database was searched for index patients carrying any of the known *MYBPC3* splice-altering variants and the frequency of each variant related to the total number of sequenced patients with hypertrophic cardiomyopathy was annotated. In calculating the frequency of each variant, only index patients from unrelated families were considered, even if a family screening was performed and additional carriers were identified in the same family. The HIC dataset included the genotype of about 20,000 consecutive and unrelated index patients with different cardiovascular diseases referred to our center for massively parallel sequencing genetic diagnosis. Nearly half of these patients correspond to HCM cases. The *MYBPC3* gene, included in the diagnostic panel, was sequenced in all cases. For *MYBPC3* variants located at deep intronic positions, only patients sequenced with our current gene panel in use since March 2019, which covers the complete *MYBPC3* gene, were included for frequency calculation. Our previous panel version, in use from March 2008 to February 2019, had included probes for covering all *MYBPC3* exons plus 50 flanking intronic nucleotides. Targeted massively parallel sequencing was performed essentially as described in a previous Methods subsection.

## Supplementary Information

Supplementary Information.

## Data Availability

The main data supporting the findings of this study are available within the article and its Supplementary information S1. Most materials and reagents used in this study are commercially available. Additional data and/or materials are available upon reasonable request, with some restrictions to protect research participants’ privacy. The *MYBPC3* c.3331−26T>G variant was submitted to the ClinVar database with the accession code SCV002011859.

## Code availability

No custom codes were generated in this work. Proprietary bioinformatics pipeline (NDNA-Pipeline, v.01) and database (HiC-Mutaciones v.10.0) were used for massively parallel sequencing data collection, analysis, annotation, and filtering of genetic variants. Alamut Visual (v.2.11.0) by Interactive Biosoftware is commercially available. The following open source codes were used for in silico prediction of RNA splicing: SpliceAI v.1.3.1, https://github.com/Illumina/SpliceAI; BranchPointer, https://www.bioconductor.org/packages/release/bioc/html/branchpointer.html; BPP, https://github.com/zhqingit/BPP; LaBranchoR, https://github.com/jpaggi/labranchor; SPLM and FSPLICE 1.0, http://www.softberry.com/berry.phtml?topic=fdp.htm&no_menu=on. Two web-based applications were used: SpliceAid (http://www.introni.it/splicing.html) and RNABP (http://nsclbio.jbnu.ac.kr/tools/RNABP/).
