# Supplementary material for: Identification of an elusive spliceogenic MYBPC3 variant in an otherwise genotype-negative hypertrophic cardiomyopathy pedigree
Source: Sci Rep. 2022 May 4;12:7284. doi: 10.1038/s41598-022-11159-y (PMC9068804; doi:10.1038/s41598-022-11159-y)
Supplement: Supplementary file 1 — Supplementary Information. [file 41598_2022_11159_MOESM1_ESM.pdf]

Supplementary Information

Identification of an elusive spliceogenic *MYBPC3* variant in an otherwise genotype-negative hypertrophic cardiomyopathy pedigree

Mario Torrado[1,2,*], Emilia Maneiro[2,3,*], Arsonval Lamounier Junior[1,2,3], Miguel Fernández-Burriel[4], Sara Sánchez Giralt[5], Ana Martínez-Carapeto[5], Laura Cazón[3], Elisa Santiago[3], Juan Pablo Ochoa[2,3], William J. McKenna[1,2,6], Luis Santomé[3] and Lorenzo Monserrat[2,3]

[1]Cardiovascular Research Group, University of A Coruña, A Coruña, Spain;
[2]Biomedical Research Institute of A Coruña, A Coruña, Spain;
[3]Cardiovascular Genetics, Health in Code, A Coruña, Spain;
[4]Genetics Unit, Hospital de Mérida, Badajoz, Spain;
[5]Cardiology Department, Hospital de Mérida, Badajoz, Spain;
[6]Institute of Cardiovascular Science, University College London, London, United Kingdom.

Supplementary Table 1. Cardiomyopathy gene panel.

A2ML1	alpha-2-macroglobulin like 1	GJA1	gap junction protein alpha 1	PERP	p53 apoptosis effector related to PMP22
AARS2	alanyl-tRNA synthetase 2, mitochondrial	GJA5	gap junction protein alpha 5	PHKA1	phosphorylase kinase regulatory subunit alpha 1
ABCC9	ATP binding cassette subfamily C member 9	GLA	galactosidase alpha	PITX2	paired like homeodomain 2
ACAD9	acyl-CoA dehydrogenase family member 9	GLB1	galactosidase beta 1	PKD2	polycystin 2, transient receptor potential cation channel
ACADVL	acyl-CoA dehydrogenase very long chain	GNB2	G protein subunit beta 2	PKP2	plakophilin 2
ACTA1	actin alpha 1, skeletal muscle	GNPTAB	N-acetylglucosamine-1-phosphate transferase subunits alpha and beta	PKP4	plakophilin 4
ACTC1	actin alpha cardiac muscle 1	GPD1L	glycerol-3-phosphate dehydrogenase 1 like	PLN	phospholamban
ACTN2	actinin alpha 2	GREM2	gremlin 2, DAN family BMP antagonist	PMM2	phosphomannomutase 2
AGK	acylglycerol kinase	GSK3B	glycogen synthase kinase 3 beta	PPA2	inorganic pyrophosphatase 2
AGL	amylo-alpha-1, 6-glucosidase, 4-alpha-glucanotransferase	GUSB	glucuronidase beta	PPCS	phosphopantothenoylcysteine synthetase
AGPAT2	1-acylglycerol-3-phosphate O-acyltransferase 2	GYG1	glycogenin 1	PPP1CB	protein phosphatase 1 catalytic subunit beta
AKAP9	A-kinase anchoring protein 9	HCN4	hyperpolarization activated cyclic nucleotide gated potassium channel 4	PPP1R13L	protein phosphatase 1 regulatory subunit 13 like
AKT1	AKT serine/threonine kinase 1	HFE	homeostatic iron regulator	PRDM16	PR/SET domain 16
ALMS1	ALMS1 centrosome and basal body associated protein	HRAS	HRas proto-oncogene, GTPase	PRKAG2	protein kinase AMP-activated non-catalytic subunit gamma 2
ALPK3	alpha kinase 3	IDH2	isocitrate dehydrogenase (NADP(+)) 2	PSEN1	presenilin 1
ANK2	ankyrin 2	ILK	integrin linked kinase	PSEN2	presenilin 2
ANK3	ankyrin 3	IRX3	iroquois homeobox 3	PTPN11	protein tyrosine phosphatase non-receptor type 11
ANKRD1	ankyrin repeat domain 1	ISM2	isthmin 2	QRSL1	glutaminyl-tRNA amidotransferase subunit QRSL1
ANO5	anoctamin 5	JARID2	jumonji and AT-rich interaction domain containing 2	RAF1	Raf-1 proto-oncogene, serine/threonine kinase
ATP5F1E	ATP synthase F1 subunit epsilon	JPH2	junctophilin 2	RANGRF	RAN guanine nucleotide release factor
ATPAF2	ATP synthase mitochondrial F1 complex assembly factor 2	JUP	junction plakoglobin	RASA1	RAS p21 protein activator 1
BAG3	BAG cochaperone 3	KAT6B	lysine acetyltransferase 6B	RASA2	RAS p21 protein activator 2
BRAF	B-Raf proto-oncogene, serine/threonine kinase	KCNA5	potassium voltage-gated channel subfamily A member 5	RBM20	RNA binding motif protein 20
BSCL2	BSCL2 lipid droplet biogenesis associated, seipin	KCND2	potassium voltage-gated channel subfamily D member 2	RBM24	RNA binding motif protein 24
C10orf71	chromosome 10 open reading frame 71	KCND3	potassium voltage-gated channel subfamily D member 3	RIT1	Ras like without CAAX 1
CACNA1C	calcium voltage-gated channel subunit alpha1 C	KCNE1	potassium voltage-gated channel subfamily E regulatory subunit 1	RRAS	RAS related
CACNA1D	calcium voltage-gated channel subunit alpha1 D	KCNE2	potassium voltage-gated channel subfamily E regulatory subunit 2	RYR2	ryanodine receptor 2
CACNA2D1	calcium voltage-gated channel auxiliary subunit alpha2delta 1	KCNE3	potassium voltage-gated channel subfamily E regulatory subunit 3	SCN10A	sodium voltage-gated channel alpha subunit 10
CACNB2	calcium voltage-gated channel auxiliary subunit beta 2	KCNE5	potassium voltage-gated channel subfamily E regulatory subunit 5	SCN1B	sodium voltage-gated channel beta subunit 1
CALM1	calmodulin 1	KCNH2	potassium voltage-gated channel subfamily H member 2	SCN2B	sodium voltage-gated channel beta subunit 2
CALM2	calmodulin 2	KCNJ2	potassium inwardly rectifying channel subfamily J member 2	SCN3B	sodium voltage-gated channel beta subunit 3
CALM3	calmodulin 3	KCNJ5	potassium inwardly rectifying channel subfamily J member 5	SCN4B	sodium voltage-gated channel beta subunit 4
CALR	calreticulin	KCNJ8	potassium inwardly rectifying channel subfamily J member 8	SCN5A	sodium voltage-gated channel alpha subunit 5
CALR3	calreticulin 3	KCNK17	potassium two pore domain channel subfamily K member 17	SCO2	synthesis of cytochrome C oxidase 2
CASQ2	calsequestrin 2	KCNQ1	potassium voltage-gated channel subfamily Q member 1	SDHA	succinate dehydrogenase complex flavoprotein subunit A
CASZ1	castor zinc finger 1	KLF10	Kruppel like factor 10	SGCA	sarcoglycan alpha
CAV3	caveolin 3	KLHL24	kelch like family member 24	SGCB	sarcoglycan beta
CAVIN1	caveolae associated protein 1	KRAS	KRAS proto-oncogene, GTPase	SGCD	sarcoglycan delta
CAVIN4	caveolae associated protein 4	LAMA2	laminin subunit alpha 2	SGCG	sarcoglycan gamma
CBL	Cbl proto-oncogene	LAMA4	laminin subunit alpha 4	SHOC2	SHOC2 leucine rich repeat scaffold protein
CCN4	cellular communication network factor 4	LAMP2	lysosomal associated membrane protein 2	SLC22A5	solute carrier family 22 member 5
CDH2	cadherin 2	LDB3	LIM domain binding 3	SLC25A3	solute carrier family 25 member 3
CHRM2	cholinergic receptor muscarinic 2	LDLR	low density lipoprotein receptor	SLC25A4	solute carrier family 25 member 4
COA5	cytochrome c oxidase assembly factor 5	LIAS	lipoic acid synthetase	SLMAP	sarcolemma associated protein
COA6	cytochrome c oxidase assembly factor 6	LMNA	lamin A/C	SNTA1	syntrophin alpha 1
COL7A1	collagen type VII alpha 1 chain	LMOD2	leiomodin 2	SOS1	SOS Ras/Rac guanine nucleotide exchange factor 1
COQ2	coenzyme Q2, polyprenyltransferase	LZTR1	leucine zipper like transcription regulator 1	SOS2	SOS Ras/Rho guanine nucleotide exchange factor 2
COX15	cytochrome c oxidase assembly homolog COX15	MAP2K1	mitogen-activated protein kinase kinase 1	SPEG	striated muscle enriched protein kinase
COX6B1	cytochrome c oxidase subunit 6B1	MAP2K2	mitogen-activated protein kinase kinase 2	SPRED1	sprouty related EVH1 domain containing 1
CRYAB	crystallin alpha B	MAP3K8	mitogen-activated protein kinase kinase kinase 8	SPRY1	sprouty RTK signaling antagonist 1
CSRP3	cysteine and glycine rich protein 3	MEF2C	myocyte enhancer factor 2C	SURF1	SURF1 cytochrome c oxidase assembly factor
CTNNA1	catenin alpha 1	MIB1	MIB E3 ubiquitin protein ligase 1	SYNE1	spectrin repeat containing nuclear envelope protein 1
CTNNA3	catenin alpha 3	MLYCD	malonyl-CoA decarboxylase	SYNE2	spectrin repeat containing nuclear envelope protein 2
CTNNB1	catenin beta 1	MRPL3	mitochondrial ribosomal protein L3	SYNGAP1	synaptic Ras GTPase activating protein 1
DES	desmin	MRPL44	mitochondrial ribosomal protein L44	TAFAZZIN	tafazzin, phospholipid-lysophospholipid transacylase
DLD	dihydrolipoamide dehydrogenase	MRPS22	mitochondrial ribosomal protein S22	TBX20	T-box transcription factor 20
DMD	dystrophin	MTO1	mitochondrial tRNA translation optimization 1	TBX5	T-box transcription factor 5
DNAJC19	DnaJ heat shock protein family (Hsp40) member C19	MYBPC3	myosin binding protein C3	TCAP	titin-cap
DNM1L	dynamin 1 like	MYBPHL	myosin binding protein H like	TECRL	trans-2,3-enoyl-CoA reductase like
DOLK	dolichol kinase	MYH6	myosin heavy chain 6	TGFB3	transforming growth factor beta 3
DSC2	desmocollin 2	MYH7	myosin heavy chain 7	TMEM175	transmembrane protein 175
DSG2	desmoglein 2	MYL2	myosin light chain 2	TMEM43	transmembrane protein 43
DSP	desmoplakin	MYL3	myosin light chain 3	TMEM70	transmembrane protein 70
DTNA	dystrobrevin alpha	MYLK2	myosin light chain kinase 2	TMOD1	tropomodulin 1
ELAC2	elaC ribonuclease Z 2	MYOM1	myomesin 1	TNNC1	troponin C1, slow skeletal and cardiac type
EMD	emerin	MYOT	myotilin	TNNI3	troponin I3, cardiac type
EYA4	EYA transcriptional coactivator and phosphatase 4	MYOZ2	myozenin 2	TNNI3K	TNNI3 interacting kinase
FAH	fumarylacetoacetate hydrolase	MYPN	myopalladin	TNNT2	troponin T2, cardiac type
FBXO32	F-box protein 32	NEBL	nebulette	TOR1AIP1	torsin 1A interacting protein 1
FGF12	fibroblast growth factor 12	NEXN	nexilin F-actin binding protein	TPM1	tropomyosin 1
FHL1	four and a half LIM domains 1	NF1	neurofibromin 1	TRDN	triadin
FHL2	four and a half LIM domains 2	NKX2-5	NK2 homeobox 5	TRIM54	tripartite motif containing 54
FHOD3	formin homology 2 domain containing 3	NKX2-6	NK2 homeobox 6	TRIM63	tripartite motif containing 63
FKRP	fukutin related protein	NNT	nicotinamide nucleotide transhydrogenase	TRPM4	transient receptor potential cation channel subfamily M member 4
FKTN	fukutin	NONO	non-POU domain containing octamer binding	TSFM	Ts translation elongation factor, mitochondrial
FLNC	filamin C	NOS1AP	nitric oxide synthase 1 adaptor protein	TTN	titin
FOXRED1	FAD dependent oxidoreductase domain containing 1	NOTCH1	notch receptor 1	TTR	transthyretin
FXN	frataxin	NPPA	natriuretic peptide A	TXNRD2	thioredoxin reductase 2
GAA	alpha glucosidase	NRAP	nebulin related anchoring protein	VCL	vinculin
GATA4	GATA binding protein 4	NRAS	NRAS proto-oncogene, GTPase	WT1	WT1 transcription factor
GATA5	GATA binding protein 5	OBSCN	obscurin, cytoskeletal calmodulin and titin-interacting RhoGEF	XK	X-linked Kx blood group
GATA6	GATA binding protein 6	OPA3	outer mitochondrial membrane lipid metabolism regulator OPA3	ZBTB17	zinc finger and BTB domain containing 17
GATAD1	GATA zinc finger domain containing 1	PDHA1	pyruvate dehydrogenase E1 subunit alpha 1	ZFHX3	zinc finger homeobox 3
GFM1	G elongation factor mitochondrial 1	PDLIM3	PDZ and LIM domain 3		

HGNC approved gene symbols and names of 251 genes included in the cardiomyopathy gene panel are indicated. Thirty-one clinical relevant genes for hypertrophic cardiomyopathy (HCM) are highlighted in colors. The eight core sarcomeric genes and *TNNC1* are highlighted in red, eleven non-sarcomeric genes in yellow and eleven HCM syndromic genes in blue.

Supplementary Table 2. SpliceAI prediction results of two rare intronic *MYBPC3* variants identified in the index patient.

MYBPC3 variant					Delta scores				Delta positions			
cDNA	#CHROM	POS	REF	ALT	DS_AG	DS_AL	DS_DG	DS_DL	DP_AG	DP_AL	DP_DG	DP_DL
c.292+177C>T	11	47372613	G	A	0.00	0.01	0.01	0.00	43	-82	-34	181
c.3331−26T>G	11	47354550	A	C	0.16	0.01	0.00	0.00	66	1	-70	68

Details of SpliceAI INFO field (as provided in https://github.com/Illumina/SpliceAI):

DS_AG Delta score (acceptor gain)
DS_AL Delta score (acceptor loss)
DS_DG Delta score (donor gain)
DS_DL Delta score (donor loss)
DP_AG Delta position (acceptor gain)
DP_AL Delta position (acceptor loss)
DP_DG Delta position (donor gain)
DP_DL Delta position (donor loss)

Delta score of a variant, defined as the maximum of (DS_AG, DS_AL, DS_DG, DS_DL), ranges from 0 to 1 and can be interpreted as the probability of the variant being splice-altering, with the following cutoffs: 0.2 (high recall), 0.5 (recommended), and 0.8 (high precision). Delta position conveys information about the location where splicing changes relative to the variant position (positive values are downstream of the variant, negative values are upstream).

Supplementary Table 3. Branchpoint annotation and prediction of the effects of the *MYBPC3* c.3331−26T>G variant on branchpoint selection using four *in silico* predictors.

		Branchpointer				BPP				LaBranchoR		RNABP	
Position	Ref nt	c.3331−26T		c.3331−26G		c.3331−26T		c.3331−26G		c.3331−26T	c.3331−26G	c.3331−26T	c.3331−26G
		Branchpoint probability	U2 binding energy	Branchpoint probability	U2 binding energy	Branchpoint motif	z-score	Branchpoint motif	z-score	Score	Score	Branchpoint probability	Branchpoint probability
−20	C	0.102	0.2	0.102	0.2	GCCTCCC	-0.183	GCCTCCC	-0.189	0.049	0.047	2.23	0.84
−21	C	0.310	0.6	0.314	0.0	**T**GCCTCC	-0.183	**G**GCCTCC	-0.189	0.473	0.266	3.05	0.96
−22	T	0.044	1.0	0.044	0.0	A**T**GCCTC	-0.183	A**G**GCCTC	-0.189	0.058	0.018	3.47	1.51
−23	C	0.078	1.6	0.073	0.9	GA**T**GCCT	-0.183	GA**G**GCCT	-0.189	0.208	0.088	5.60	2.13
−24	C	0.131	1.6	0.085	0.9	AGA**T**GCC	-0.183	AGA**G**GCC	-0.189	0.458	0.065	3.91	3.87
−25	G	0.052	1.4	0.049	0.3	GAGA**T**GC	-0.183	GAGA**G**GC	-0.189	0.029	0.022	2.25	3.56
−26	T	0.043	0.3	0.061	0.3	AGAGA**T**G	-0.183	AGAGA**G**G	-0.189	0.072	0.011	3.22	2.02
−27	A	0.191	0.5	0.165	0.1	CAGAGA**T**	-0.183	CAGAGA**G**	-0.189	0.298	0.276	36.64	33.68
−28	G	0.058	0.5	0.048	0.5	CCAGAGA	-0.183	CCAGAGA	-0.189	0.025	0.031	2.69	1.38
−29	A	0.219	0.5	0.296	0.5	CCCAGAG	-0.183	CCCAGAG	-0.189	0.513	0.571	41.63	39.19
−30	G	0.054	0.2	0.045	0.2	GCCCAGA	-0.183	GCCCAGA	-0.189	0.033	0.036	3.01	3.0
−31	A	0.359	0.0	0.293	0.0	GGCCCAG	5.477	GGCCCAG	5.292	0.169	0.222	43.18	39.86
−32	C	0.034	0.0	0.026	0.0	TGGCCCA	-0.183	TGGCCCA	-0.189	0.009	0.010	2.36	2.35
−33	C	0.065	0.5	0.050	0.5	CTGGCCC	-0.183	CTGGCCC	-0.189	0.013	0.011	0.99	1.09
−34	C	0.022	0.1	0.017	0.1	CCTGGCC	-0.183	CCTGGCC	-0.189	0.016	0.009	0.96	1.17

The position relative to the 3' splice site is indicated. Prediction scores for nucleotides at positions from −20 to −34 are shown. Ref nt - reference nucleotide. Software developers recommended the following guidelines to identify the branchpoint (BP): Branchpointer - nucleotide with a probability score higher than 0.52, BPP - BP motif with the highest z-score in the BP area, LaBranchoR - nucleotide with the highest score, RNABP - nucleotide the highest probability. The highest score assigned by each prediction tool to reference nucleotides is highlighted in grey, potentially representing the natural branchpoint of intron 30, probably located at −31A or −29A. The four *in silico* tools consistently suggested that the *MYBPC3* c.3331−26T>G variant does not have a considerable effect on the branchpoint scores obtained by the top-ranked nucleotides −31A or −29A. LaBranchoR generated a minority prediction suggesting that the variant substantially reduces the scores obtained by nucleotides located between −21 and −24. In the BPP heptamer motifs, reference (T) and alternative (G) nucleotides are underlined and highlighted in green and red, respectively.

Supplementary Table 4. Standardized nomenclature *of MYBPC3* misspliced transcripts induced by the variant c.3331−26T>G at both RNA and deduced protein level.

Short name	Standardized nomenclature	
	RNA level (Reference Sequence: NM_000256.3)	Protein level (Reference Sequence: NP_000247.2)
cRi30	r.3330_3331ins3330+1 _3331-1	p.Glu1111Valfs*37
pRi30/AG1	r.3330_3331ins3331-92_3331-1	p.Glu1111*
pRi30/AG2	r.3330_3331ins3330-89 _3331-1	p.Glu1111Glyfs*108

cRi30 - complete retention of intron 30
pRi30/AG1 - partial retention of intron 30 by the use AG1
pRi30/AG2 - partial retention of intron 30 by the use AG2

Supplementary Table 5. Prediction of potential splice acceptor sites in *MYBPC3* intron 30.

Splice acceptor site	Sequence motif		*MYBPC3* Reference Sequence		*MYBPC3* c.3331−26T>G	
	intron (-7 -6 -5 -4 -3 -2 -1)	exon	FSPLICE score (threshold= 4.17)	SPLM score (range: 1-100)	FSPLICE score (threshold= 4.17)	SPLM score (range: 1-100)
Canonical	ccccc**ag**	GAGTG	11.47	78	10.57	66
Cryptic AG1	tccat**ag**	TAGGG	8.07	52	8.07	52
Cryptic AG2	atagt**ag**	GGGTG	5.33	26	5.33	26

Using the complete *MYBPC3* intron 30 sequence (including the i30-E31 junction) as an input (from both reference and c.3331−26T>G sequences) all potential splice acceptor sites were predicted using FSPLICE and SPLM platforms. In addition to the canonical splice acceptor site of intron 30, both FSPLICE and SPLM algorithms detected the AG1 and AG2 cryptic splice acceptor sites, that were used both in minigene-transfected cells and patients´ blood cells for the generation of pRi30/AG1 and pRi30/AG2 *MYBPC3* misspliced transcripts with partial retention of intron 30. Twelve nucleotide sequence motif is shown for each detected acceptor site. Essential **ag** dinucleotides are shown in bold. Both bioinformatics tools considered that AG1 is a stronger acceptor site than AG2. The cryptic site prediction scores for AG1 and AG2 were the same for both reference or c.3331−26T>G sequences, whereas the canonical site prediction scores were slightly reduced for the c.3331−26T>G sequence.

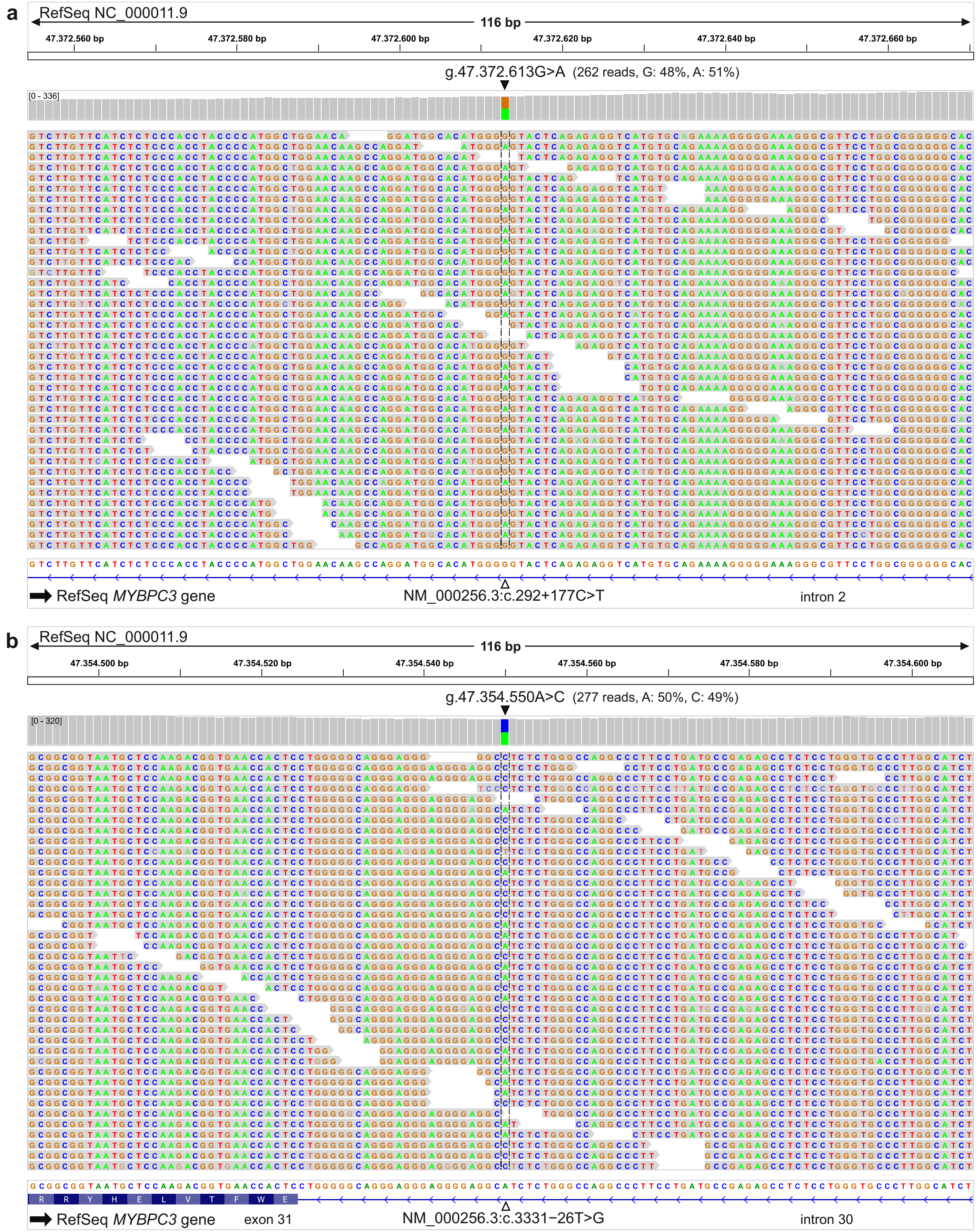

Supplementary Fig. 1. Identification of two *MYBPC3* intronic variants in the index patient. Alignments of massively parallel sequencing reads to the reference genome (RefSeq NC_000011.9) are shown at two *MYBPC3* genomic positions (116-bp scale) revealing two nucleotide substitutions located in intron 2 (**a**, c.292+177C>T) and intron 30 (**b**, c.3331−26T>G). The *MYBPC3* gene was targeted by probes covering about 21 kb, corresponding to the complete sequence between exon 1 and the last exon 35. After classification of sequencing data and filtering out common variants, only these two rare intronic variants were identified in the *MYBPC3* gene, in the heterozygous state. Genomic (black triangles) and coding DNA (white triangles) variant names are indicated and the percentage of reads corresponding to each alternative nucleotide are shown with round brackets. Grey vertical bars represent the coverage of each nucleotide and the coverage range is annotated with square brackets. Coverage bars with two colors revealed the presence of both the reference and the alternative nucleotide in the reads covering that position. Exon-intron structure is represented with blue boxes and lines, respectively. Black arrows pointing right indicate that the positive strand of RefSeq is shown. Blue arrows pointing left indicate the direction of transcription from the negative strand. The figure was assembled using scalable vector graphic files exported from the IGV application and allow unlimited zoom in when displayed on screens.

Alamut Visual Splicing Predictions

Gene: **MYBPC3** - Transcript: **NM_000256.3** - Variant: **c.292+177C>T**
Analysis range: **c.258 (Exon 2) - c.358 (Exon 3) [723 bps]**

Donor Sites

	SSF [0-100]	MaxEnt [0-12]	NNSPLICE [0-1]	GeneSplicer [0-24]
Threshold	*≥ 70*	*≥ 0*	*≥ 0.4*	*≥ 0*
Exon 2 – c.261	= 72.64	= 1.99		= 2.25
Exon 2 – c.279	= 72.09	= 2.53		= 2.27
Exon 2 – c.288	= 87.67			
**Exon 2 – c.292** ℕ	= 94.67	= 10.77	= 0.98	= 8.91
Intron 2 – c.292+27	= 71.88	= 3.53		= 0.58
Intron 2 – c.292+86		= 3.62		
Intron 2 – c.292+211	= 77.90	= 6.59	= 0.71	**1.19 ⇒ 1.35** *(+13.7%)*
Intron 2 – c.292+215	= 76.10	= 3.27		
Intron 2 – c.293-272	= 70.14			
Intron 2 – c.293-185	= 77.39	= 3.37		= 6.15
Intron 2 – c.293-178	= 79.47			
Intron 2 – c.293-41	= 81.61	= 6.32	= 0.58	= 3.07
Exon 3 – c.336	= 72.61			

ℕ**atural Splice Site**

Acceptor Sites

	SSF [0-100]	MaxEnt [0-16]	NNSPLICE [0-1]	GeneSplicer [0-21]
Threshold	*≥ 70*	*≥ 0*	*≥ 0.4*	*≥ 0*
Exon 2 – c.280		= 0.06		
Intron 2 – c.292+77	= 84.94	= 6.83	= 0.71	
Intron 2 – c.292+134	= 77.04	= 7.03		**5.55 ⇒ 5.49** *(-1.1%)*
Intron 2 – c.292+205	= 80.91	= 5.81	= 0.86	**5.00 ⇒ 5.01** *(+0.2%)*
Intron 2 – c.292+259	= 83.43	= 12.77	= 0.95	**13.69 ⇒ 13.74** *(+0.3%)*
Intron 2 – c.292+287	= 71.35			
Intron 2 – c.293-160	= 74.86			
Intron 2 – c.293-89	= 81.18	= 8.10	= 0.58	= 4.42
Intron 2 – c.293-65	= 71.98	= 2.04		
**Exon 3 – c.293** ℕ	= 82.95	= 8.20	= 0.72	= 11.92

ℕ**atural Splice Site**

© Interactive Biosoftware - Created by Alamut Visual v.2.11.0 on 07/06/2021

Alamut Visual Splicing Predictions

Gene: **MYBPC3** - Transcript: **NM_000256.3** - Variant: **c.3331-26T>G**
Analysis range: **c.3231 (Exon 30) - c.3452 (Exon 31) [441 bps]**

Donor Sites

	SSF [0-100]	MaxEnt [0-12]	NNSPLICE [0-1]	GeneSplicer [0-24]
Threshold	*≥ 70*	*≥ 0*	*≥ 0.4*	*≥ 0*
Exon 30 – c.3296		= 0.38		
**Exon 30 – c.3330** ℕ	= 81.04	= 8.27	= 0.99	= 11.12
Intron 30 – c.3331-94				**3.62 ⇒ 3.78** *(+4.3%)*
Exon 31 – c.3432		= 1.95		

ℕ**atural Splice Site**

Acceptor Sites

	SSF [0-100]	MaxEnt [0-16]	NNSPLICE [0-1]	GeneSplicer [0-21]
Threshold	*≥ 70*	*≥ 0*	*≥ 0.4*	*≥ 0*
Intron 30 – c.3330+42	= 71.57	= 6.39		
Intron 30 – c.3330+51		= 0.69		
Intron 30 – c.3331-92		= 4.67		**3.17 ⇒ 2.91** *(-8.4%)*
Intron 30 – c.3331-89		= 1.79	= 0.68	
Intron 30 – c.3331-45	= 76.31			
**Exon 31 – c.3331** ℕ	= 82.09	= 12.05	= 0.97	**10.67 ⇒ 9.42** *(-11.7%)*
Exon 31 – c.3352	= 70.03			
Exon 31 – c.3383	= 70.81	= 3.95		
Exon 31 – c.3423	= 81.07	= 4.36	= 0.42	
Exon 31 – c.3427		= 0.98		
Exon 31 – c.3444		= 2.18		

ℕ**atural Splice Site**

© Interactive Biosoftware - Created by Alamut Visual v.2.11.0 on 31/03/2021

Supplementary Fig. 2. Output of the Alamut Visual splicing predictions. The predicted splicing consequences of the *MYBPC3* intronic variants c.292+177C>T and c.3331−26T>G are shown. The analysis range (723 bp and 441 bp) was adjusted for each variant to include predictions for the natural splice sites (N). The red arrows pointing right indicate the score prediction change obtained from the reference to the alternative sequence. The equal signs indicate that the same score was obtained for the reference or the alternative sequence.

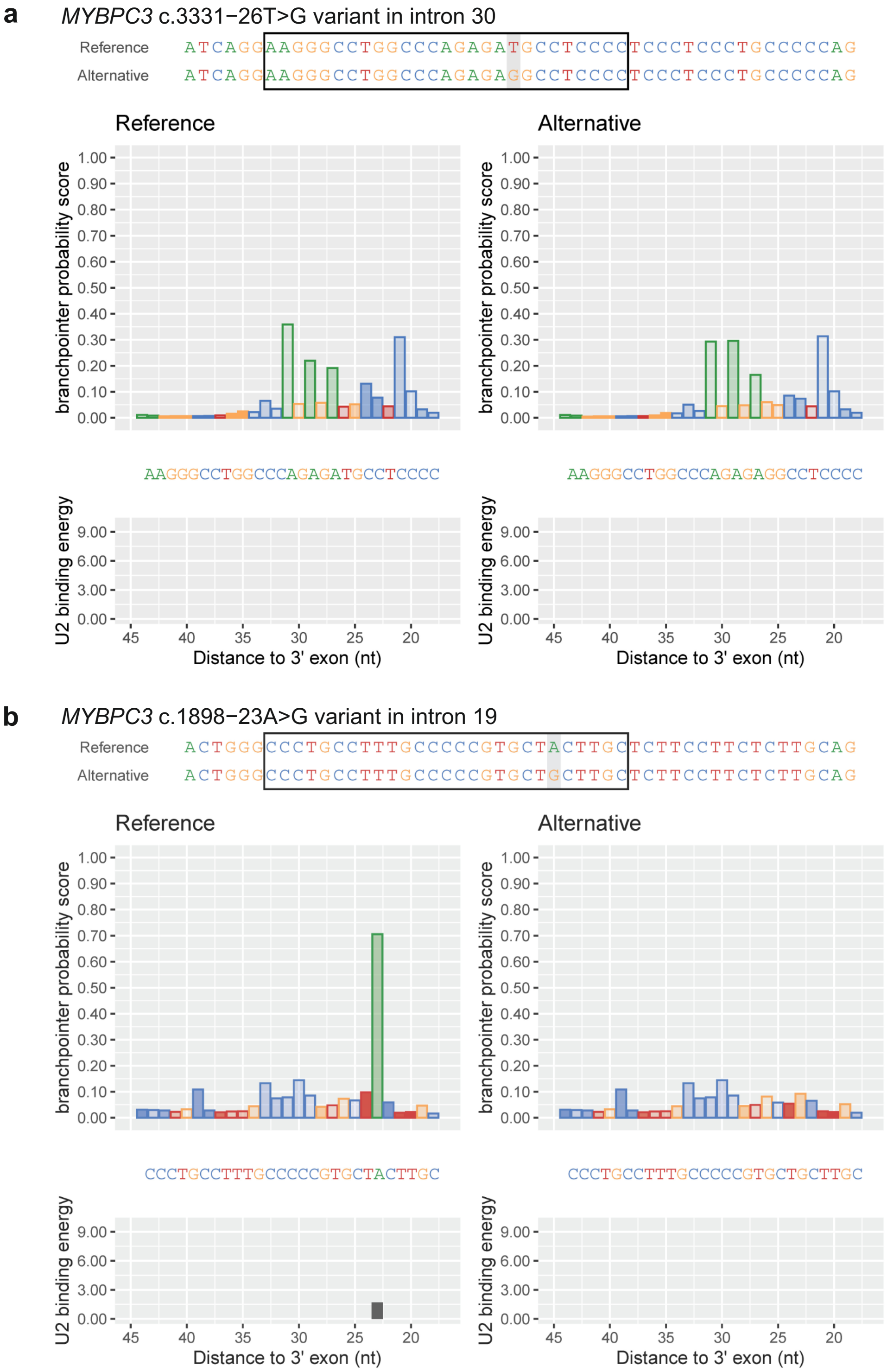

Supplementary Fig. 3. Annotation of splicing branchpoints and effects of two *MYBPC3* intronic variants on branchpoint selection. The graphical prediction outputs generated by Branchpointer, a machine-learning based model of branchpoints, are shown for two *MYBPC3* intronic nucleotide substitutions: the variant object of this study located in intron 30 (**a**, c.3331−26T>G) and a previously reported splice-altering variant in intron 19 (**b**, c.1898−23A>G). The software analysis range included the region between −18 to −44 relative to the splice acceptor site. These nucleotides are boxed in the sequence and represented in the probability graph. The main panel displays the probability scores of each site within the branchpoint window. The opacity of the bars is representative of relative U2 binding energy (darker = stronger), and the lower panel shows U2 binding energy for all sites above the probability cutoff. **a** The software predicts several potential branchpoints in reference intron 30. The maximum branchpoint score was obtained for the nucleotide A at position −31 (0.359). This score, at the same −31 position, was only slightly reduced in the alternative sequence c.3331−26T>G (0.293). However, none of the scores obtained from reference or alternative sequences exceed the probability cut-off of 0.52. The U2 binding is not represented in this case, since the U2 binding energy can be used as a measurement of branchpoint strength only when the probability score is above the cut-off. **b** The Branchpointer results indicated that the splice-altering *MYBPC3* c.1898−23A>G variant suppresses the branchpoint located at c.1898−23A.

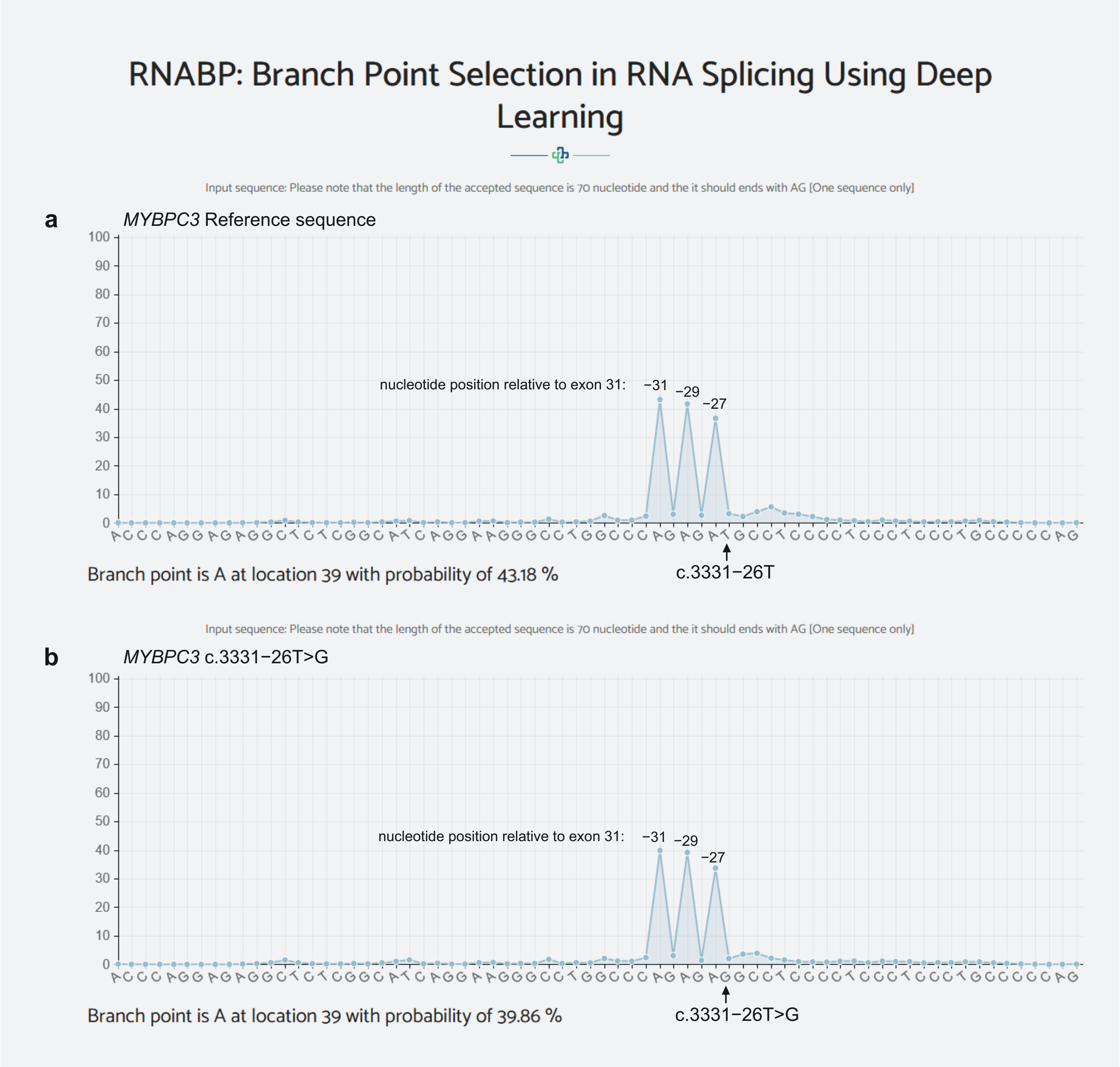

Supplementary Fig. 4. Effect of the *MYBPC3* c.3331−26T>G variant on branchpoint selection in RNA splicing using the deep-learning prediction tool RNABP. The last 70 nucleotides of intron 30 corresponding to the reference (**a**) or c.3331−26T>G (**b**) sequences were used as inputs. In the *MYBPC3* reference sequence (a), the RNABP program identified three probable A nucleotides to be used as the branchpoint, located at positions −31, −29 and −27 from exon 31 (equivalent to locations 39, 41 and 43 counting the 70 represented nucleotides from the left, respectively), close to the c.3331−26T>G variant. In the reference sequence, the software estimated the following probability of each nucleotide A to be a branchpoint: 43.18%, 41.63% and 36.64%, respectively. In the *MYBPC3* c.3331−26T>G sequence (b), the RNABP program estimated a slightly reduced probability values for each nucleotide A: 39.86%, 39.19% and 33.68%, respectively.

Reference *MYBPC3* minigene plasmid 1446

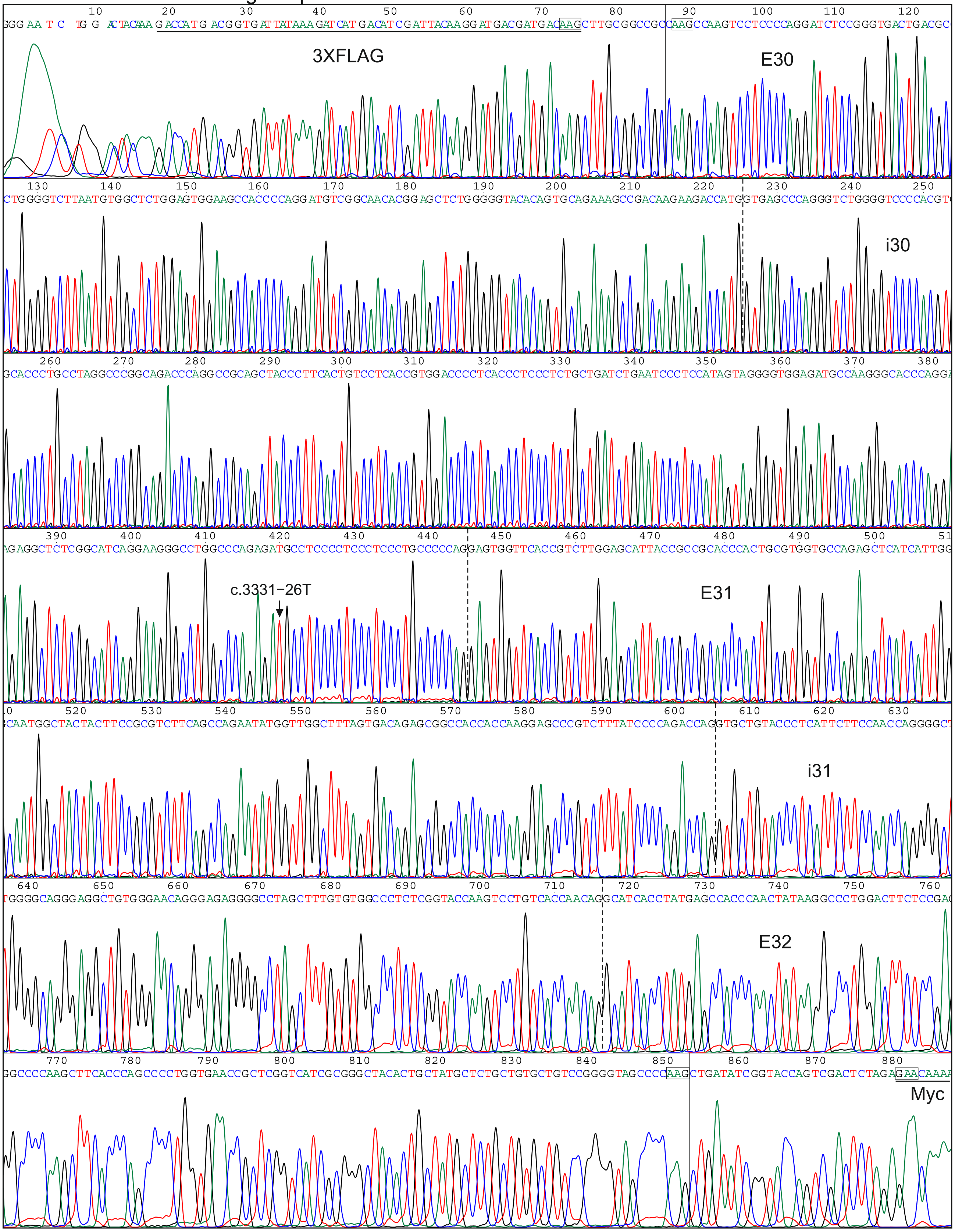

Supplementary Fig. 5. Full insert DNA sequencing chromatogram of the reference *MYBPC3* minigene plasmid 1446. The forward sequence obtained with the sequencing primer 480 is shown. The plasmid insert sequence was also verified with the reverse sequence obtained with primer 481. The boundary between the FLAG-epitope vector sequence and the first *MYBPC3* minigene exon (E30) is indicated with a vertical solid line. The first nucleotide of E30 was removed during the PCR amplification step to allow cloning the first complete codon of E30 (boxed) in-frame with the FLAG epitope. The last codon of the FLAG-tag is boxed. The boundary between the last codon of E32 (boxed) and the start of the vector sequence containing the Myc-tag epitope is also shown with a vertical solid line. The end of the FLAG-tag and the start of the Myc-tag coding sequences are both underlined. The first codon of the Myc-tag is boxed. The boundaries between exons (E) and introns (i) are shown with vertical dashed lines. The arrow marks the position of a single red chromatogram peak corresponding to the reference T nucleotide at c.3331−26T.

Mutant *MYBPC3* minigene plasmid 1447

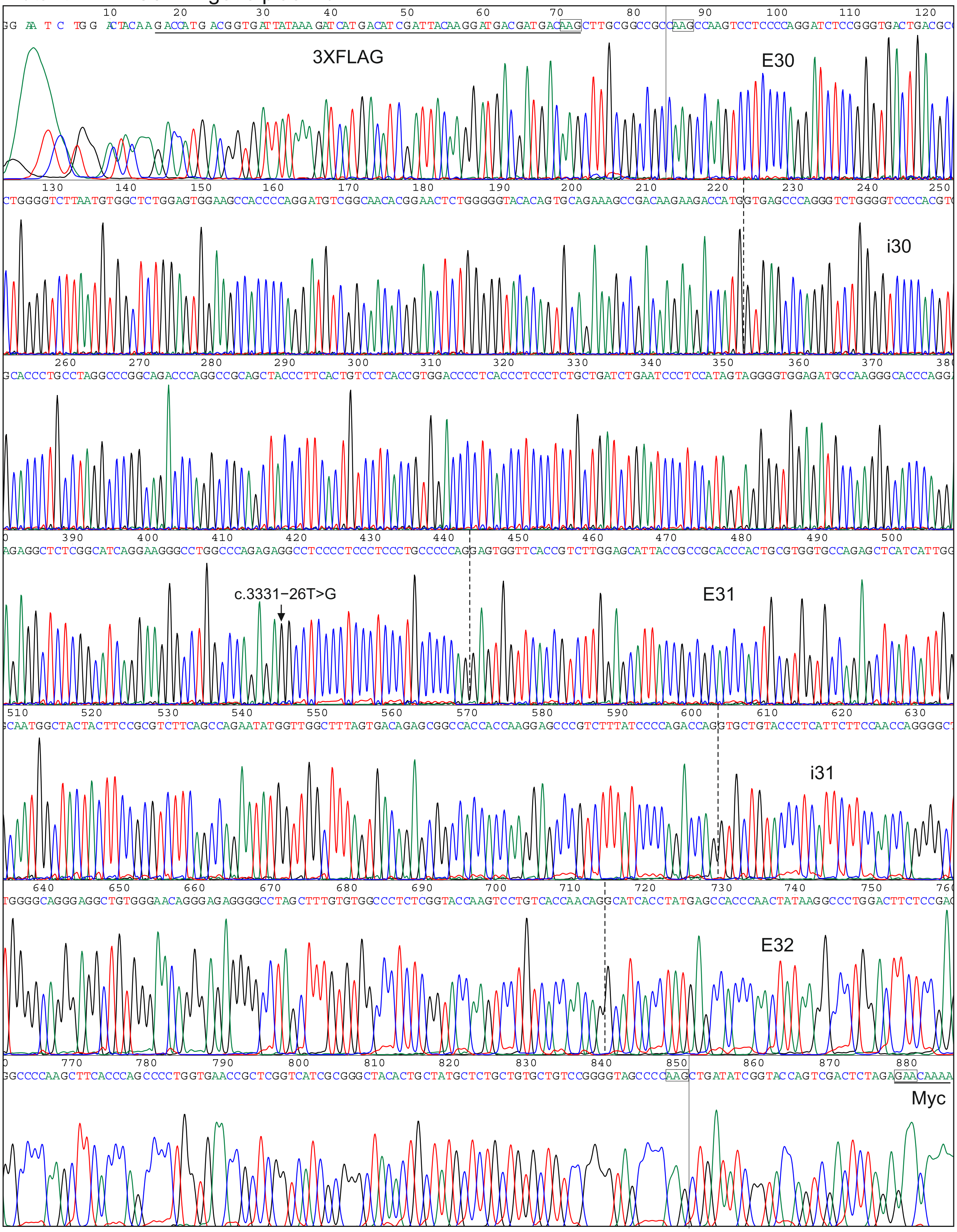

Supplementary Fig. 6. Full insert DNA sequencing chromatogram of the mutant *MYBPC3* minigene plasmid 1447. Figure annotations are described in the previous Supplementary Fig. 5. The arrow marks the position of a single black chromatogram peak corresponding to the alternative G nucleotide of the *MYBPC3* c.3331−26T>G substitution.

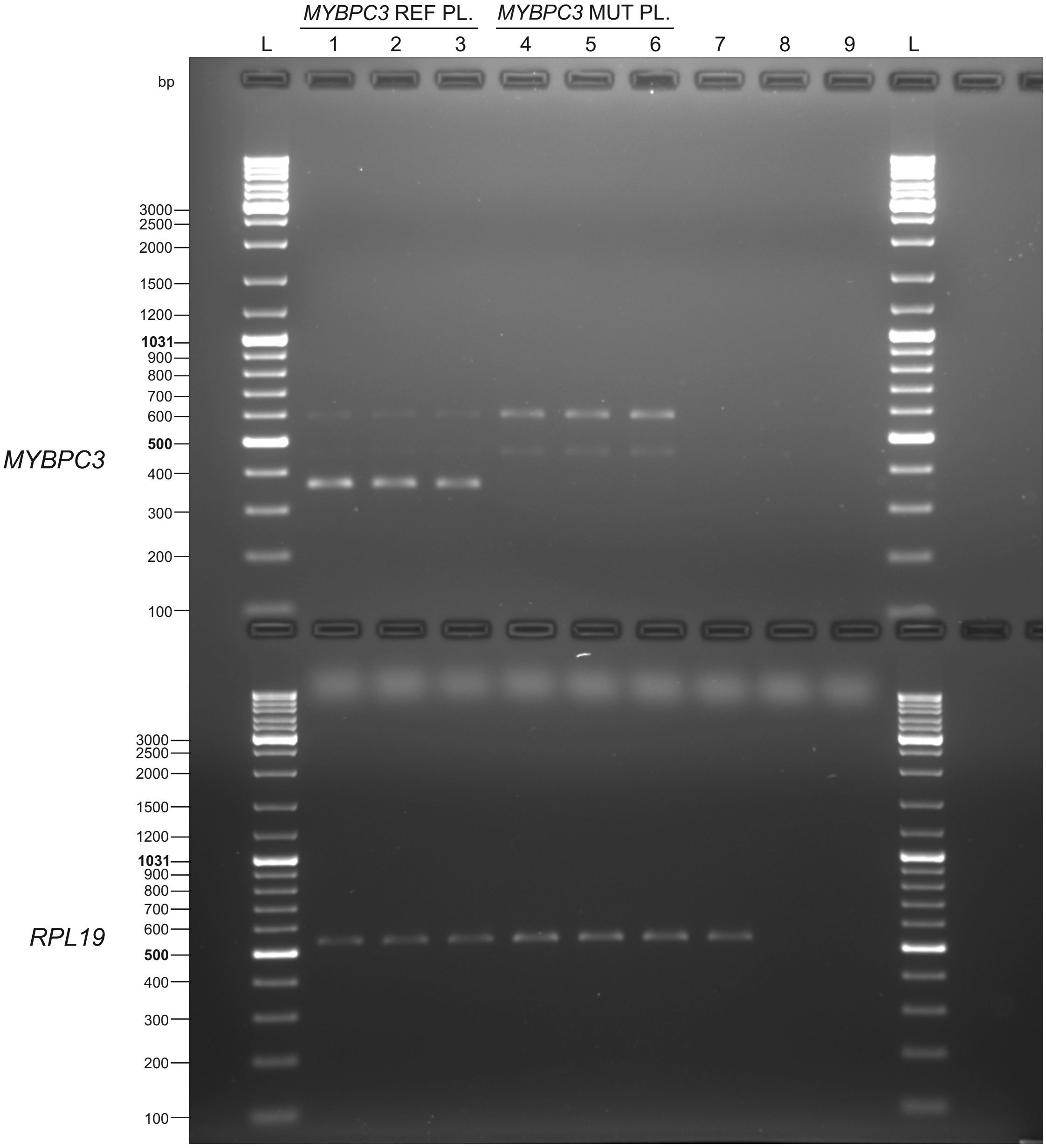

Supplementary Fig. 7. Full size RT-PCR gel image of Fig. 3. An additional picture of the same gel presented in Fig. 3c was taken with the Versadoc's optical zoom set to a lower value to show the entire gel. *MYBPC3* and *RPL19* RT-PCR products were loaded in the upper and lower part of the same agarose gel.

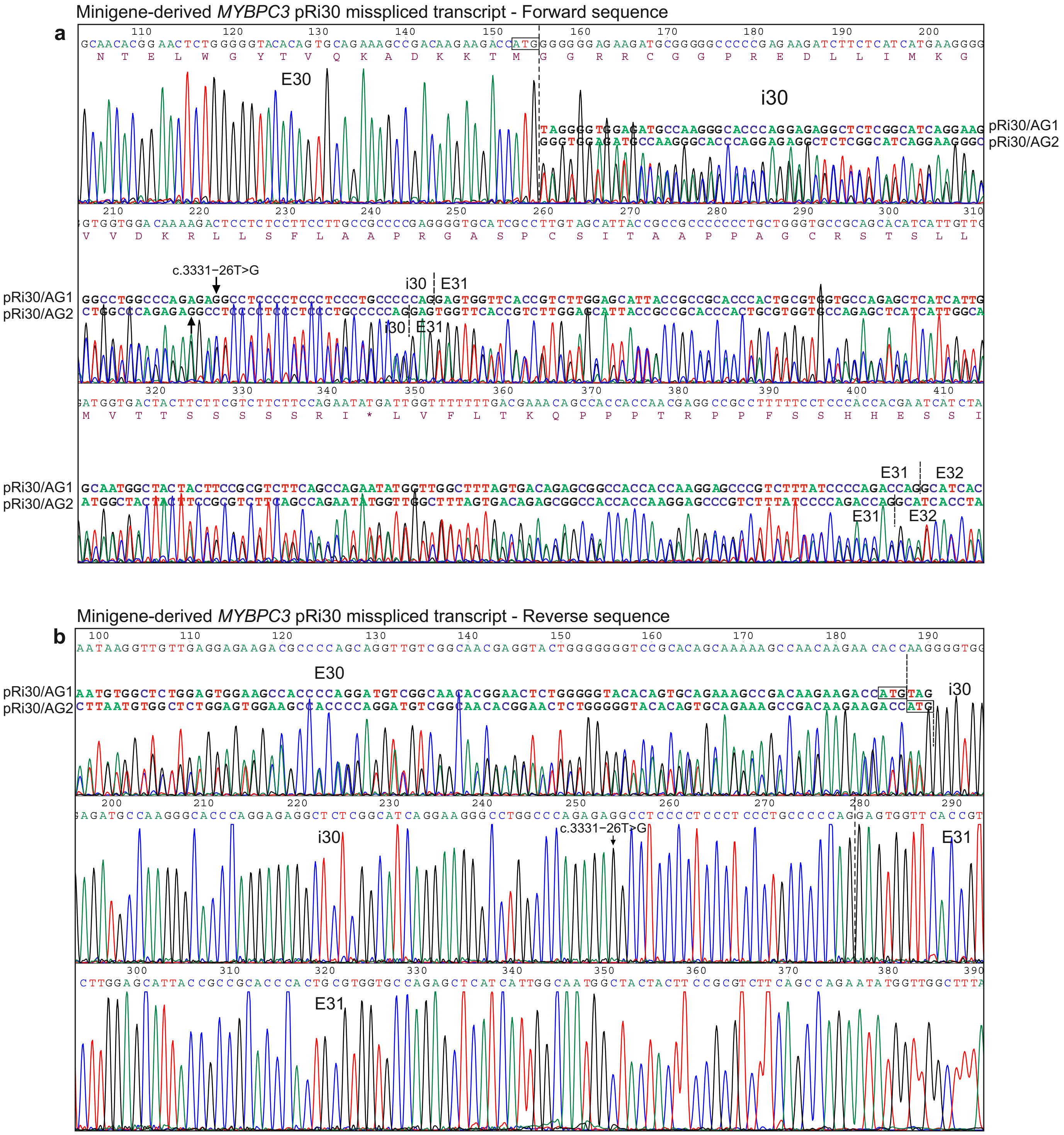

Supplementary Fig. 8. Sequence analysis of the purified 450-bp RT-PCR band derived from the mutant minigene *MYBPC3* c.3331−26T>G. Forward (a) and reverse (b) sequencing chromatograms are shown. Exons (E) and introns (i) junctions are marked with dashed lines. The analysis of both sequences indicated that two similar-sized RT-PCR products were co-localized in the 450-bp gel band, resulting in overlapping chromatogram peaks in the middle part of the sequence. The point where the sequences became duplicated corresponds to the alternative use of two consecutive AG dinucleotides present in the middle of intron 30 (see Fig. 4) that were used as splice acceptor sites in association with the canonical dinucleotide GT of the splice donor site of intron 30, resulting in two misspliced transcripts with partial retention of intron 30 (pRi30/AG1, RT-PCR product length: 453 bp; and pRi30/AG2, RT-PCR product length 450 bp). In the forward chromatogram (a), the sequence turned duplicated after the last ATG codon (boxed) of exon 30. Hereafter, the nucleotide sequence deduced by Chromas is not valid since it could not interpret overlapping chromatogram peaks. The expected misspliced mRNA sequences (pRi30/AG1 and pRi30/AG2) have been typed over the chromatogram and each overlapping nucleotide peak was verified to correspond to either the sequence of pRi30/AG1 or pRi30/AG2. The reverse sequence (b) showed a single chromatogram sequence through the second half of i30 and E31, which turned into a duplicated overlapping sequence in the junction of i30 and E30. The last codon of E30, ATG, is boxed in both sequences. The *MYBPC3* c.3331−26T>G nucleotide variant is present in both misspliced variants, pRi30/AG1 and pRi30/AG2, and is marked with vertical arrows in the forward and reverse sequences.

a cRi30 *MYBPC3*

```
3193 aagccaagtcctccccaggatctccgggtgactgacgcctggggtcttaatgtggctctg 3252 E30
1065 K  P  S  P  P  Q  D  L  R  V  T  D  A  W  G  L  N  V  A  L   1084
3253 gagtggaagccaccccaggatgtcggcaacacggagctctggggtacacagtgcagaaa 3312
1085 E  W  K  P  P  Q  D  V  G  N  T  E  L  W  G  Y  T  V  Q  K   1104
3313 gccgacaagaagaccatggtgagcccagggtctggggtccccacgtgcaccctgcctagg 3372 i30
1105 A  D  K  K  T  M  V  S  P  G  S  G  V  P  T  C  T  L  P  R   1124
3373 cccggcagacccaggccgcagctacccttcactgtcctcaccgtggacccctcaccctcc 3432
1125 P  G  R  P  R  P  Q  L  P  F  T  V  L  T  V  D  P  S  P  S   1144
3433 ctctgctgatctgaatccctccatagtaggggtggagatgccaagggcacccaggagagg 3492
1145 L  C  * 1146
3493 ctctcggcatcaggaagggcctggcccagagaggcctcccctccctccctgcccccagga 3552 E31
3553 gtggttcaccgtcttggagcattaccgccgcacccactgcgtggtgccagagctcatcat 3612
3613 tggcaatggctactacttccgcgtcttcagccagaatatggttggctttagtgacagagc 3672
3673 ggccaccaccaaggagcccgtctttatccccagaccaggcatcacctatgagccacccaa 3732 E32
3733 ctataaggccctggacttctccgaggccccaagcttcacccagcccctggtgaaccgctc 3792
3793 ggtcatcgcgggctacactgctatgctctgctgtgctgtccggggtagccccaagcccaa 3852 E33
3853 gatttcctggttcaagaatggcctggacctgggagaagacgcccgcttccgcatgttcag 3912
3913 caagcagggagtgttgactctggagattagaaagccctgcccctttgacgggggcatcta 3972
3973 tgtctgcagggccaccaacttacagggcgaggcacggtgtgagtgccgcctggaggtgcg 4032
4033 agtgcctcagtga 4045                                                E34
```

b pRi30/AG1 *MYBPC3*

```
3193 aagccaagtcctccccaggatctccgggtgactgacgcctggggtcttaatgtggctctg 3252 E30
1065 K  P  S  P  P  Q  D  L  R  V  T  D  A  W  G  L  N  V  A  L   1084
3253 gagtggaagccaccccaggatgtcggcaacacggagctctggggtacacagtgcagaaa 3312
1085 E  W  K  P  P  Q  D  V  G  N  T  E  L  W  G  Y  T  V  Q  K   1104
3313 gccgacaagaagaccatgtagggtggagatgccaagggcacccaggagaggctctcggc 3372 i30
1105 A  D  K  K  T  M  *  1110
3373 atcaggaagggcctggcccagagaggcctcccctccctccctgcccccaggagtggttca 3432 E31
3433 ccgtcttggagcattaccgccgcacccactgcgtggtgccagagctcatcattggcaatg 3492
3493 gctactacttccgcgtcttcagccagaatatggttggctttagtgacagagcggccacca 3552
3553 ccaaggagcccgtctttatccccagaccaggcatcacctatgagccacccaactataagg 3612 E32
3613 ccctggacttctccgaggccccaagcttcacccagcccctggtgaaccgctcggtcatcg 3672
3673 cgggctacactgctatgctctgctgtgctgtccggggtagccccaagcccaagatttcct 3732 E33
3733 ggttcaagaatggcctggacctgggagaagacgcccgcttccgcatgttcagcaagcagg 3792
3793 gagtgttgactctggagattagaaagccctgcccctttgacgggggcatctatgtctgca 3852
3853 gggccaccaacttacagggcgaggcacggtgtgagtgccgcctggaggtgcgagtgcctc 3912 E34
3913 agtga 3917
```

c pRi30/AG2 *MYBPC3*

```
3193 aagccaagtcctccccaggatctccgggtgactgacgcctggggtcttaatgtggctctg 3252 E30
1065 K  P  S  P  P  Q  D  L  R  V  T  D  A  W  G  L  N  V  A  L   1084
3253 gagtggaagccaccccaggatgtcggcaacacggagctctggggtacacagtgcagaaa 3312
1085 E  W  K  P  P  Q  D  V  G  N  T  E  L  W  G  Y  T  V  Q  K   1104
3313 gccgacaagaagaccatgggtggagatgccaagggcacccaggagaggctctcggcatc 3372 i30
1105 A  D  K  K  T  M  G  W  R  C  Q  G  H  P  G  E  A  L  G  I   1124
3373 aggaagggcctggcccagagaggcctcccctccctccctgcccccaggagtggttcaccg 3432 E31
1125 R  K  G  L  A  Q  R  G  L  P  S  L  P  A  P  R  S  G  S  P   1144
3433 tcttggagcattaccgccgcacccactgcgtggtgccagagctcatcattggcaatggct 3492
1145 S  W  S  I  T  A  A  P  T  A  W  C  Q  S  S  S  L  A  M  A   1164
3493 actacttccgcgtcttcagccagaatatggttggctttagtgacagagcggccaccacca 3552
1165 T  T  S  A  S  S  A  R  I  W  L  A  L  V  T  E  R  P  P  P   1184
3553 aggagcccgtctttatccccagaccaggcatcacctatgagccacccaactataaggccc 3612 E32
1185 R  S  P  S  L  S  P  D  Q  A  S  P  M  S  H  P  T  I  R  P   1204
3613 tggacttctccgaggccccaagcttcacccagcccctggtgaaccgctcggtcatcgcgg 3672
1205 W  T  S  P  R  P  Q  A  S  P  S  P  W  *    1217
3673 gctacactgctatgctctgctgtgctgtccggggtagccccaagcccaagatttcctggt 3732 E33
3733 tcaagaatggcctggacctgggagaagacgcccgcttccgcatgttcagcaagcagggag 3792
3793 tgttgactctggagattagaaagccctgcccctttgacgggggcatctatgtctgcaggg 3852
3853 ccaccaacttacagggcgaggcacggtgtgagtgccgcctggaggtgcgagtgcctcagt 3912 E34
3913 ga 3914
```

Supplementary Fig. 9. Deduced protein translation of *MYBPC3* misspliced transcripts caused by the c.3331−26T>G variant. Deduced protein sequence is shown below the nucleotide sequence starting from E30. The three misspliced transcripts identified in the minigene assay were translated *in silico*: (a) complete retention of i30, cRi30; (b) partial retention of i30 with the use of cryptic acceptor site AG1, pRi30/AG1; and (c) partial retention of i30 with the use of cryptic acceptor site AG2, pRi30/AG2. Nucleotide and amino acid numbers starting at E30 correspond to the full coding and protein reference sequences, respectively. Premature termination codon in each sequence is boxed. The natural stop codon in penultimate E34 is underlined. In the three sequences, the alternative G nucleotide corresponding to the *MYBPC3* c.3331−26T>G variant is shown in bold. Each exon is highlighted in different colors. In the cRi30 sequence, the two consecutive cryptic AG dinucleotides are double-underlined.

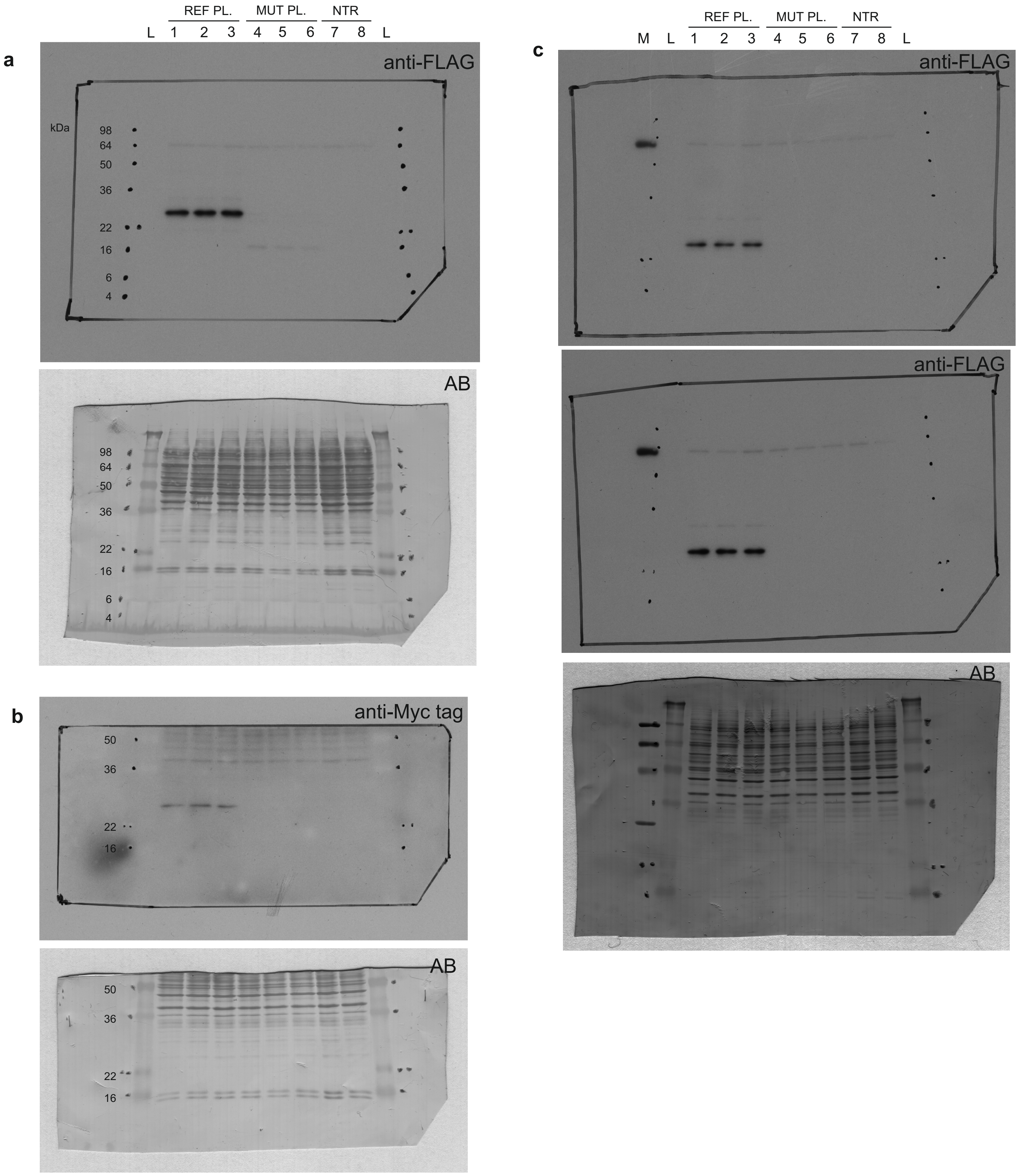

Supplementary Fig. 10. **Full size Western blot film and membrane images of Fig. 5.** The protein ladder SeeBlue Plus2 pre-stained protein standard was included in each membrane (lane L). Molecular weights (kDa) of marker bands are indicated. Films were overlapped to the corresponding membranes to mark the position of each pre-stained marker band. An additional Western blot analysis is shown in **c** using a higher dilution of anti-FLAG (1:25,000 instead of 1:20,000). Two film exposures of about 10 and 20 seconds are shown. In this case, the Amersham Low Molecular Weight (LMW) protein marker (GE Healthcare) was also loaded in lane M. A non-specific band of about 67 kDa corresponding to one of the LMW marker bands was observed. A non-specific band of similar size but weaker was also detected in protein extracts from both transfected and non-transfected cells.

a

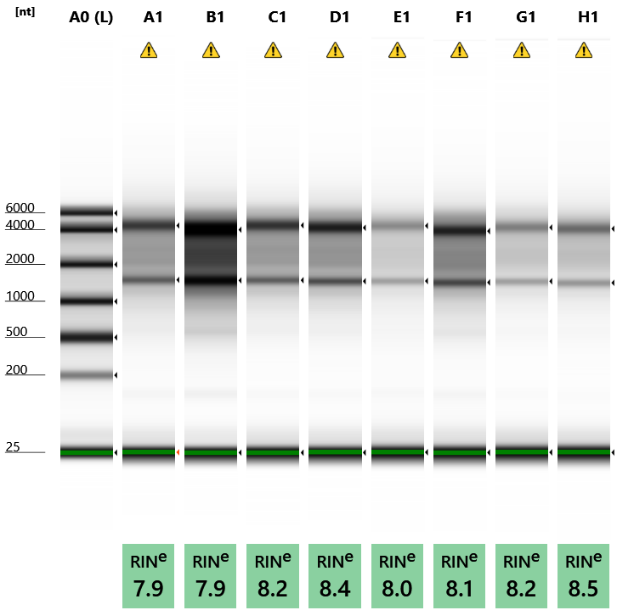

b

Well	RINe	28S/18S (Area)
A0	-	-
A1	7.9	1.7
B1	7.9	1.7
C1	8.2	2.1
D1	8.4	2.0
E1	8.0	2.2
F1	8.1	1.9
G1	8.2	2.4
H1	8.5	2.2

Supplementary Fig. 11. Evaluation of total RNA integrity isolated from whole blood. **a** The results of the TapeStation (Agilent Technologies) automated electrophoresis of total RNA displayed as a gel image shows the separation profile of each sample along with the estimated RNA Integrity Number (RIN) value. The four RNA samples (III.7-lanes A1, E1; III.1-lanes B1, F1; III.4-lanes C1, G1; III.6-lanes D1, H1) isolated and purified from stabilized whole blood were simultaneously run in duplicate lanes using different RNA input volumes: 1.0 µl (A1, B1, C1, D1) or 0.5 µl (E1, F1, G1, H1), on the same RNA ScreenTape (Agilent Technologies). An RNA ladder was loaded in lane A0. RIN values from 1 to 10 can be obtained, where 10 indicates the highest possible RNA quality. **b** The sample information on the right shows, in addition, the ratio 28S/18S.

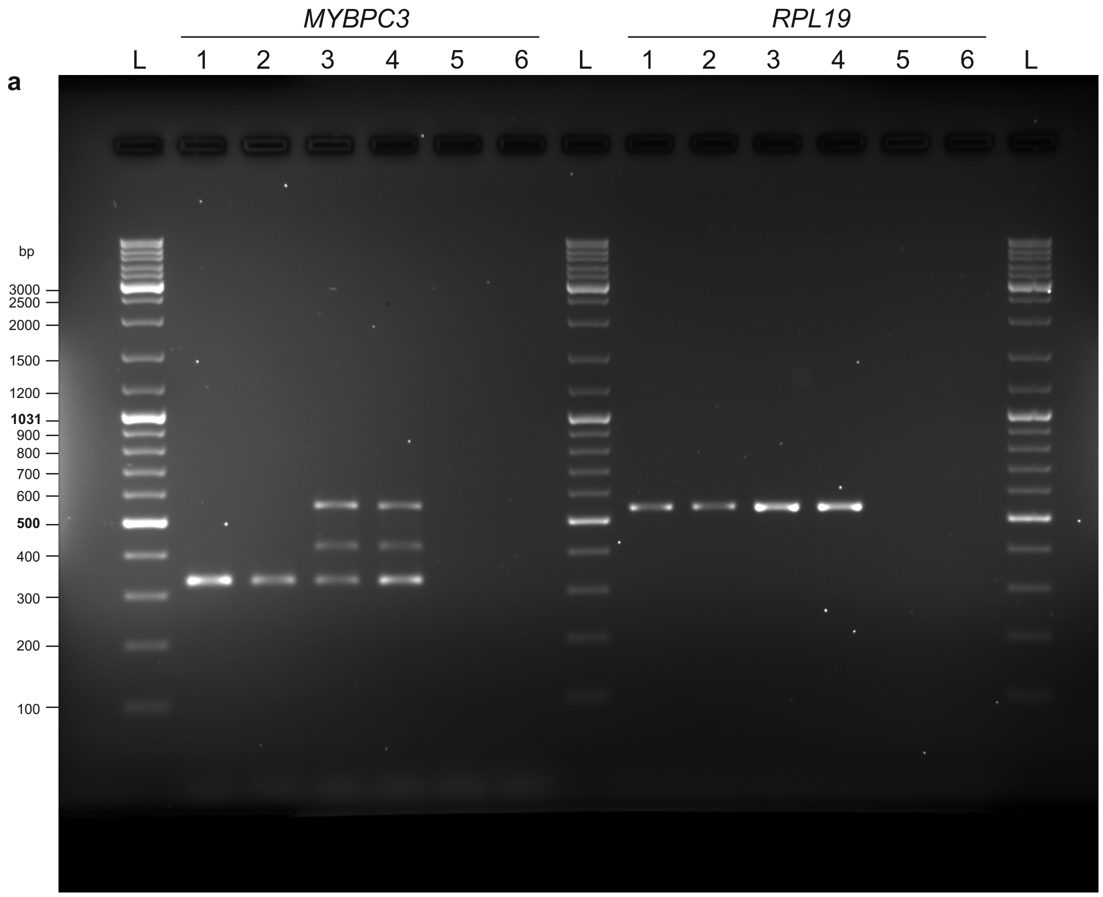

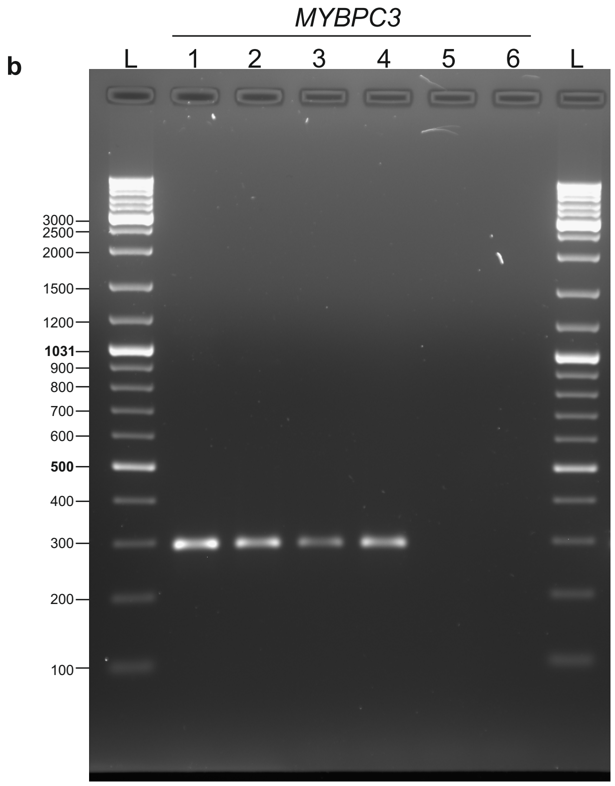

Supplementary Fig. 12. Full size RT-PCR gel images of Fig. 6. a Full image of Fig. 6a. An additional picture of the same gel presented in Fig. 6a was taken with the Versadoc's optical zoom set to lower values to show the entire gel. RT-PCR products from the same cDNA samples targeting the *MYBPC3* mRNA region between exons 30-31 and *RPL19* were loaded in the left and right part of the same gel, respectively. The amplification of *RPL19* was used as a control for the synthesis of cDNA. **b** Full image of Fig. 6b, showing an additional RT-PCR assay targeting the *MYBPC3* mRNA region between exons 2-3 in the same cDNA samples.

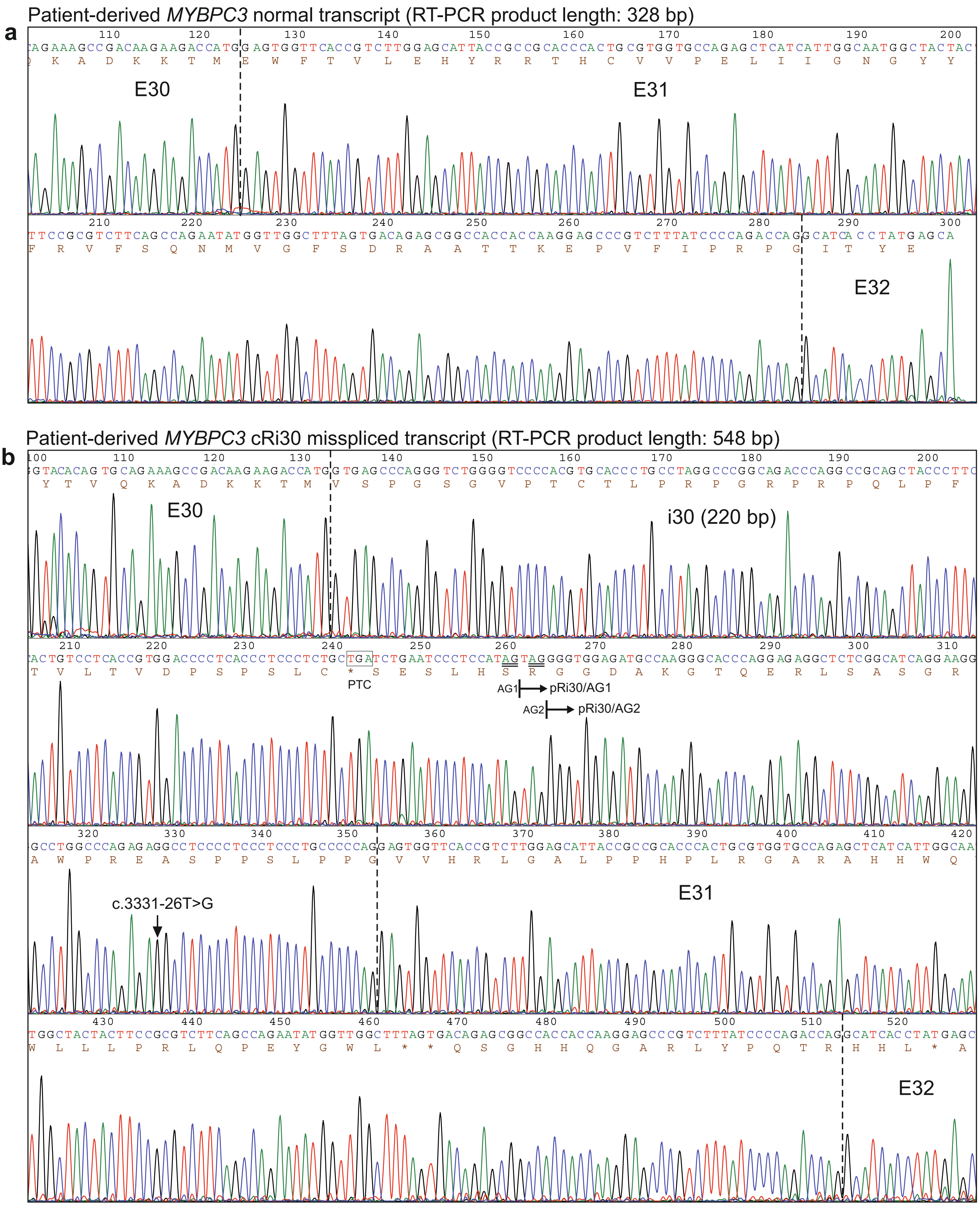

Supplementary Fig. 13. DNA sequencing chromatograms of normal (a) and misspliced cRi30 (b) *MYBPC3* RT-PCR products of patients' RNA. Normal and misspliced RT-PCR products identified in the analysis presented in Fig. 6 were gel-purified and subjected to direct Sanger sequencing. Shown chromatograms were derived from patient III.6 and were identical to those obtained from patient III.4, both heterozygous carriers of the *MYBPC3* c.3331−26T>G variant. The deduced amino acid sequence is shown below the nucleotide sequence, as translated by the Chromas software. The 328-bp RT-PCR band corresponds to normal *MYBPC3* mRNA and the 548-bp band corresponds to a misspliced transcript with the complete retention of intron 30 (cRi30). The presence of a single black-G chromatogram peak corresponding to the nucleotide substitution c.3331−26T>G in the cRi30 transcript is indicated with a vertical arrow. The cRi30 transcript retained i30, but not i31. The exon-exon or exon-intron junctions are marked with vertical dashed lines. PTC - premature termination codon. The patients' blood *MYBPC3* expression assay also has led to the identification of misspliced transcripts of intermediated size generated by partial retention of i30 (pRi30/AG1 and pRi30/AG2) with the use of two pre-existing consecutive AG dinucleotides (double underlined) acting as cryptic splice acceptor sites (see Supplementary Fig. 14), as previously shown by the minigene expression assay (see Supplementary Fig. 8).

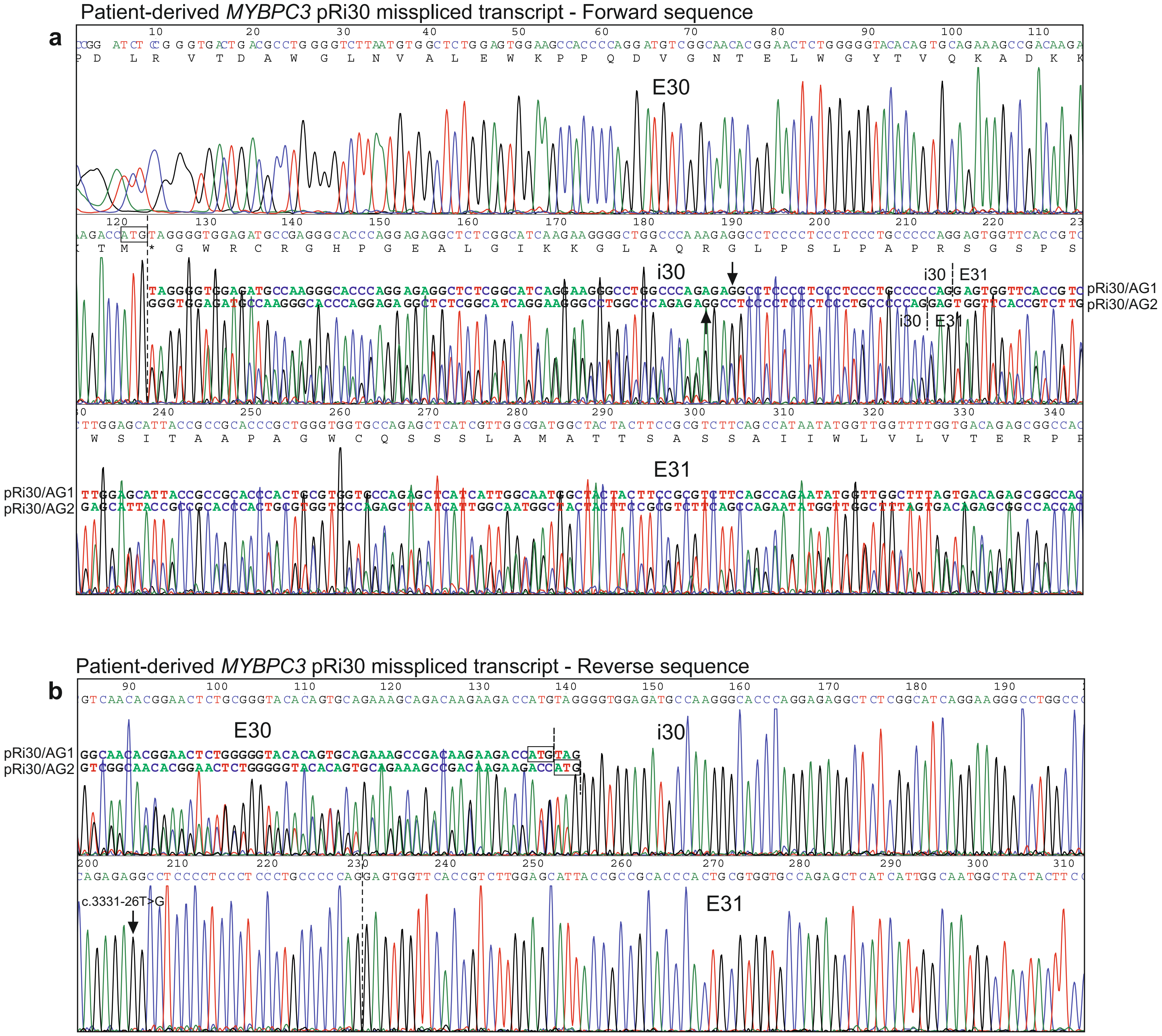

Supplementary Fig. 14. DNA sequencing chromatograms of the purified 420-bp RT-PCR band amplified from patients' RNA corresponding to the misspliced *MYBPC3* mRNA forms pRi30/AG1 and pRi30/AG2. Forward (a) and reverse (b) sequencing chromatograms derived from patient III.4 are shown. Identical sequences were obtained from patient III.6. The analysis of both sequences indicated that two similar-sized RT-PCR products were co-localized in the 420-bp gel band, resulting in overlapping chromatogram peaks in the middle part of the sequence. The structures of these patient-derived misspliced *MYBPC3* transcripts are identical to those previously obtained by the minigene splicing assay, described in Supplementary Fig. 8. Thus, Figure annotations there also apply here. In this case, the overlapping *MYBPC3* transcripts from patients' RNA correspond to RT-PCR products of 420 bp (pRi30/AG1) and 417 bp (pRi30/AG2), resolved as a single band of about 420 bp by agarose electrophoresis separation (see Fig. 6).

Patient-derived *MYBPC3* normal transcript E2-E3-E4 (RT-PCR product length: 292 bp)

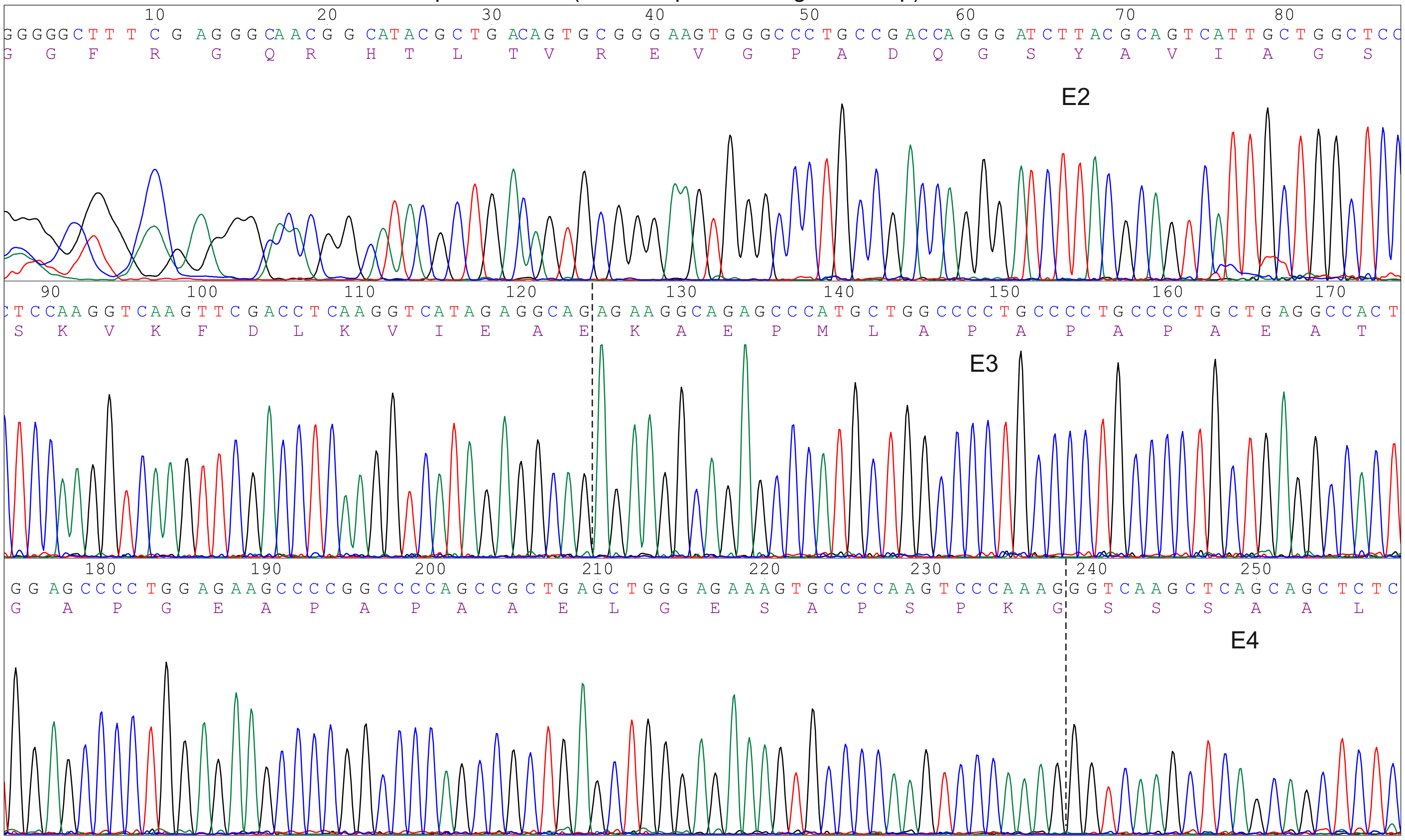

Supplementary Fig. 15. Sequence analysis of patient-derived *MYBPC3* RT-PCR product of exons 2-3-4 (E2-E3-E4). Sanger sequencing chromatogram of the 292-bp product obtained by RT-PCR of blood total RNA (III.7, carrier of the *MYBPC3* c.292+177C>T variant located in intron 2) with the use of a forward primer in exon 2 and a reverse primer in exon 4. The cDNA sequence corresponds to normal *MYBPC3* mRNA, indicating that intron 2 was removed and exons 2 and 3 were normally ligated in the presence of the c.292+177C>T intronic variant. Identical cDNA sequences were obtained from the other analyzed carrier (III.1) and non-carriers (III.4, III.6) of the *MYBPC3* c.292+177C>T variant. In addition to the expected RT-PCR product of 292 bp, no other splice forms were detected in the targeted mRNA region by this splicing assay. The exon-exon junctions are marked with vertical dashed lines. The deduced amino acid sequence is shown below the cDNA sequence as translated by the Chromas software.

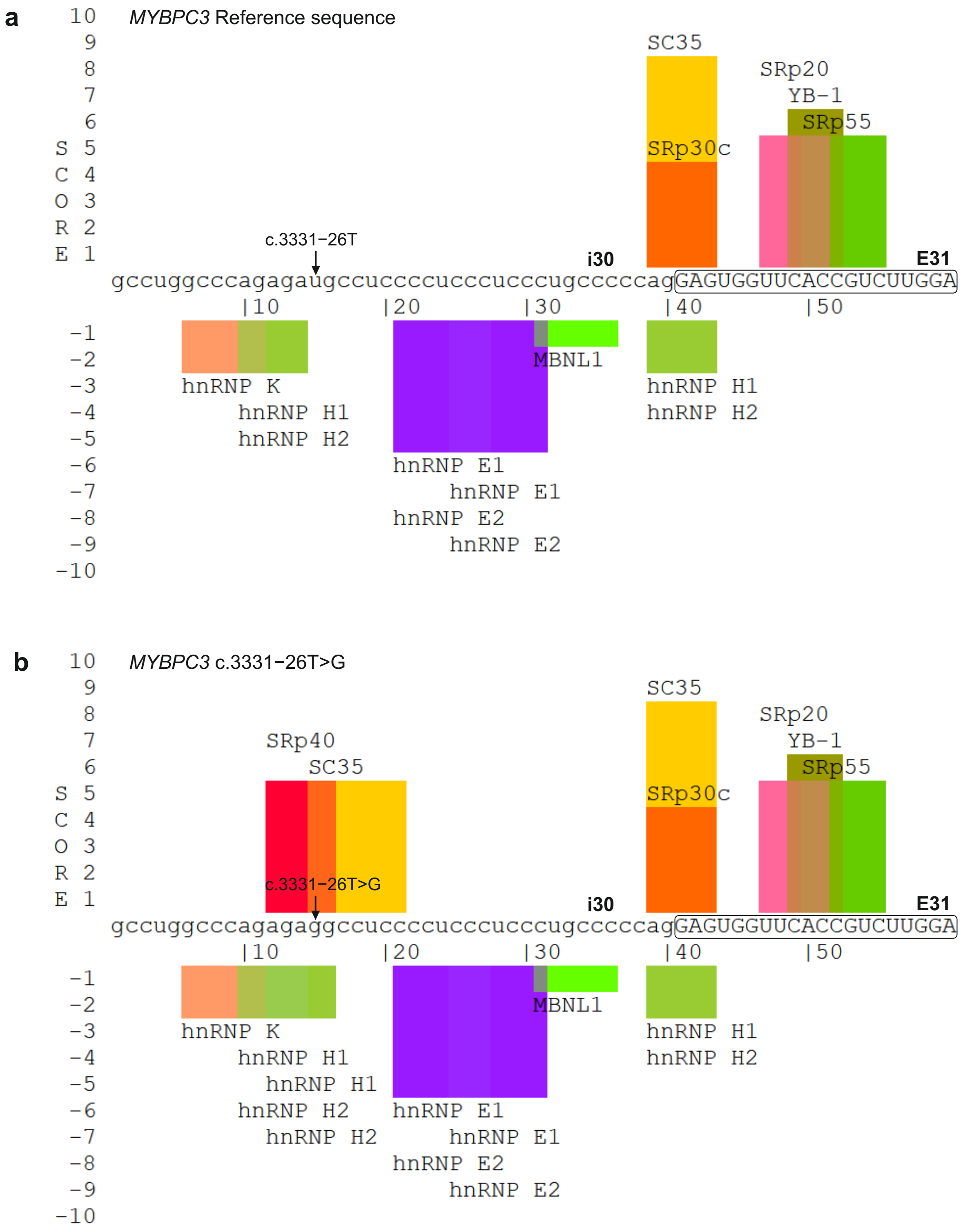

Supplementary Fig. 16. SpliceAid analysis of RNA target motifs bound by splicing proteins in the *MYBPC3* reference and c.3331−26T>G sequences. The junction between intron 30 (i30, last 40 nucleotides, lowercase) and exon 31 (E31, first 20 nucleotides, uppercase) from reference (**a**) and c.3331−26T>G (**b**) *MYBPC3* sequences were used as input. Graphic output histograms of each target RNA sequence are represented by a colored bar whose height is proportional to each sequence's respective binding affinity (score). The software assigned a positive score to target sequences that facilitate exon definition and a negative score to target sequences that facilitate intron definition. No binding sites are present only in the first reference sequence. Binding sites for SRSF5 (splicing factor arginine/serine-rich 5, also known as SRp40) and SRSF2 (splicing factor arginine/serine-rich 2, also known as SC35) are present only in the second mutated sequence: AGAGG for SRp40 (score +5) and GGCCUCC for SC35 (score +5); the affected nucleotide is underlined.

Supplementary Fig. 17. Annotation of cryptic splice-altering intronic variants in the *MYBPC3* gene. The 46 splice-altering single nucleotide substitutions listed in Table 1 are annotated in red. Genomic coordinates correspond to the Reference Sequence NC_000011.10 (GRCh38). Exon (E) and intron (i) nucleotides are shown in capital blue and lower-case black letters, respectively. The first and last exon nucleotides are shown in light blue. If a variant creates an active GT or AG cryptic splice site, the corresponding dinucleotides are highlighted in grey in the reference sequence. Splice-altering variants can also weaken natural splice signals without creating competitive splice dinucleotides. Start and stop codons are boxed.

```
attcatctat atttagcagg tggctggaca ggaggcagat aagcagagcc  47352753
tggggagggg ggaggtcccc atatatagtg ggaaggacag gaccccactc  47352703
AGTCCCTCTT TGGGTGACCT GTGCCTGCTT CGTGCCTGGT GTGACGTCTC  47352653 E1
TCAGGATGCC TGAGCCGGGG AAGAAGCCAG gtagctttag gactggggtt  47352603
gggtctaagt gtgggagcag gggggtgtct acaattgggg agcagggcta  47352553
ggagggagtt cttgaggggg ttgacgagga cgtggcctct gagccccagg  47352503
acaggggcag ccagtcctcc agggtccttt tttgattctc ggtttctttt  47352453
tcccatccct ctgatgagag gtgaaggtaa acccaaggtc agggacgcag  47352403
agaggggaca cagccaagac aggaagcacc cggggtcaac ccattctgag  47352353
aatgcaaagg gacccctccc ctgtcccatt gccttcacct tccacagcaa  47352303
ggttccccgc ggacagagcc cacgtttagt atggagctga gccaggctca  47352253
cggcatgcct tcagaccagt ctctacccac tatgggtctc agtctcccaa  47352203
cctggggtgc cgggggtggt ggtggcattg ggttggttcc tcctggctgt  47352153
gacattatct gcttcatgag cctgtacggt gtggccccca tgttgagggt  47352103
gacacagccc ctcttctctg agagaggaca gtcaggagcc cctgaaccca  47352053 i1
ggccaagggc agggaaaatc acagcccagg gacctgggcc tgggctgtgc  47352003
ccttatgaag gttgtccagg gcagagatcc aggtccccat cccccagcgc  47351953
cctcccaggc cagctttatc tcagttgctg atacccagcc tgaggtcctt  47351903
ggaggacaaa tttggccaca ttccaagtag gcagacttcc ccgcccccctg  47351853
ccaagggccc gaaatggttg ggggaaaggg agctgggttc atcacgggtg  47351803
tctccaagca gggaaagtgg acacatgcag aaaaagtctt gttgggaggt  47351753
caggaccaca gccctggctc tcccgactgc tagctgtgtg atcttggagg  47351703
agccgcttag cttctctgag cctcagtgtc ctcctctgag aaatgaacag  47351653
aataactgca actacctcag agaactggta gagtattacg tgaggggatg  47351603
catagaaagt gctagcacag tatttactga gaggggctgc aggggcgggg  47351553
tgcacgctcc aaccaggggc cgctgtagcc tccacctggc ccttcagTCT  47351503 c.26−10G>A
CAGCTTTTAG CAAGAAGCCA CGGTCAGTGG AAGTGGCCGC AGGCAGCCCT  47351453
GCCGTGTTCG AGGCCGAGAC AGAGCGGGCA GGAGTGAAGG TGCGCTGGCA  47351403
GCGCGGAGGC AGTGACATCA GCGCCAGCAA CAAGTACGGC CTGGCCACAG  47351353 E2
AGGGCACACG GCATACGCTG ACAGTGCGGG AAGTGGGCCC TGCCGACCAG  47351303
GGATCTTACG CAGTCATTGC TGGCTCCTCC AAGGTCAAGT TCGACCTCAA  47351253
GGTCATAGAG GCAGgtaaga tcctgatcag ccttccctga ggtttgggct  47351203
gctgggggag gggcagccca gcgactctcc atccatccag ggaacaggag  47351153
gtgctttcac actttcttgc ctttgctgtg gctgtgcccc ccgccaggaa  47351103
cgccctttcc cccttttctg cacatgacct ctctgagtac ccccatgtgc  47351053
catcctggct tgttccagcc atggggtagg tgggagagat gaacaagaca  47351003
caaccccact ctccccctgc agacctccct ggggagatga ccctgcacag  47350953
acggccggga agccccagga gctgtggatg aggggagtgc agcccagtct  47350903 i2
gaggggctgc tctaaggctg ggcagagca gggatggacc tggagctgtt  47350853
cagggcagat gcagaggaca tgggaactga tggcctggga cggggaggag  47350803
aatgtgagag gcaggctggc tctccagctc agcatgagct gcctctgagg  47350753
ggcccaggca gcgggaggac agccatggca gactttcctc atccacagcg  47350703
ggctcatggg tccaatgctc aggagagaga gggtggaggg gtctctggtt  47350653
agtggctgca ccacgactcc catctgatcc cctccagAGA AGGCAGAGCC  47350603
CATGCTGGCC CCTGCCCCTG CCCCTGCTGA GGCCACTGGA GCCCCTGGAG  47350553 E3
AAGCCCCGGC CCCAGCCGCT GAGCTGGGAG AAAGTGCCCC AAGTCCCAAA  47350503
Ggtgagaggg gctgggcagg gaggggcagg atccgtgggt agggcgtgtc  47350453
cagggcaggt ctcaaaagcc tttgctgggc caggtgcggt ggctcaggcc  47350403
```

```
           tataatcccc agtactttgg gaggccgagg tgggcggatc atgaggtcag  47350353
           gagattgaga ccatcctggc taacatgatg aaaccccatc tctactaaaa  47350303
           atacaaaaaa attagccggg cgtggtggcg ggcacctgcg gtcccagcta  47350253 i3
           acttgggagg ctgaggcagg agaatggtgt gaacctggga ggcggagctt  47350203
           gcagtgagcc gagattgtgc ctctgcactc cagcctgggt gacagagcaa  47350153
           gactccatct caaacaaaca gaaaaagcct ttgctcacag GGTCAAGCTC  47350103 c.407−5C>T
           AGCAGCTCTC AATGGTCCTA CCCCTGGAGC CCCCGATGAC CCCATTGGCC  47350053 E4
           TCTTCGTGAT GCGGCCACAG GATGGCGAGG TGACCGTGGg tgagtgtgag  47350003 c.505+5G>C  c.505+6T>G
           ctgctgtgcc cagcattggg gtgggaaggg ggggcagcag gacactcccc  47349953 i4
           aagccgggcc tgtcgcccct gcctttgcag GTGGCAGCAT CACCTTCTCA  47349903
           GCCCGCGTGG CCGGCGCCAG CCTCCTGAAG CCGCCTGTGG TCAAGTGGTT  47349853 E5
           CAAGGGCAAA TGGGTGGACC TGAGCAGCAA GGTGGGCCAG CACCTGCAGC  47349803
           TGCACGACAG CTACGACCGC GCCAGCAAGg tgggtccacc ggggtcgtgg  47349753 c.654+5G>C  c.654+5G>A
           agacacggag agaggggaca tggagagccc tagaaggcac aaggcacaca  47349703
           gagggcacca tgagacccag aggcacacac gagctgcaag caaggggtgt  47349653
           ggggaatctg ggaggcaggt ggaggggcca ggggcccaca gggagatccc  47349603
           ggaggcctgg gggccaggga gtgcgatgag gctcatggga gcactgtcgg  47349553
           ggacctggga gacttggtgg acatgatcac ctcaaggggg cacctaataa  47349503
           ggtttggccg gggtgggggg gtcgcccagg gtggtcagga agcctggagg  47349453
           acactgtgaa gcatgggggc accagctagt ggggttgtaa agcccctgga  47349403
           tgggtgagta tcagggctaa gggctcaagg gactctcagg gattaaccag  47349353
           aacaattcct cattatacag acacggaaac caaagcttga gaggttaagt  47349303
           ggctgctcca aggtcactta gacataggcc tagaacctta agccttcgtt  47349253
           ttttgtgttt tgtttttttc cgccctgccc accagaacct taaggcttaa  47349203
           gtccccttgt ttgttcactc caagcctctg cttttcccaa ccttccccag  47349153
           tcctgggcta ggcaaggggg cctctagcaa gagaggccaa catgttctgc  47349103
           tgtgcggggc tgtgcattgc acaaaagcct gtatttcacc acaaccatgt  47349053 i5
           gccagcagat ggcaatcatg ggtttcgaga tagtcccctt tctctgtaca  47349003
           aaaactctgt atgggttaac taaagcctga gggtgcgaag atcgatgagc  47348953
           atttgtcact cccagcctcc tttaaatata tatatatata tataatttga  47348903
           gacagggtct cgctctgtcg ctcaggctgg agtgcagtgg ctcaatctcg  47348853
           gctcactgca acctccacct cctgggttca agcgattctc ccacctcagc  47348803
           ctcccgagta gctgaaacta gaggtgtgca ccaccatact tggctaattt  47348753
           tcgttatttt tagtagagac ggaatttcac cacgttggcc aggctggtct  47348703
           ggagctcctg gtcttatgtg atccgcccgc ctcagcctcc caaagtgggg  47348653
           attacaggcc tgagccaccg cgcccggcca ctcccagtct cctttaaggg  47348603
           tgcggagcct tgtctcccgg cccctggtgt cccctgacgc cccgtccctc  47348553 c.655−25A>G
           catgcacaca gGTCTATCTG TTCGAGCTGC ACATCACCGA TGCCCAGCCT  47348503 E6
           GCCTTCACTG GCAGCTACCG CTGTGAGGTG TCCACCAAGG ACAAATTTGA  47348453
           CTGCTCCAAC TTCAATCTCA CTGTCCACGg tgagggggcc ctggtgtctg  47348403
           tcctgggctc gggctcccca tgggtcctgg tctcctacct ccttttccca  47348353
           acactaagga ggatgcctcg tcccatccag acatgagtgc tggccacgtg  47348303
           cccagtgctg cacacacagg gtgtgagaga aaccccaagg cttgcagggt  47348253
           aggcgtgggg gcttagggct gagtccgggt ctcatctggg tgggactctg  47348203 i6
           ttttatcatc ttggtgtcac ggctcctggc ccatggcctg gcactgtctg  47348153
           aatgtttggg cgatgggtga aggtgagttg agagatgggt gggaattggg  47348103
           gacaataaac cgtcccacct gcctggtacc ttgaccctgg gaaaggctgg  47348053
           gaaggtgaga tcctggggca gatgcccagc ctcatggggt catgaatggg  47348003
           caagtctgtg aatactcaga ggccgccccc agggcagggc ttctcaaacg  47347953
           gccccctctg aagccccttc ccccatctct ccaccctttg aacccagAGG  47347903 E7
           CCATGGGCAC CGGAGACCTG GACCTCCTAT CAGCCTTCCG CCGCACgtga  47347853 c.821+3G>T
c.821+5G>A gtggccatcc tcagggcctg ggggaggcca gtgctggagt ctgaggccct  47347803
           tcagggtctc gactgggggt cagggctggg gatgatttgc ggggcggagc  47347753 i7
           tgaagtcagt gggggctgca ggggagagca agcttcccag gcaggtgagg  47347703
           atactgagtc taacccccac agGAGCCTGG CTGGAGGTGG TCGGCGGATC  47347653 E8
           AGgtacccct gccccaggcc ctacctgcac catccgcaga cccccagggg  47347603 i8
           tctgagacgg gtgggagccc aatctggcta gtgtcccttt ctccctcccc  47347553
           actccactcc catcctgctc ctaatccctt tccagtccct cactgcagcc  47347503
           tcactggggg tccctctcca tagTGATAGC CATGAGGACA CTGGGATTCT  47347453 E9
           GGACTTCAGC TCACTGCTGA AAAAGAGgtg agtcctgggt ggcagttcct  47347403
           ggggcagctg gtgaggcctg gctccaaatc ccagcactgt ggtttgccag  47347353
```

ctgtgtgacc ctgagcacaa cactgcacct ctctgagttg gtttccttgt 47347303
ctgtcgaggg gatgatccca gtgtggaagg agttaaaggg ctctgcagac 47347253 i9
attatgtccc gtcaacagtc atcctcacag tgtcccccac ggccactcca 47347203
cacttgaggc tgcaaaagct ccctccgtaa actccgggga cccagaagga 47347153
acagagagag ggtccccaga gctgcagggt ctaccaggtc ggcccaactg 47347103
acttatgttc tctctgccct ctctctccct ctccccc**g**gc ccctccatta 47347053 c.906−36G>A
c.906−8T>C tggctgctgc tgctg**t**ggcc cagAGAgtaa gaatcggggt ctgggtgttt 47347003 **E10**
gggggcggca gggcgctggt gggtgcagag gggattcccg tcccctctct 47346953
gagaggctgt ggtgagagac tcagagcagg gtgcggctcc ccacggacag 47346903
gggtgagtct ctgtgccttg ggcttctcct gccacccacc tatctgtcag 47346853 i10
agcttaggag ggataagaaa aagtaatgca cacaggaggt cctgccagag 47346803
cctccggagc agcatgggtg ccccacaaca cagggagtgc aagtgcggaa 47346753
aatagggagg aagtaaaggc ccatgggtgg ggtccaggtc tttgagggaa 47346703
ggtggccata cctctcatgt gccacctacc ctttctccca cccccacccc 47346653
tggagcagCA GTTTCCGGAC CCCGAGgtg**a** gtgcccaaca tagcacagca 47346603 **E11** c.926+4A>G
ccctcatcac ccctaattct gccaggagcc cctccagatc cccagcccct 47346553
cttcagctcc cttggccccc agtcctgcct ggcctgggac ccagagcagc 47346503 i11
tccagctgcc ccaccagaag ggaagggca gagggacagg ggtggctaca 47346453
gctccttggt cctgggccca ggaagccccg cccaggggc tgcagtcttg 47346403 c.927−10C>A
cccccggcca cagcctagac tg**cgg**gacac agGGACTCGA AGCTGGAGGC 47346353 c.927−9G>A
ACCAGCAGAG GAGGACGTGT GGGAGATCCT ACGGCAGGCA CCCCCATCTG 47346303 **E12** c.927−8G>A
AGTACGAGCG CATCGCCTTC CAGTACGGCG TCACTGACCT GCGCGGCATG 47346253
CTAAAGAGGC TCAAGGGCAT GAGGCGCGAT GAGAAGAAGA GCACAGgtta 47346203
gcccttcctc agaggggaga ggagagggca caggctagcc cttccctaca 47346153
caggagagga gagggcatag gttagccctt ccccacaggg gagaggagag 47346103
ggcacaggtt agccccccac ctcgtccaca tacatggaaa gagcggagtc 47346053
cggcttagca cagagactca caggaaagaa gagcccaggt taacccctcc 47346003
ttgtgtagaa aaagccaaga atgcccaggt tagcccctta ccacagagat 47345953
agaagagaag agcaaaagtt aggccaggac agccacaagg aaaaggacag 47345903
agctcaggtt aaccctcctc accaaggaaa aatcaggcta cactttcttt 47345853
ctctaccctc ttctgaaaag aaatgaagcc cggttaaccc tctccacacc 47345803
caaaagaaaa gggaagaggc ccgctaagcc tggagagcca cacacagg**c**a 47345753 c.1090+453C>T
aagaaaagag cctggctaat ctttctctca ggcaaacaaa gcaggagggc 47345703
ttgggttaac taccctcacc cccaggcaga tggaaagagg agagcccagg 47345653
aatagccctt ctccactgag gggttgacaa gggcgggtgt gagccgggaa 47345603
agcaggggtc tcacatgcct tctccccacc atgtctgggt ctagcacagg 47345553
gcctggcaca cacctcgagc gctctataaa caccgactga atggaggggt 47345503
cagcttccat gcaggatatg ggagacaagc ccctcccaaa gcacacacac 47345453 i12
aaaacacaca cgatcatggg gacggggaga ggagctggtt agtccctccc 47345403
tgctgacctc ccccaggccc cttatccctc tgccctgcct ccctgttgtc 47345353
tcagcctcac tgggtttccc atcaccccat cgctaggtgg cctggccctt 47345303
accggcaccg tggccccatt taagccaggg atcccctgaa tgccaccaat 47345253
gttcctctgg gttaactcct gaactgctga gcccagaggg caccaagcaa 47345203
aggactgtag gtgcccaagg aggaaaagat ggaggctggc aaggcagatg 47345153
gggacctccg agggtagggg tgtcttccag gaatagcaac aacagtaaaa 47345103
agagcaaatt gctggccagg tgcagtggct cacgcctgta atcccagcac 47345053
tttgggaggc cgaggcaggt ggatcacctg aggttaggcg ttcaagacca 47345003
gcctgaccaa catggtgaaa ccccgtctct actaaaaata caaaattacc 47344953
tgggcatggt ggcaggcgcc tgtaatccca gctactccgg aggctgaggc 47344903
aggagaatca cttgaacccg ggaggcagag gttgcagtga gtcaggattg 47344853
tgccattgca ctccaacctg ggtgacagaa tgagactcca tctcaaaaaa 47344803
agtgagtgag ttccttccta agccagccac taggtgcttt atgtaattat 47344753
ctcatttaat tctcgaaaca acactatgat ggaaggacta ttaccatttc 47344703
catgttactg aggaaaccga ggcacagagg gtttaaggaa ctagcgcaag 47344653
gtcacagagc acccagcagt agatctctgc ccctccgcag ctgactccag 47344603
gcccagccag agtatttgag gactcagggg tccttctctg ctcggtggat 47344553
ggagcccccca cttcctccct gggctcagca gcagcaacat gggcttttac 47344503
aaacacggcc gcaagaggag gcattgttgt ggagcagggc tgggctcaag 47344453
acccagctct gccacacacc tgctgggctg gtggcattgg gccagtcgcc 47344403
tctctgagcc ctatttcccc cactgcagca gcatggggac caccgcactg 47344353
gccctggggg ctgagatggg agtgagagtg ctgggcacag agggctgcta 47344303

ggtgggagcg cctgggaaga gtacacaact caggggttgc tgagagaaga 47344253
gaaggcacag ctgtcccagc cccagttctc ctgacaagct tctgtctttc 47344203
c.1091−575A>C tggaagcccc taacccaatc cctgagctga gcaggtggag aggaccccaa 47344153
ggaagacaga aggacaaggt cagacagaca gagacaagga ggtgggttga 47344103
cacatagaca cagataaaga aatgaagaca ccatgcctgt aatcccagca 47344053
ctttgggagg ctgaggcggg cgcatcacct gaggtcagga tttcaagacc 47344003
agcccggcca acatggtaaa accccgtctc tactaaaaat acaaaaatta 47343953 i12
gctgggcgtg gtggcacatg cctgtaatcc cagctactcg ggaggccgag 47343903
gcaggagaat cacttgaacc tgggaggcag aggttgcagt gagccaagat 47343853
ggcgccattg cactccagcc tggccaacag agcaagactc tgtccaaaaa 47343803
acaaacaaac agacaaacaa acaaaacaaa aagaaataga gacacagaga 47343753
agtggaagcg gggcggcaca gaggggattg gaggggggga cctgggcccc 47343703
agccacagcc acagtagctt ggcctggggg agcagggtgc gggcggtggg 47343653
gtggccgggg ctgaggggtg gtgctcagCC TTTCAGAAGA AGCTGGAGCC 47343603
GGCCTACCAG GTGAGCAAAG GCCACAAGAT CCGGCTGACC GTGGAACTGG 47343553 **E13**
CTGACCATGA CGCTGAGGTC AAATGGCTCA AGAATGGCCA GGAGATCCAG 47343503
ATGAGCGGCA Ggtgcagcct ggggtgggga ggggggctcg ggtggaggtg 47343453
gggaccccca tagccttgcc tcctgcccct ctccacatgc gtatctctga 47343403 i13
ctcggtgtgg ctctcagccc catctctctg ggcctaattt cccatccttt 47343353
c.1224−80G>A tgctcctgcc ggtccctctc tctctctcct tttgtctcgg gctcacttcc 47343303 c.1224−52G>A
c.1224−21A>G tcccgtcggg aacacttcaa cggccccttc tgttctacag CAAgtaagtt 47343253 c.1224−19G>A **E14**
cccctctgga tggcttgggg tggggggcac agggattatc atggggcgca 47343203 i14 c.1227−14C>A
ctgggcccgg aggggtgtcc gcagctttcc tgccacttcc ctgcggcccc 47343153 c.1227−13G>A c.1227−9C>A
cacccagGTA CATCTTTGAG TCCATCGGTG CCAAGCGTAC CCTGACCATC 47343103 **E15**
AGCCAGTGCT CATTGGCGGA CGACGCAGCC TACCAGTGCG TGGTGGGTGG 47343053
CGAGAAGTGT AGCACGGAGC TCTTTGTGAA AGgtgggcct gggacctgag 47343003 i15
gatgtgggaa cctggggagg agatggcctc aggggagcca accctcatgc 47342953
tcaccctgcc tggacagAGC CCCCTGTGCT CATCACGCGC CCCTTGGAGG 47342903 **E16**
ACCAGCTGGT GATGGTGGGG CAGCGGGTGG AGTTTGAGTG TGAAGTATCG 47342853
GAGGAGGGGG CGCAAGTCAA ATGgtgagtt ccagaagcac ggggcatggg 47342803 c.1457+3G>C c.1457+5G>C
tgttgggggc atctgcccag aagaggccac agcacttgcc acccacccac 47342753 i16
ccggctagGC TGAAGGACGG GGTGGAGCTG ACCCGGGAGG AGACCTTCAA 47342703
ATACCGGTTC AAGAAGGACG GGCAGAGACA CCACCTGATC ATCAACGAGG 47342653 **E17**
CCATGCTGGA GGACGCGGGG CACTATGCAC TGTGCACTAG CGGGGGCCAG 47342603
GCGCTGGCTG AGCTCATTGT GCAGGgtgag cctggctggg ggggcacatg 47342553 c.1624+4A>T
aggctttagg gcttggaccc ctcagccccc accccaccta gccctgtagg 47342503
ggagcaggca aacctgggct caaggcctct gtgaccttgg gcctttgaca 47342453
gcctaaacct catccttctc atctctaaaa tgacgatgct gaggtgaccg 47342403 i17
ctccagaggg tactgagagg gtcatacggg cctggtgagc atcaccaccc 47342353
ctcagacact tgaggttcct tatgtgcact gcaccatcac aggcaaacat 47342303
ggcacacaca ggcacacgtg ttttcacatg cccacatgca cccagacacg 47342253
tgcgcaccag cgcccatggg cccacacgcc ctccacaggg attcacgcca 47342203
cacccacaca ccctcccgag ctcaatggct ctgccctgcc ctgcagAAAA 47342153 c.1625−7C>A
GAAGCTGGAG GTGTACCAGA GCATCGCAGA CCTGATGGTG GGCGCAAAGG 47342103
ACCAGGCGGT GTTCAAATGT GAGGTCTCAG ATGAGAATGT TCGGGGTGTG 47342053 **E18**
TGGCTGAAGA ATGGGAAGGA GCTGGTGCCC GACAGCCGCA TAAAGGTGTC 47342003
CCACATCGGG CGgtgagtgt gcagggcagg tggatgggac aggtggagac 47341953 c.1790+5G>A
tgagatggag acagagagag acacagggag aaacagagag agagagataa 47341903
agacaggtgg acagagaggc agatgcacag agacaggaca gagactgaga 47341853
cagagaccca cagagaggga gaaacagaca gacccaaaaa gacaaagaga 47341803
cagaaagaca gaaatagttc tatagagaca gaaagatatg cacagggagg 47341753
cacacacata ggagagttcg tcagagacag agaagaacag agggacagag 47341703
acaaagaggc agcagggaga gacacggacg gggtcgagcg ctccccagca 47341653
ccctccccca gcttgtgacc agggcagggc aggaggccac caaggaggcc 47341603
ggctctggcc agaggccctc actgcctctc ccgccctctt gctcatccct 47341553 i18
ggagcaactg ggctggccgt gctgggccca agttcacaga gattaatgag 47341503
acccagcccc tgcccagtct ccaagggccc agccaaggag atgctttgat 47341453
gtagaaatca cttttacgct gcctacctct gaaggcacat taaaaagctg 47341403
gaaaattaga taatatagaa aaaaaaaggc atttttaaaa atcagaatac 47341353
caacaagcca ggacaaggtg agggcctcct gggaccctgg ctggggtatc 47341303
tggcaaggcc aggggtgtgt ggcccagtgg ggtcccctga gccactgctc 47341253

```
              ccctgcagGG TCCACAAACT GACCATTGAC GACGTCACAC CTGCCGACGA  47341203 E19
              GGCTGACTAC AGCTTTGTGC CCGAGGGCTT CGCCTGCAAC CTGTCAGCCA  47341153
c.1897+5G>A   AGCTCCACTT CATGGgtgag cctgctccag ggtagggtgg gtcggggcag  47341103 i19
              tggggccagg agcccctgtc actgggccct gcctttgccc ccgtgctact  47341053 c.1898-23A>G
              tgctcttcct tctcttgcag AGGTCAAGAT TGACTTCGTA CCCAGGCAGG  47341003 E20
              gtaagtcttg gggcccctga gtcttggttc tgctcacttt cccgcaaccc  47340953
              acccacgcag gcctcccctc cactcacaga gggaagagca aggatcaaaa  47340903
              gtgggggtcc tggggcccag cttcagcttt gccactcatg accatgggtc  47340853
              aattataatc tctgtgagcc tcagtttcct cttctctaaa atgggaaccc  47340803
              tgatagtgcc acctcatagg atagttataa agatgaggtg acatgatgta  47340753
              tcagacctgt caagtgctgt cacatgtgtg actgttatta ttacatcttt  47340703
              tcttcttctt cctttttttt tatttttatt ttttttgaga cggagtctcc  47340653
              ctctgtcgcc cacgctggag agaagtggcg cgatctcggc tcactgcaag  47340603 i20
              ctccgcctcc caggttcaca ccactctcct gcctcagcct cctgagtagc  47340553
              tgggactaca ggcgcccgcc accatgccca gctaattatt ttttgtattt  47340503
              ttagtagaga cggggtttca ccatgttagc caggatggtt tcgatctcct  47340453
              gatctcatga tctgcctgcc tcggcctccc aaagtgctgg gattacaggc  47340403 c.1927+600C>T
              gtgagccact gcgtccagac tgctttgctt tgtttaactg agggcagatt  47340353 c.1928-569G>T
              cctgatttat ttggccaggg aacctttttta atgtgtgcat gtgtgcgtgc  47340303
              atgtgtgcgc gcgtgtgtgt gtgtgcatgt gtctgtgtgt gtatgtatat  47340253
              atgtgtgtct gtgtctgtgt gtgtgcgtgt atgtatgtgt atttgtgtct  47340203
              gtgtgcatgt gtgtgtctgt gtgtgcatgt gtgtatgtgt atgtgtgtct  47340153
              gtgtgtatgt gtatatgcgt gtgtgtgtgt gtattgtgtg tgtgtgtatg  47340103
              tgtgtggcaa gacactgatg ttcccccagt tttcaaaatg ctatgctagg  47340053
              tgacctgacc tgaatattac aagcctccca gccaacgtcc tggggtgatg  47340003
              ggaatgttcc agatcttggt tggattaata attgcatggg tgtattcatc  47339953
              tgtcagaact cagcctactg tcagatctga gcataaacta tacctcaata  47339903
              caaatctaaa aaaccaataa ccaggggccc aggctcttga gcagccccgc  47339853
              cccagtgacc tgtgctcctc ctggctctcc cgtttctctg aactacattg  47339803
              tgtcttctgc agAACCTCCC AAGATCCACC TGGACTGCCC AGGCCGCATA  47339753
              CCAGACACCA TTGTGGTTGT AGCTGGAAAT AAGCTACGTC TGGACGTCCC  47339703 E21
              TATCTCTGGG GACCCTGCTC CCACTGTGAT CTGGCAGAAG GCTATCACGC  47339653
              AGgtactgtg ggtccctcct cagtctcccc atctataaga tgggtgtgtg  47339603
              gaaccagcca agtgctagga cctgctgcac accccaccca catgtgcacg  47339553
              ccctgctgtt ggctacccca tgggagagac tggtcagggg gcaggcaagg  47339503 i21
              tgggcagtgt gggtcagggg gtggaagaag cagcagaggg gcgcttccag  47339453
              gctgagtcag ctcctctgct ccctacttcc ctcctgccct gttcccagGG  47339403
              GAATAAGGCC CCAGCCAGGC CAGCCCCAGA TGCCCCAGAG GACACAGGTG  47339353 E22
              ACAGCGATGA GTGGGTGTTT GACAAGAAGg tgagtgagac tgaggtcagg  47339303
              aggaggctcg tttgtctttc atcactttct acctacgtgg tgccgagccc  47339253
              ttggcgaggt ctccatggag gtgccacact tggccgggtg tgttgctgcc  47339203
              cccagcaggg tccctgtggg gccctcacac ctcaggcaga gggtctctgt  47339153
              gaagtgtgct gatggccatc acaaggatgt ggacaccctc tcattgccag  47339103
              gaagccctca tgactgggct gctccctggc tcccttcatc ctaagctgac  47339053
              cttgaccttg accttgtgct ccctctcaac tcaggagtag tttctgagac  47339003 i22
              acttttctgc cagtgtagct gccggggtct tgctcctgcc tggcactgtt  47338953
              ggttcttctg aaggctgggg tcttgagggg tcctcatgat agctcatctg  47338903
              agaggacagg tggttgcccc aggtcccgtg acaaagctag aacccgagcc  47338853
              cctgccctca gtcggtgcca cagagatgat tttgaactag atgctgacgt  47338803
              ggatgcagtc ttcccacagg ctgtgttccc cccatggacc cccgggacat  47338753
              ctggctgttg gcggaggctg caagggcctc tggggtctga cttggatctc  47338703
c.2149-3C>G   accccaactc tgcacccccc cagCTGCTGT GTGAGACCGA GGGCCGGGTC  47338653
              CGCGTGGAGA CCACCAAGGA CCGCAGCATC TTCACGGTCG AGGGGGCAGA  47338603 E23
              GAAGGAAGAT GAGGGCGTCT ACACGGTCAC AGTGAAGAAC CCTGTGGGCG  47338553
              AGGACCAGGT CAACCTCACA GTCAAGGTCA TCGgtgaggc cggccggggt  47338503 c.2308+3G>C
              ccaagctgga gaacacagag gggcagcccc aaggagggcc catccgttcg  47338453
              ctcattcagc cactcgacaa acatcacggg catgctccct gagccgagcg  47338403
              gcaacaggac aggtttttct gcccaaaagg agccccgctc tgggggagat  47338353
              ggaggcagat gcatccagca gatttcctgc gacactgctg tacatgagcc  47338303 i23
              atgtccagag tgccgtggga gcactgagca gagagccatc cctgtgcctg  47338253
              ggggtgatgc ctttaggagg ctgagaagat ggtgaggaaa ggcatcccag  47338203
```

gcagaggaaa ctgtgtacaa aggggtggag gagggaaagg gccttttggg 47338153
tgtggagaat gaatgaaaca tgtcaggaga ggactgggtg gggttgggag 47338103
gatcagagcc aggacagggg tgaatatggg cagtgagaag ccttaaaggg 47338053
ttgacggagg gctggcgaca tgatcagaaa tgcatcctgt tgagacccag 47338003
cctggacaac acagtgagac tccatatcac taaaaaaaaa aaaaaaaaaa 47337953
ggaaaagaag aaagaaatgc attctagaaa gatctctttg gatctggggg 47337903
tggtgggggc agcctgtggc ggttagttgg agtgggaagg ggacgagcaa 47337853
cgttactcaa ggccctgagc ggggcagggc tgatgtgggt ccatcccacc 47337803 c.2309–26A>G
ccatccagAC GTGCCAGACG CACCTGCGGC CCCCAAGATC AGCAACGTGG 47337753 **E24**
GAGAGGACTC CTGCACAGTA CAGTGGGAGC CGCCTGCCTA CGATGGCGGG 47337703
CAGCCCATCC TGGgtgagtg caagggcacc ggatggaggt gtgagggcgc 47337653 i24
caaacagatc cgagggaagg tggtgtgggg atgcctgggt tccagaccag 47337603 c.2414–36G>T
agctgccacc tcccctgagc cagGCTACAT CCTGGAGCGC AAGAAGAAGA 47337553
AGAGCTACCG GTGGATGCGG CTGAACTTCG ACCTGATTCA GGAGCTGAGT 47337503 **E25**
CATGAAGCGC GGCGCATGAT CGAGGGCGTG GTGTACGAGA TGCGCGTCTA 47337453
CGCGGTCAAC GCCATCGGCA TGTCCAGGCC CAGCCCTGCC TCCCAGCCCT 47337403
TCATGCCTAT CGgtgagcct gcctggcctg gtcctgcccc ccgccccctc 47337353
cccagttaaa aacctccaat aatagcaggt gctctgcagg cagccatgct 47337303
agacactcac atccacagtc tcctttcatc ctcgactggg gatactcagc 47337253
cgcattttac agatgaggaa acaggctcca agcagttaaa tgacctgctt 47337203
gagatctccc agctggttgg ggtggagctg actctcacac tacgccgacc 47337153
cctagttcac gtttcaagcc tctctttcca tgtctacaaa atgggaataa 47337103
tgacactgcc tatctcagag agctgttagg atgatatggg atgaccaggc 47337053
aggccatctg gcatctaaca ggtgctcaat aaatgttcat tgtcccctgt 47337003
tctccacacc cttcaaccca gggttttcac ctatgggatg gggcaagggc 47336953
tgtagttagt ggaggggtct caggggcatc tgtctgtgga aagagtcctg 47336903
gaccaagact cagagatggc agaggcagct ccatggtccc cggggggcca 47336853
actggagtca tccctccctg aagcctcagc ttcctcatct gtataatggg 47336803
gtctgagtgg gcagaacagg tgttgagact gtggcagggc tcaggaggac 47336753 i25
acctgttccc actagggtgg gtgagtccgg gggcagctgc ctgtggcaag 47336703
ggcaggccaa ggacccctgg gcccagggat ggggcacagc ttgggaagat 47336653
ggcaggaatc ggggcctgtg gggagaaggg tccaggcctc tcgttttagg 47336603
gactgattcc tggtttgttc agttcttagt caactcacct aatcatacgt 47336553
gccaggaaat tcccatgttt tctgtagttt tagttttagt tttttttttt 47336503
tttttttttt tttgagatgg agtctcgctc tgtcacccag actggagtgc 47336453
agtcctggcg cgatctgggg tcactgtaac cttcacctcc caggttcaag 47336403
caattctccc acctcagcct tctgagtagc tgggattaca ggcatccacc 47336353
accatgccca gctaattttt gtatttttgt agagacggag tttcaccatg 47336303
ttggccaggc tggtcttgaa ctcctgacct caggtgatcc tcccacctcg 47336253
gcctcccaaa gtgctgggat tacaggcgtg agccaccgtg cctggctgct 47336203
gttgctgttt gtttttaagc caatctacag acatgtcttg taacagggac 47336153
aaaatattcc ttaaagtggc caaaagccag ctgaacagga agtgccccct 47336103
atgtgaccag tgggcagttc agagtctagg gcatggatct ccagcttccc 47336053
caggcttgct cagacccctc tctgacctct cctctgccca gGTCCCCCCA 47336003
GCGAACCCAC CCACCTGGCA GTAGAGGACG TCTCTGACAC CACGGTCTCC 47335953 **E26**
CTCAAGTGGC GGCCCCCAGA GCGCGTGGGA GCAGGAGGCC TGGATGGCTA 47335903
CAGCGTGGAG TACTGCCCAG AGGGCTgtga gtgtccccgc ccccaacccc 47335853
cctccccaaa ggaagaacat gctcaccttg ccattgagca gattcacctg 47335803
tagtcaagtt ttttaggccc acttttgccg aaagttgagg acacccagag 47335753
aaatatctgt cctgttttca gaagtaaaaa agcttgccta gtgaaaaaca 47335703
aatgcagagt aaagctgact gtaacacttc tttgagcagt gcgaaatcag 47335653
caactacatt ttaaaaacat atttaaggcc aggcgcgatg gcttacgcct 47335603
gtaatcccag cactttggga ggccaaggtg ggcggatcac ctgaggtcgg 47335553
gagttcgaga ccagtctgac caacgtggag aaaccccgtc tctactaaaa 47335503
atacaaaatt agccgggtgt ggtggtgggc acctgtgatc ccagctacgc 47335453
gggaggctga ggcaggagaa tcacttgaac tcgggaggca gaagttgcgg 47335403 i26
tgagccaaga tcatgccatt gcactccagc ctgggagaca agagcgaaac 47335353
tccatctcaa aaataaataa acaaataaaa atttaaaaaa acatatttaa 47335303
atcctggaga ttcctatcag aggagtgggc agtgggagtg gggtgtcagt 47335253
ggtgacacag cctgtggcct tgcctccccc tccccacccc cagGCTCAGA 47335203 c.2738–3C>G
GTGGGTGGCT GCCCTGCAGG GGCTGACAGA GCACACATCG ATACTGGTGA 47335153 **E27**

```
                  AGGACCTGCC CACGGGGGCC CGGCTGCTTT TCCGAGTGCG GGCACACAAT  47335103
                  ATGGCAGGGC CTGGAGCCCC TGTTACCACC ACGGAGCCGG TGACAGTGCA  47335053
c.2905+5G>T       GGAGATCCTG Cgtgagtgcc cctttgtgca gtcacaagac ccgccattga  47335003
                  caccccaggc ccttggtgtc cagtggaggt ggggacagtt ggggaggccc  47334953
                  agaatgctct gcccagaacg ctgggcagag acaggagggg ttggagccat  47334903
                  cgtccatgcg ctaggcctag ggaaaaagtg cagagagggt ggccaggcac  47334853
                  agtggctcaa gcctgtaatc ccagcacttt aggaggccaa ggcaggcaga  47334803
                  tcacttgagg tcaggagttt gagaccagcc tggccaacat agtgaaaccc  47334753
                  tgtctctact aaaaatacaa aaactagcca ggtgtggtgg cgcgtgcctg  47334703
                  tagtccctca actacttggg aggctgaggc agaagaatcg cttggacccg  47334653
                  ggaggtggca gttgcaatga gctgagatca cactattgca ctccagtctg  47334603 i27
                  ggtgacagag tgagacaacg tctcaaaaaa aaaaaaagtg cagaaagggc  47334553
                  tctcttggtg tatgtcagtc ccttagttct ggaacaggga aagtaaaagc  47334503
                  cacagtaccc aagctctctt ccaggagtgt ctcagctcca acaggtaaat  47334453
                  attgtctatg tagtcagatt ggtccctctg ggatccactt cccattcctt  47334403
                  tgttccttgt cccaacaaag ggtccatctt ttgtaaggat tggttctcaa  47334353
                  agctggaacg aggcagcaaa ctcaggtggt aacaacacag ggcatagggt  47334303
                  gtcaaagccc aggcttacac cctggtctat gacttactag ctgtgtgact  47334253
                  tcagtcaagt tgcttaccct ctctgatccc tgcctttga agtggtgagg  47334203
                  ctcggggaag ccagctgggc ttgggcagcc tggagttgct gtgttagcag  47334153
                  gagctaaagg aggcgcaggg cccagcaggc agcttttttc ttagctgcag  47334103
                  ctctctgggc cttgtctcaa gggaggttgg agctgtggag cccctcagag  47334053
                  ctgtggcggg ccctcactta gctacccact ctatacccac agAACGGCCA  47334003
                  CGGCTTCAGC TGCCCAGGCA CCTGCGCCAG ACCATTCAGA AGAAGGTCGG  47333953 E28
                  GGAGCCTGTG AACCTTCTCA TCCCTTTCCA Ggtgggactg gccccccttcc  47333903
                  ctgtccccca ggggagagag gctatagtgt gttgttccca tccagtggac  47333853 i28
                  tgatgtctca gggctggcct gatctgaggt accagaggct ggggtggcgg  47333803
                  ccggcccttg gagtgatcca ggttcagggt taagcttttc ctcccctcag  47333753
                  GGCAAGCCCC GGCCTCAGGT GACCTGGACC AAAGAGGGGC AGCCCCTGGC  47333703
                  AGGCGAGGAG GTGAGCATCC GCAACAGCCC CACAGACACC ATCCTGTTCA  47333653 E29
                  TCCGGGCCGC TCGCCGCGTG CATTCAGGCA CTTACCAGGT GACGGTGCGC  47333603
                  ATTGAGAACA TGGAGGACAA GGCCACGCTG GTGCTGCAGG TTGTTGgtgc  47333553
c.3190+5G>A       gtggccaagg cctccttgag ccccccttgtt tcccttccct gggctgggct  47333503
                  gctggggcca agggtggggc caggggaccc aacccacagc tcacattttc  47333453
                  cagtccactg ccccagcagg cctggccctg gttggcaggg gtggggtggt  47333403 i29
                  ccctggagcc agtgaccccc tgctcactgt caggaggcgt ggtgacccaa  47333353
                  ctgggtctgt ctcccgcagA CAAGCCAAGT CCTCCCCAGG ATCTCCGGGT  47333303
                  GACTGACGCC TGGGGTCTTA ATGTGGCTCT GGAGTGGAAG CCACCCCAGG  47333253 E30
c.3330+5G>T       ATGTCGGCAA CACGGAGCTC TGGGGGTACA CAGTGCAGAA AGCCGACAAG  47333203
c.3330+5G>C       AAGACCATGg tgagcccagg gtctggggtc cccacgtgca ccctgcctag  47333153
c.3330+5G>A       gcccggcaga cccaggccgc agctaccctt cactgtcctc accgtggacc  47333103
                  cctcaccctc cctctgctga tctgaatccc tccatagtag ggtggagat  47333053 i30
                  gccaagggca cccaggagag gctctcggca tcaggaaggg cctggcccag  47333003
c.3331−26T>G      agatgcctcc cctccctccc tgcccccagG AGTGGTTCAC CGTCTTGGAG  47332953
                  CATTACCGCC GCACCCACTG CGTGGTGCCA GAGCTCATCA TTGGCAATGG  47332903 E31
                  CTACTACTTC CGCGTCTTCA GCCAGAATAT GGTTGGCTTT AGTGACAGAG  47332853
                  CGGCCACCAC CAAGGAGCCC GTCTTTATCC CCAGACCAGg tgctgtaccc  47332803
                  tcattcttcc aaccaggggc tggggcaggg aggctgtggg aacagggaga  47332753 i31
c.3491−3C>G       ggggcctagc tttgtgtggc cctctcggta ccaagtcctg tcaccaacag  47332703
                  GCATCACCTA TGAGCCACCC AACTATAAGG CCCTGGACTT CTCCGAGGCC  47332653
                  CCAAGCTTCA CCCAGCCCCT GGTGAACCGC TCGGTCATCG CGGGCTACAC  47332603 E32
                  TGCTATGCTC TGCTGTGCTG TCCGGGGTAG CCCCAAGgta gggaacttta  47332553
                  ggcgctggtc caggccgacc aggaggcagg gctcacaggc cccgacgttg  47332503
                  agcagtcctc tccccagcct gtctcccctg ctttctctcc accttggggt  47332453 i32
                  gtatggaccg ggttcctccc cctgggcagg gccatggtac tcactcttgg  47332403
                  ttccatgttt gtttccagcc ttgggcatag tcagggactc tcgtgggacc  47332353
                  ccccgagtag aaacacagat gtgtctccct gggtccctgc caggtcccct  47332303
                  ctcagcctgg atggcttccc tccctctctt tacttattt atagCCCAAG  47332253 c.3628−12C>G
                  ATTTCCTGGT TCAAGAATGG CCTGGACCTG GGAGAAGACG CCCGCTTCCG  47332203
                  CATGTTCAGC AAGCAGGGAG TGTTGACTCT GGAGATTAGA AAGCCCTGCC  47332153 E33
                  CCTTTGACGG GGGCATCTAT GTCTGCAGGG CCACCAACTT ACAGGGCGAG  47332103
```

The two consecutive preexisting cryptic acceptor sites in intron 30 (AG1 and AG2), which were identified by experimental splicing assays of c.3331−26T>G, are double underlined. Highlighted in green and yellow are the most probably affected nucleotides by the c.3331−26T>G variant identified by SpliceAI with different windows, as described below.

SpliceAI
Window: any from 66 bp to 10,000 bp
Maximum Δ score = 0.16
Acceptor gain - position 66

SpliceAI
Window: 65 bp
Maximum Δ score = 0.10
Acceptor gain - position 63

```
GCACGGTGTG AGTGCCGCCT GGAGGTGCGA Ggtgaggagc cctcggggcc  47332053
agggcctggg agatgggaag agcgggcggc agtggggacc ctgggctttg  47332003
ctccgttgtc ctcggccaag cacctgccct ggctgcaagg gcacggaccc  47331953 i33
tgccacgccc agatgggcaa tagcttccag aaggctggga ggacacagtg  47331903
acatggcctc ctcttctgca gTGCCTCAGT GACCAGGCTG GCTCCTGGGG  47331853 E34
ATGGCCAGgt atgtaccacc cgacacccag ggcacaagtc agtggctgag  47331803
ccctggccct tggtcctggg gcagccatca gtaaatggga ggctgtaggg  47331753 i34
gccctccatt cactcgtaag ataacctgtg ttgcagGTAC AACCGGATGC  47331703
CAGCCCCGTG CCAGGAGCCT GGAGGGAAGT TGGGGAAACC CCTCCCTACT  47331653
GTTGGATGTA TGTGTGACAA GTGTGTCTCC TGTGCTGCGA TGGGGGATCA  47331603
GCAGGGCAGT TGTCGGGCAG TCCTGAGTGG GTGTTGCACA GACTGGTCCA  47331553 E35
CAGGGCTCCT GAAGGAAGCC CCTGGATCTT TGGGGTAAAA GGAGGGTGGC  47331503
CTCAAGAAAC AATGTCTGGG GACAGGCCTT TCTGGCCTGC TATGTCTTCC  47331453
CAATGTTTAT TGGGCAATAA AAGATAAGTG CAGTCACAGA GAACTCActc  47331403
ttctcaattt cggtgtgcct gttttcataa aaatgcacag gggatggcca  47331353
ggtgcggtgg ctcatgcctg ttatcccagc actttgggag gcggagg
```
